# Crossover Patterning by the Beam-Film Model: Analysis and Implications

**DOI:** 10.1371/journal.pgen.1004042

**Published:** 2014-01-30

**Authors:** Liangran Zhang, Zhangyi Liang, John Hutchinson, Nancy Kleckner

**Affiliations:** 1 Department of Molecular and Cellular Biology, Harvard University, Cambridge, Massachusetts, United States of America; 2 School of Engineering and Applied Sciences, Harvard University, Cambridge, Massachusetts, United States of America; Stowers Institute for Medical Research, United States of America

## Abstract

Crossing-over is a central feature of meiosis. Meiotic crossover (CO) sites are spatially patterned along chromosomes. CO-designation at one position disfavors subsequent CO-designation(s) nearby, as described by the classical phenomenon of CO interference. If multiple designations occur, COs tend to be evenly spaced. We have previously proposed a mechanical model by which CO patterning could occur. The central feature of a mechanical mechanism is that communication along the chromosomes, as required for CO interference, can occur by redistribution of mechanical stress. Here we further explore the nature of the beam-film model, its ability to quantitatively explain CO patterns in detail in several organisms, and its implications for three important patterning-related phenomena: CO homeostasis, the fact that the level of zero-CO bivalents can be low (the “obligatory CO”), and the occurrence of non-interfering COs. Relationships to other models are discussed.

## Author Summary

Spatial patterning is a common feature of biological systems at all length scales, from molecular to multi-organismic. Meiosis is the specialized cellular program in which a diploid cell gives rise to haploid gametes for sexual reproduction. Crossing-over between homologous maternal and paternal chromosomes (homologs) is a central feature of this program, playing a role not only for increasing genetic diversity but also for ensuring regular segregation of homologs at the first meiotic division. The distribution of crossovers (COs) along meiotic chromosomes is a paradigmatic example of spatial patterning. Crossovers occur at different positions in different meiotic nuclei but, nonetheless, tend to be evenly spaced along the chromosomes. We previously-described a mechanical “stress and stress relief” model for CO patterning with an accompanying mathematical description (the “beam-film model”). In this paper we explore the roles of mathematical parameters in this model; show that it can very accurately describe experimental data sets from several organisms, in considerably quantitative depth; and discuss implications of the model for several phenomena that are directly related to crossover patterning, including the features which can ensure that every chromosome always acquires at least one crossover.

## Introduction

Crossover (CO) recombination interactions occur stochastically at different positions in different meiotic nuclei. Nonetheless, along a given chromosome, COs tend to be evenly spaced. This interesting phenomenon implies the existence of communication along chromosomes, the nature of which is not understood. CO patterning, commonly known as “CO interference”, was originally detected from genetic studies in Drosophila [1], [2]. It was found that the frequency of meiotic gametes exhibiting two crossovers close together along the same chromosome (“double COs”) was lower than that expected for their independent occurrence. The implication was that occurrence of one CO (or more correctly one CO-designation) “interferes” with the occurrence of another CO (CO-designation) nearby.

**Figure 1:**
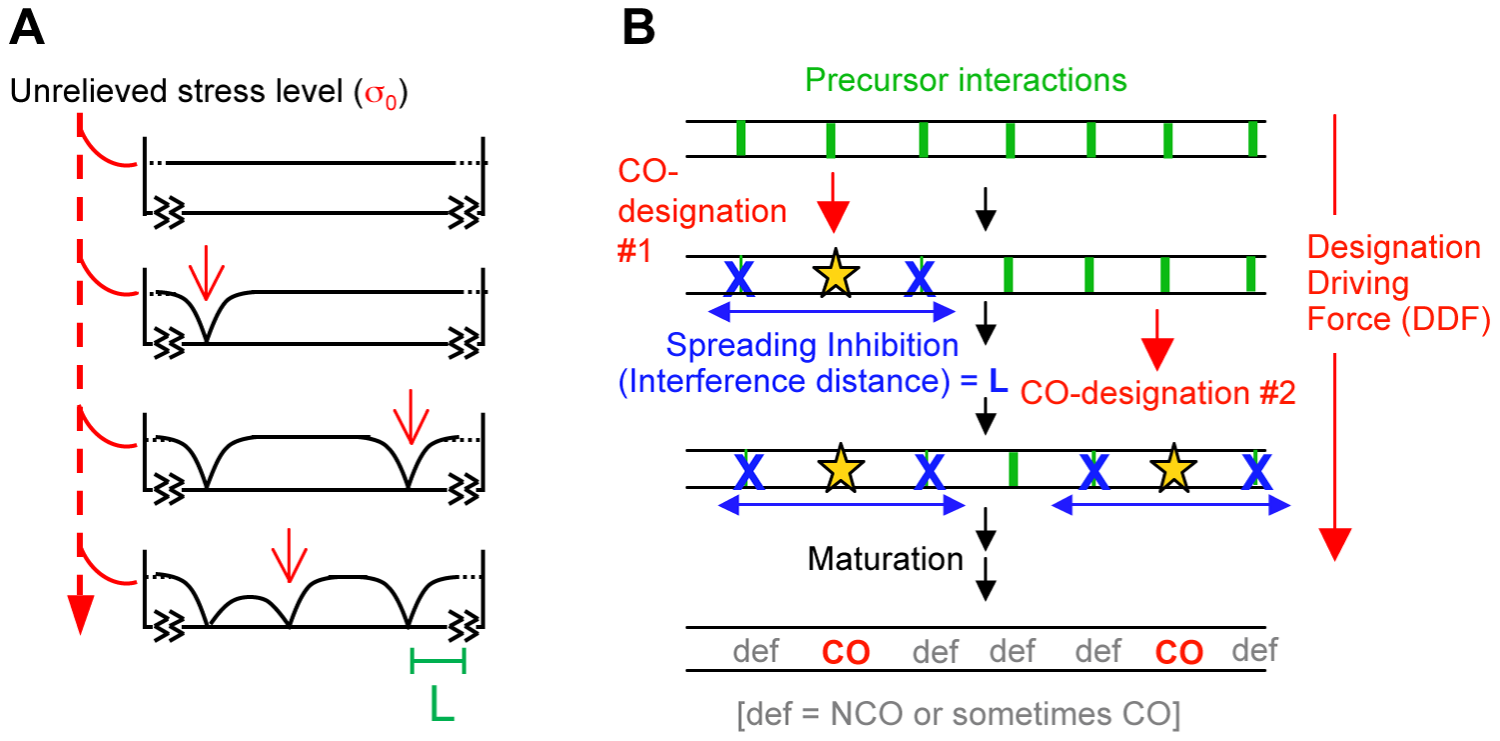
The beam-film model. (A) Beam-film model [3]. CO-designation is promoted by stress. Each stress-promoted CO-designation reduces the stress level to zero at the designation point. That effect redistributes in the vicinity, decreasing exponentially with distance, correspondingly reducing the probability of subsequent designation(s) in the immediate vicinity. Subsequent CO-designations will tend to occur in regions with higher remaining stress levels, thus giving an even distribution. More specifically: with a film attached to a beam, if the beam expands relative to the film, stress arises along the film, causing it to crack at the positions of flaws. A crack at one position will release the stress nearby (with a distance L that is characteristic of the materials) thus reducing the probability that another crack occurs nearby. Assuming a maximal possible stress level of σ_0_, if a crack occurs at an isolated position that is unaffected by any prior cracks, then the stress level at any distance “x” to either side is σ = σ_0_ (1−e^−x/L^). If two cracks occur near one another, the stress levels at positions between them is the sum of their individual effects, with additional considerations also coming into play at the ends of the beam as described [3]. (B) A generalized version of the beam-film model involving sequential rounds of event designation and spreading interference as described by the mathematical expressions of the BF model.

We previously proposed a model for CO patterning in which macroscopic mechanical properties of chromosomes play governing roles via accumulation, relief and redistribution of stress (Figure 1A) [3], [4]. In that model, a chromosome with an array of precursor interactions comes under mechanical stress along its length. Eventually, a first interaction “goes critical”, undergoing a stress-promoted molecular change which designates it to eventually mature as a CO. By its intrinsic nature, this change results in local relief of stress. That local relaxation then redistributes outward in the immediate vicinity of its nucleation point, in both directions, dissipating with distance. A new stress distribution is thereby produced, with the stress level reduced in the vicinity of the CO-designation site, to a decreasing extent with increasing distance from its nucleation point. This effect disfavors occurrence of additional (stress-promoted) CO designations in the affected region. The spreading inhibitory signal comprises “CO interference”. More such CO-designations may then occur, sequentially, each accompanied by spreading interference. Each subsequent event will tend to occur in a region where the stress level remains higher, which will necessarily tend to be regions far away from prior CO-designated sites. Thus, as more and more designation events occur, they tend to fill in the holes between prior events, ultimately producing an evenly-spaced array. The most attractive feature of this proposed mechanism is the fact that redistribution of stress is an intrinsic feature of any mechanical system, thus comprising a built-in communication network as required for spreading CO interference.

CO-designated interactions then undergo multiple additional biochemical steps to finally become mature CO products [5]. Precursors that do not undergo CO-designation mature to other fates, predominantly inter-homolog non-crossovers (NCOs).

CO patterning by the above stress-and-stress relief mechanism can be modeled quantitatively by analogy with a known physical system that exhibits analogous behavior, giving the beam-film (BF) model [3].

We note that BF model simulations can be applied to any mechanism whose effects are described by the same mathematical expressions as the beam-film case. In such a more general formulation (Figure 1B), there is again an array of precursor interactions. That array would be acted upon by a “Designation Driving Force” (DDF). Event-designations would occur sequentially (or nearly so). Each designation would set up a spreading inhibitory effect that spreads outward in both directions, decreasing in strength with increasing distance, thereby decreasing the ability of the affected precursors to respond to the DDF. When multiple designation/interference events occur, they would produce an evenly-spaced array. Maturation of CO-designated and not-CO-designated interactions ensues.

The present study adds several new features to the BF simulation program and explores in further detail the predictions and implications of the BF model (whether mechanical or general). We evaluate the ability of the model to quantitatively explain experimental CO pattern data sets in budding yeast, tomato, grasshopper and Drosophila. Our results show that the logic and mathematics of the BF model are remarkably robust in explaining experimental data. New information of biological interest also emerges. We then present detailed considerations of three phenomena of interest, the so-called “obligatory CO” and “CO homeostasis”, and the nature of “non-interfering COs”. We discuss how these phenomena are explained by the BF model and show that BF predictions can very accurately explain experimental data pertaining to these effects. Overall, the presented results show that BF simulation analysis is a useful approach for exploring experimental CO patterns. Other applications of this analysis are presented elsewhere. The current study has also provided new criteria for characterization of CO patterns using Coefficient of Coincidence analysis and illustrates both short-comings and useful applications of gamma distribution analysis. Relationships of the BF model to other models are discussed.

## Results

### Part I. Coefficient of Coincidence (CoC) Relationships and the Event Distribution (ED)

CO data sets, whether experimental or from BF simulations, comprise descriptions of the positions of individual COs along the lengths of each of a large number of different chromosomes (“bivalents”). Each bivalent represents the outcome of CO-designation in a single meiotic nucleus; the entire data set comprises the outcomes of CO patterning for a particular chromosome in many nuclei.

#### CoC relationships

**Figure 2:**
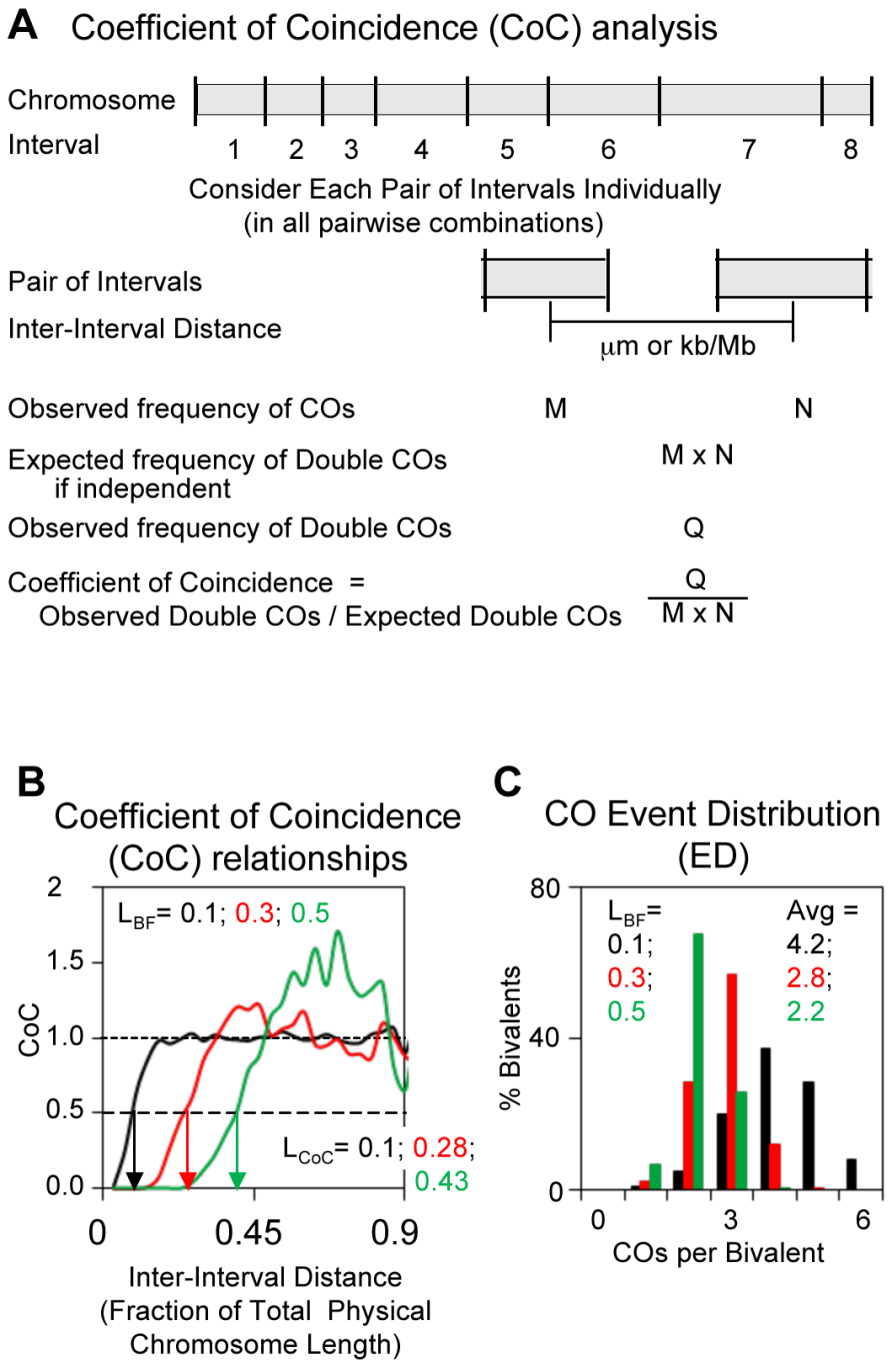
Descriptors of CO patterns: Coefficient of Coincidence (CoC) and Event Distribution (ED). (A) Determination of CoC. Interval sizes can be identical or different (Figure S1 for more details). (B,C) Data sets were generated by BF simulations at the indicated varying values of parameter (L), also called L_BF_. Other specified parameter values for the simulations are: Smax = 3.5, A = 1, cL = cR = 0.85, N = 13, B = 1, E = 0, M = 1. (B) CoC curves. Inter-interval distances given as fractions of total physical chromosome length in µm. The inter-interval distance at which CoC = 0.5 (vertical arrows) is defined as L_CoC_. L_CoC_ and L_BF_ are always quite similar in magnitude. (C) EDs. Fraction of bivalents exhibiting different numbers of COs and average values.

The classical description of CO interference relationships is Coefficient of Coincidence (CoC) analysis [1], [2]. For this purpose (Figure 2A), the chromosome of interest is divided into a number of intervals and for each interval the total frequency of COs in the many chromosomes examined is observed. Intervals are then considered in pairs, in all possible pairwise combinations. For each pair, the observed frequency of bivalents exhibiting a CO in both intervals (“double COs”) is compared with the frequency expected if events occurred independently in the two intervals. The latter frequency is given by the product of the total frequencies of events in the two intervals, each considered individually. For any pair of intervals, the ratio of the frequency of observed double COs to the frequency of expected double COs (Observed/Expected) is the Coefficient of Coincidence (CoC). If events occur independently in the two intervals, the CoC for that pair of intervals is one. If (positive) CO interference is present for the two intervals, the CoC is less than one (some expected COs have been inhibited). CoC values for all interval pairs are then plotted as a function of the distance between the corresponding intervals (defined as the distance between the centers of the two intervals).

The classical resulting CO interference CoC pattern is illustrated by an appropriate set of BF simulations (Figure 2B). When intervals are close together (short inter-interval distances), the frequency of observed COs is much less than that expected from independent occurrence (CoC<<1), reflecting “interference”. The CoC increases with increasing inter-interval distance to a value of ∼1. Additionally, because COs tend to be evenly spaced, the CoC value rises above ∼1 specifically at the average distance between adjacent COs (or multiples thereof): at these particular spacings, the probability of a double CO is higher than that predicted by random occurrence. This tendency is increasingly pronounced as interference extends over longer and longer fractions of total chromosome length.

BF simulations specify a parameter for interference distance, denoted “L” ([3]; below). Figure 2B illustrates CoC curves for simulations at varying values of L. For any actual CoC curve, whether experimental or simulated, a useful parameter for describing the strength of CO interference is the inter-interval distance at which the CoC = 0.5. We define this parameter as L_CoC_ (Figure 2B; vertical arrows). Where appropriate, the value of the BF-specified parameter “L” is denoted alternatively as L_BF_ to distinguish it from L_CoC_ (Figure 2B). Interestingly, the values of L_CoC_ and L_BF_ are always quite similar (e.g. Figure 2B).

CoC analysis provides a very accurate and reproducible description of CO patterns for experimental data sets as long as two requirements are met (Figure S1, Protocol S1). First, chromosomes must be divided into a large enough number of intervals that double COs within an interval are rare. If this requirement is not met, closely-spaced double COs will be missed. In general, interval size should be less than ∼1/4 the average distance between adjacent COs. Second, the data set must be large enough to give significant numbers of double COs. As a practical matter, where possible (e.g. for cytological markers of CO positions), interval size should be decreased progressively until the CoC curve no longer changes.

We further note that the appropriate metric for CO interference is physical distance along the chromosome. This has been shown to be the case for mouse and Arabidopsis [6]–[8]; for tomato (as described below); and for budding yeast (L.Z., unpublished). Accordingly, disruption of chromosome continuity abolishes the transmission of interference in C.elegans [9]. Experimentally, “interference distance” is defined in units of µm pachytene (synaptonemal complex; SC) length. In reality, SC length is often (or always) a proxy for chromosome length at the preceding stage (leptotene): in yeast, Sordaria and likely other organisms, CO patterning occurs at the leptotene stage and nucleates SC formation (e.g., [4]).

#### The ED

CO patterns are also reflected in the average number of COs per bivalent and the fractions of chromosomes exhibiting different numbers of COs, which we refer to as the “Event Distribution” or “ED”. As the “interference distance” increases, the distribution of COs per bivalent shifts to lower numbers with a corresponding decrease in the average number of COs per bivalent (Figure 2C).

### Part II. Parameters of the BF Model and Their Roles for CO Patterns

BF simulations require specification of three types of parameters (Table 1). One set describes the nature of the precursor array upon which CO-designation acts; a second set describes features of the patterning process *per se*; and a third precursor specifies the efficiency with which a designated event matures into a detectable CO or CO-correlated signal.

**Table 1:** BF parameters.

Precursor parameters	
**N**	Average number of precursors per bivalent
**E**	Precursor distribution along chromosomes. 0 = random; 1 = even; 0< = E< = 1
**B**	Precursor distribution among chromosomes 0 = Poisson; 1 = constant; 0< = B< = 1
**s**	Distribution of precursor sensitivities to DDF (currently random)
**Density**	Precursor density can be varied along the chromosome as desired
**Patterning parameters**	
**S (Smax)**	Designation driving force; increased in simulations to maximum level; usually S>1
**L = L_BF_**	Distance over which interference signal spreads (total for both directions)
**A**	Reactivity of precursor to local value of (s); (A 1 = s^2^; A2 = s; A3 = 5/s; A4 = 1/s)
**c**	End effects on interference (cL and cR) 0 = unclamped; ≥1 = clamped
**Other parameters**	
**M**	Efficiency with which a CO-designated interaction matures to a detectable CO

#### The precursor array

The precursors for CO patterning are generally assumed to be the total array of double strand break (DSB)-initiated interactions between homologs. Several BF parameters describe the nature of this array. (N) specifies the average number of precursors per bivalent. (E) specifies the extent to which the precursors along a given bivalent are evenly or randomly spaced. (B) specifies the extent to which precursors occur at a constant value along a given bivalent in different meiotic nuclei or are randomly (Poisson) distributed among different nuclei. Also, different precursors will naturally exhibit a range of intrinsic sensitivities to the DDF. The parameter(s) specifies the distribution of those sensitivities as specified by the Matlab function “rand”.

**Figure 3:**
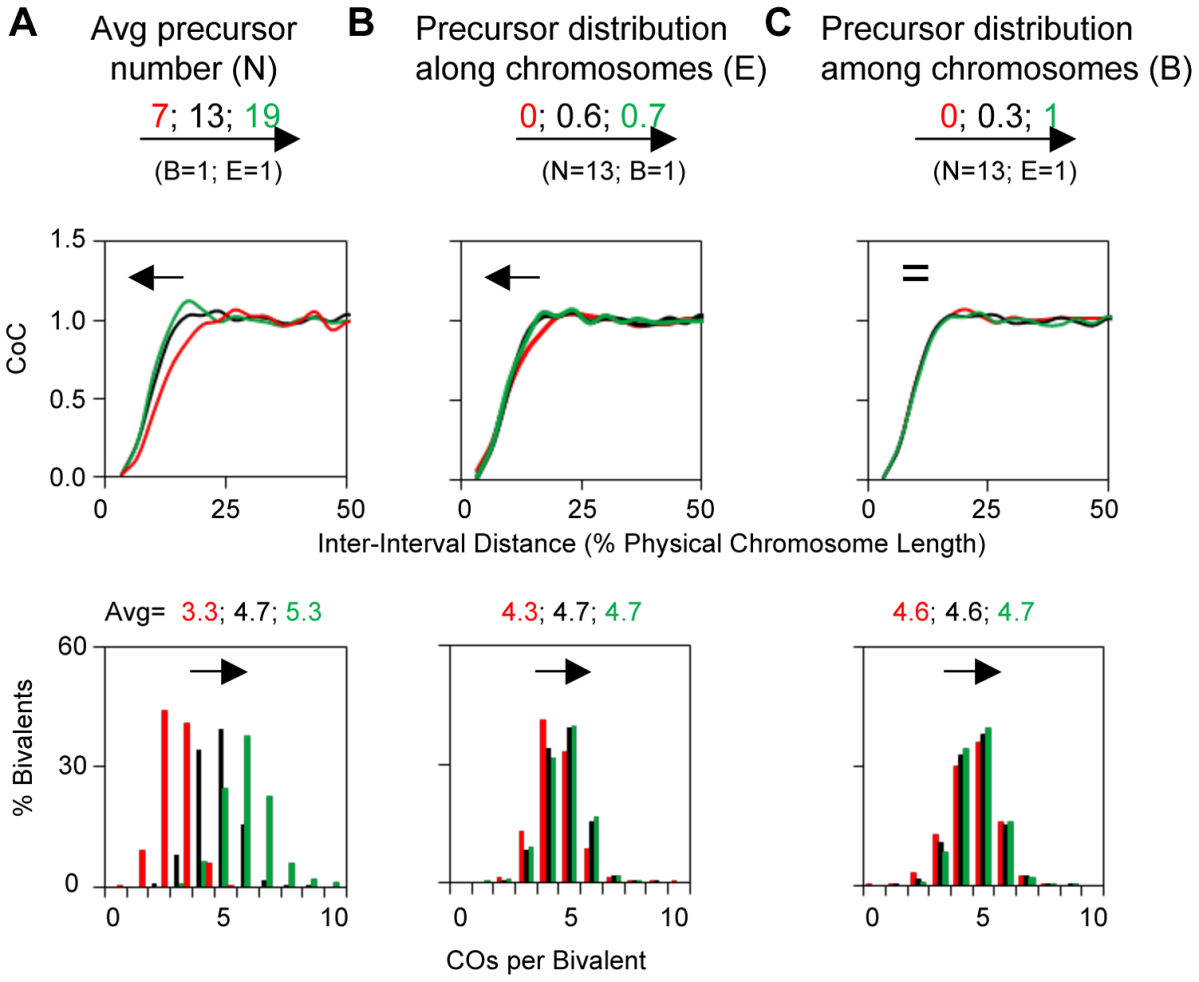
Variations in the precursor array can alter CO patterns. BF simulations were carried out under a set of “standard conditions” except for variations in the parameter of interest as indicated. Panels (A), (B) and (C) illustrate the effects of variations in N, E and B. Standard simulation parameter values are the same as the best-fit values for yeast Chromosome XV (Table 2). Parameter (E) is the standard deviation of the average inter-precursor distance and the corresponding evenness level is also shown by the shape (ν) of gamma distribution in the program output. For E = 0, 0.6 and 0.7, the corresponding ν = 1, 2.4 and 4.2, respectively. Parameter (B) is set by using the binomial distribution, in which with an average number of precursors on each bivalent (a constant mean), the distribution of the number of precursors among bivalents (probability of success for each trial) can be adjusted by changing the number of the trials.

The original BF model included (N) and (s) and assumed a given chromosome has the same number of precursors in different nuclei but assumed that precursors are distributed randomly along a given chromosome (B = 1, E = 0). The latter assumption is likely not the case *in vivo*. Experimental evidence in several organisms shows that precursors tend to be evenly spaced, sometimes dramatically (e.g. [10]–[14]). And anecdotal evidence further suggests that the number of precursors tends to be quite constant for a given bivalent in different nuclei (e.g. [10], [12], [15], [16]). We further note that evenly-spaced precursors have not been taken into account in any previous quantitative model for CO patterning (e.g. [17]–[20]). Variations in the nature of the precursor array can affect CoC relationships, with interactive effects, particularly at low values of (N) (Figure 3ABC; Figure S2).

The BF simulation program now also includes a feature which permits the density of precursors to be varied along the chromosome in a desired pattern. This feature is useful for modeling effects such as the paucity of DSBs in centromeric regions, or other regional and domainal variations in DSB levels along chromosomes (e.g. [5], [6], [10], [21]–[26]). Application to grasshopper CO patterns is described below.

The BF model assumes that precursors do not turn over, i.e. that a precursor either develops into a CO or into some other type of product, without being recycled to serve again as a precursor in another position. This assumption has not been directly tested. However, it seems reasonable because precursors are known to be highly evolved multi-protein complexes whose numbers can be constant over long periods of time (e.g. [12]).

The BF model also assumes that the entire precursor array is established prior to CO-designation (or essentially so). This is clearly true in some organisms (e.g. [12]). It is not so clear in other organisms, where different regions of the genome can be at significantly different stages within a single nucleus (e.g. [27]). However, BF simulations will still pertain in the latter case if the effects of CO-designation at earlier-evolved positions can be “stored” within the chromosomes and exert their effects upon nearby positions when the appropriate precursors do finally evolve.

Finally, it has sometimes been considered that CO patterns evolve in two stages (e.g. [11], [15]). In such a case, one round of event-designation is imposed on total DSB-mediated interactions, giving a set of intermediate designated sites. That intermediate set then undergoes a second round of designation. BF simulations can directly model this situation. DSB-mediated interactions are used as a first set of precursors for a first simulation to give the intermediate array of events. That intermediate array is then used as a second set of precursors for a second simulation. The predicted outcomes from one- and two-round scenarios for recombination-related markers in Sordaria meiosis are presented elsewhere. A useful feature in distinguishing between the two scenarios is whether closely-spaced COs ever do, or do not, occur at the specific spacing characteristic of the first precursor array. If COs arise in a single step, closely-spaced events can occur at the positions of adjacent precursors. If COs arise in two steps, then this will not occur; instead, closely-spaced COs can only occur at the spacing of adjacent precursors in the intermediate array (Figure S3).

#### CO patterning parameters: DDF (S), interference distance (L), precursor reactivity (A) and end effects (cL, cR)

All of these patterning parameters are present in the original BF model. Detailed explanations of their significance are as follows:

(S,Smax) versus (L): The outcome of the patterning process is determined primarily by two basic parameters: the strength of the (CO)-designation driving force (global stress or the DDF), as given by parameter (S); and the distance over which interference spreads, given by parameter (L).

For simulation purposes, the value of (S) is progressively increased to a specified maximum (Smax). A first, most reactive, precursor goes critical to give a CO-designation with accompanying interference; the level of (S) is then further increased, giving a next designation at the next most reactive position. This process is increased up to a final desired level. This procedure gives sequential CO-designations. The higher the final maximum value, (Smax), the more CO-designations. Interference arises instantaneously after each designation, reducing the probability that affected precursors can respond to the driving force over the specified distance (L).

**Figure 4:**
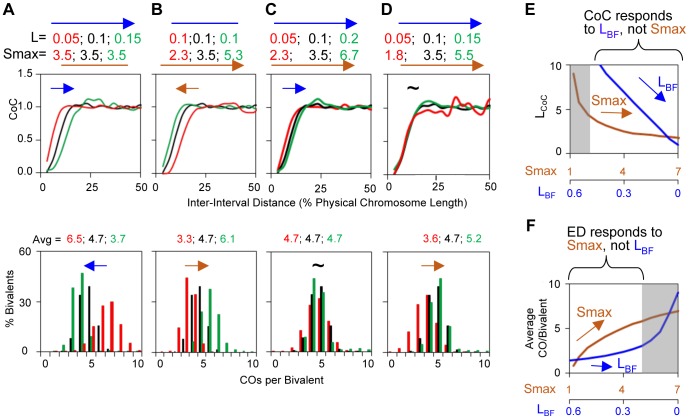
Variations in the L and/or Smax can alter CO patterns. All panels: BF simulations as in Figure 3. Panels (A) and (B) illustrate the effects of variations in L or Smax, respectively. Panels (C) and (D) illustrate the fact that very similar CoC and ED relationships can be achieved by appropriate different combinations of L and Smax, but with a differential response of CoC relationships to changes in L (C) and of ED relationships to changes in Smax (D). These differential responses are further documented in Panels (E) and (F).

The final overall pattern of COs reflects the balance between the CO-designation driving force (DDF) and the interference distance, i.e. the values of (Smax) and (L). Correspondingly, a change in the value of either parameter can confer a similar alteration in CoC relationships and the ED (Figure 4AB). Higher (Smax) or lower (L) permits more COs to occur at shorter inter-interval distances, thus shifting the CoC curve to the left. Concomitantly, the overall level of COs increases. Lower (Smax) or higher (L) has the opposite effects. The ED changes commensurately.

To a considerable extent, opposing variations in the two parameters can compensate for one another (Figure 4CD). Nonetheless, in most cases, the effects of variations in (L) and (Smax) can be distinguished. The primary target of (L) is inter-CO communication, with the number of COs affected as a secondary consequence. The primary target of the DDF (Smax) is the number of COs, not inter-CO communication, with inter-CO relationships affected as a secondary consequence. Correspondingly, variations in (L) primarily affect CoC relationships whereas variations in (Smax) primarily affect the ED (Figure 4CDEF). The practical implication for best-fit BF simulations is that the values of these two parameters can be specified independently (Figure S4).

We note that, *in vivo*, (S) and (Smax) could take a variety of forms. (i) The value of Smax could potentially be defined by the time available for CO-designation. Interestingly, in Drosophila, the presence of a structural chromosome heterozygosity (deletion or inversion) results in a delay in meiotic progression and an increase in the number of COs without loss of CO interference [28], [29]. This constellation of phenotypes could be modeled by an increase in Smax. (ii) CO designation could occur sequentially without any progressive increase in the DDF, simply because different precursor complexes will tend to undergo designations sooner or later in relation to their intrinsic reactivities, up to the maximum number specified by Smax.

Also: in the BF model, the strength of the interference signal decays exponentially with distance from the CO-designation site (Figure 1A) in accord with the way in which stress redistributes in the beam-film system upon which the mathematical expressions are based [3], [4] (Figure 1A legend). This decay relationship can be altered in the simulation program. However, we have found no need to do so thus far (e.g. below).

**Figure 5:**
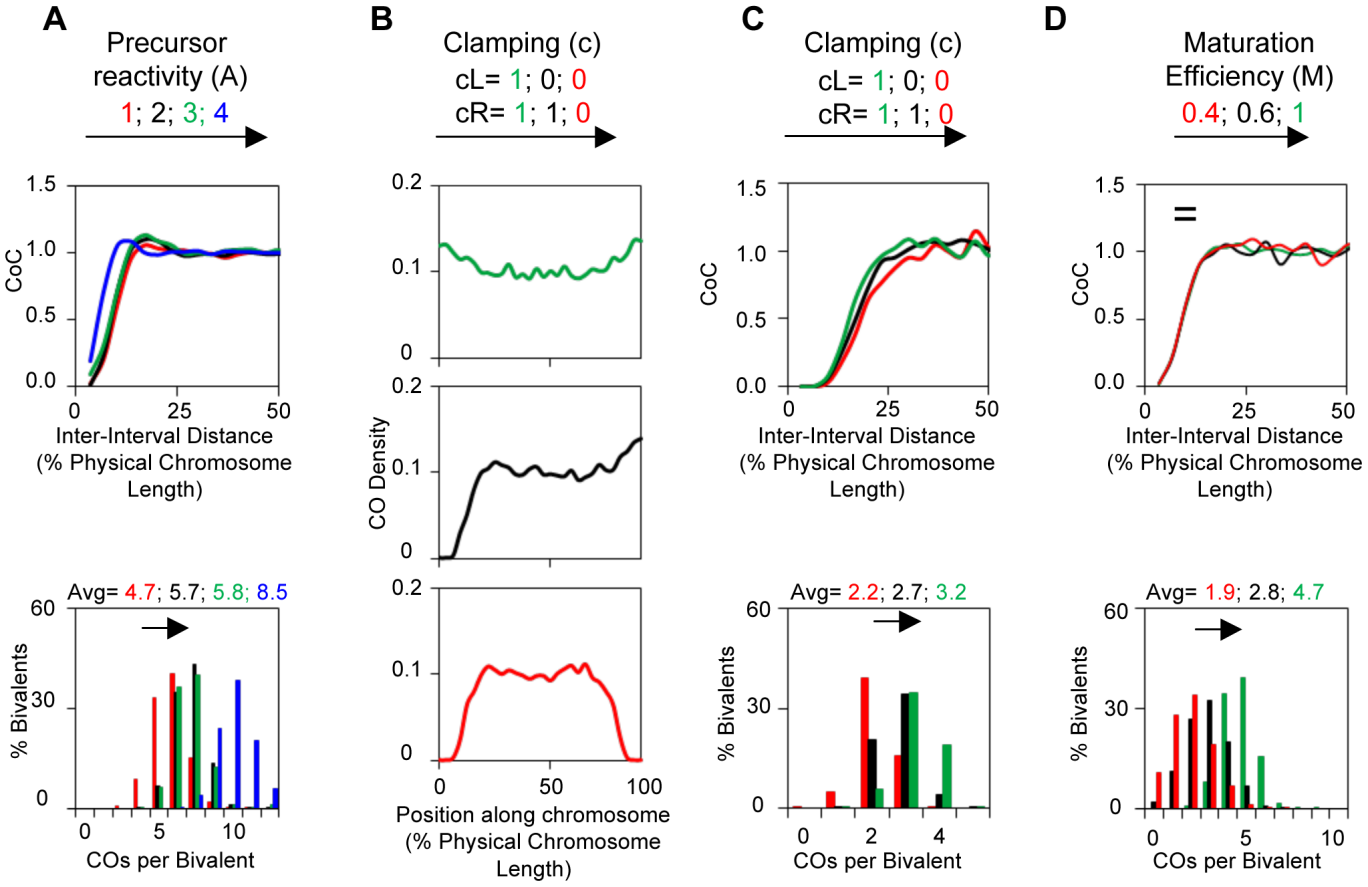
Variations in A, c or M can alter CO patterns. All panels: BF simulations as in Figure 3. (A) Effects of variations in precursor reactivity relationships. (B, C) Effects of variations in end clamping status on the distribution of COs along the chromosome (Panel B) and on CoC and ED relationships (Panel C). (D) Variations in maturation efficiency (M) do not affect CoC relationships but do affect the ED.

Finally, the value of (Smax) actually incorporates the combined effects of the driving force and the sensitivity of precursors to that force. Similarly, the value of (L) incorporates the combined effects of the strength of the interference signal (as it dissipates with distance) and the sensitivity of precursors to that signal. Put another way: any difference that can be modeled by a change in (Smax), or a change in (L) could, potentially, reflect a change either an actual change in the altered feature or a change in the ability of precursors to sense that feature. Other information must be brought to bear to distinguish the two types of effects.

(A): A third patterning parameter, (A), describes the dose/response relationship between precursor sensitivity (s) and the local stress/DDF level at the corresponding position (i.e. the value of s as modified by the effects of any interference signals that have emanated across that position). Parameter (A) can have one of four possible values. In two cases (A = 1, 2), reactivity varies directly with (s); in the other two cases (A = 3, 4), reactivity varies inversely with (s) (Table 1). Variations in (A) can affect CO patterns (Figure 5A).

Clamping: c(L) and c(R): Special considerations apply to interference at chromosome ends. These effects are incorporated into BF simulations by “clamping” parameters (cL and cR). In the absence of any other consideration, a terminal region will behave the same way as any other region of the chromosome with respect to its response to the DDF (Smax), the interference signal (L) and precursor reactivity (A). The interesting consequence of this effect, not regularly appreciated, is that there will automatically be intrinsic tendency for the ends to exhibit higher frequencies of COs, because these regions will be subjected to interference signals emanating in from only one direction (i.e. from internal regions of the chromosomes and not from regions “beyond” the end of the chromosome) (Figure 5B top). In a mechanical model, this “default” situation is achieved by “clamping” the end of the chromosome to some object. In BF simulations, clamping is defined by parameter (c), which can be specified individually at each chromosome end (cL and cR). The default case, fully clamped, is c = 1.

In a mechanical model, the chromosome end could alternatively be free in space, i.e. would be “unclamped”. Since such a free end cannot support stress, it would behave as if it already had experienced a CO, i.e. with an interference signal having spread inward. The result would be a decreased probability that COs will occur near that end. In this case, c = 0. Intermediate situations can also occur. Thus, c(L) and c(R) can take any value between 0 and 1.

As a practical matter, specification of (cL) and (cR) permits more accurate modeling of *in vivo* patterns where end effects are prominent. For example, many organisms exhibit a tendency for COs to occur near the ends of chromosomes whereas others do not. Such effects tend to emerge when chromosome ends are clamped (e.g. Figure 5B middle, bottom). [Notably, however, genetic variations that result in paucities or excesses of DSBs near ends (e.g. [6], [10], [21]–[26], [30]) should be modeled by variations in the precursor density (above) rather than as effects on interference].

Variations in (cL) and (cR) primarily affect the distribution of COs along the length of a bivalent but also have secondary effect on overall CoC and ED relationships (Figure 5BC).

#### Maturation efficiency (M)

A precursor that undergoes designation may not mature efficiently into the signal used to define designations experimentally. This situation, defined by the value of the parameter (M), occurs in diverse mutant situations. If maturation is less than 100% efficient, the initial array of CO-designated events undergoes random subtraction such that the final array of detected events reflects only a subset of the original designation array. Variations in (M) do not affect CO patterns. Since maturation efficiency only affects CO status after patterning is established, a decrease in (M) only shifts ED relationships to lower CO numbers, with no/little effect on CoC relationships (Figure 5D).

### Part III. BF Simulations Accurately Describe Experimental Data Sets

Application of the BF model to an experimental data set permits the identification of a set of parameter values for which simulated CO patterns most closely match those observed experimentally (general strategy described and illustrated in Figure S4).

Best-fit simulation analysis for data sets from yeast, Drosophila, tomato and grasshopper demonstrates that the logic and mathematics of the BF model can describe experimental CO patterns with a high degree of quantitative accuracy. This conclusion is evident in descriptions of CoC and ED patterns as described in this section (III). Additional evidence is provided by applications and extensions of BF simulation analysis to CO homeostasis, the obligatory CO and non-interfering COs as described in sections IV–VI. Inspection of experimental CoC relationships has also provided new information regarding the metric of CO interference in tomato and the fact that interference spreads across centromere regions (in grasshopper, as previously described, and also in tomato and yeast).

#### Budding yeast

**Figure 6:**
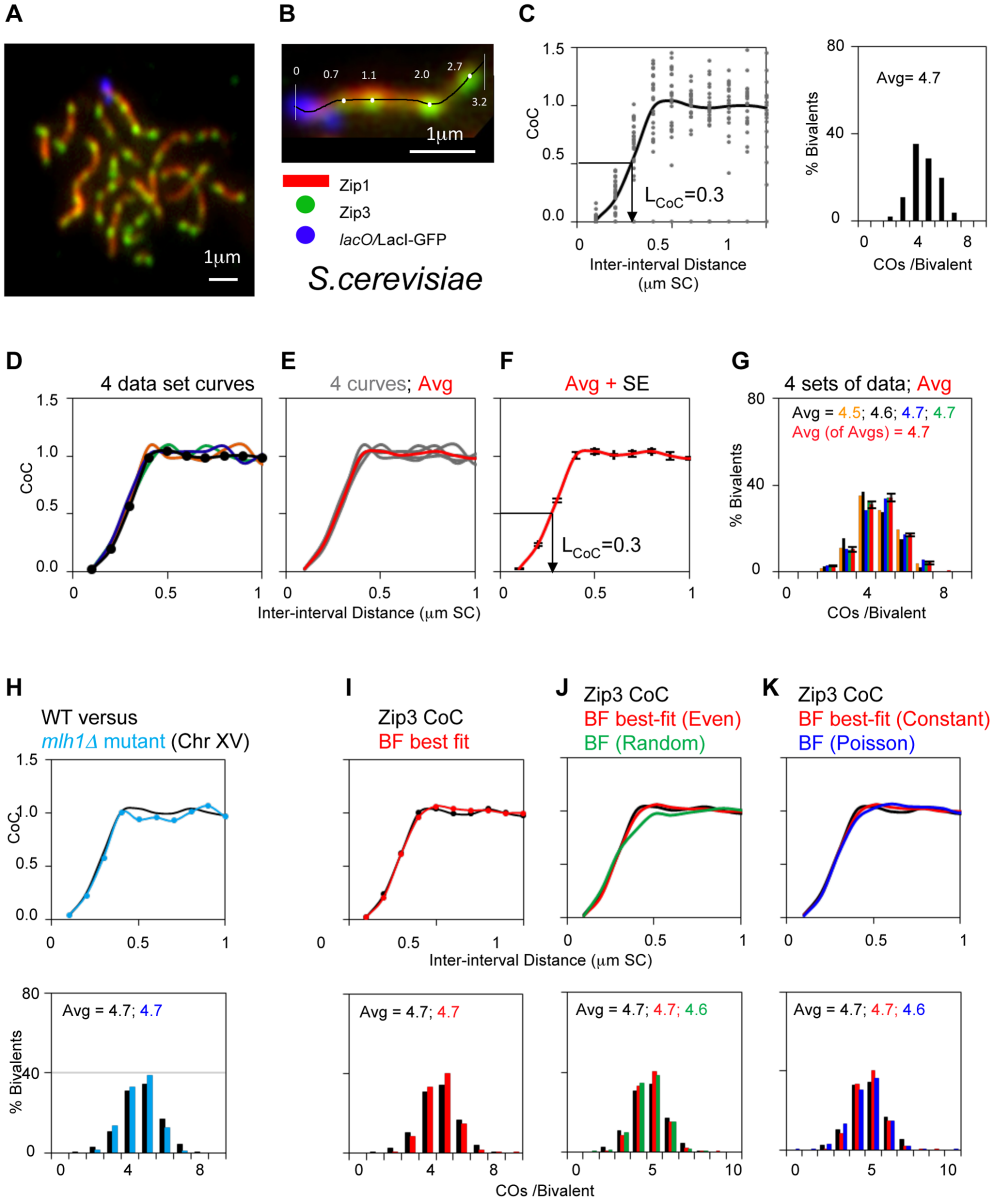
Experimental and BF analysis of CO patterns in budding yeast. Panels (A, B): Experimental System. (A) Spread yeast pachytene chromosomes fluorescently labeled for SC component Zip1, CO-correlated foci of ZMM protein Zip3, and terminally labeled at the end of Chromosome XV by a *lacO*/LacI-GFP array. (B) Positions of Zip3 foci along a single Chromosome XV bivalent were defined as shown. Panels (C–H): Experimental CO patterns for Chromosome XV. (C) CoC and ED relationships for a single representative Chromosome XV data set reflecting CO positions defined along >300 bivalents (as in (B)). Average CoC curve (black line) shows L_COC_ = 0.3 µm. (D–G) CoC curves and EDs for four independent experiments like that in (C). (D) shows the four individual average CoC curves; data set from panel (C) in black. (E) shows the four curves from four independent data sets and their average (in red). (F) shows the average of the four average CoC curves with the standard error at each inter-interval distance. (G) Shows the EDs for four independent experiments in (D–F) and the average (in red) with standard error. (H) Compares the average CoC curve and ED for an *mlh1*Δ mutant (blue) with those for WT (black; average of averages from panels (F) and (G)). Both ED relationships and CoC relationships in the mutant are as WT since Mlh1 acts very late (text). Panels (I–K): BF simulations of CO patterns for Chromosome XV (data from average of averages in panels (F) and (G). (I) Best-fit simulation (red) versus experimental data (black). Best-fit simulation specifies relatively even spacing of precursors (E = 0.6) and a constant number of precursors along the chromosome in all nuclei (B = 1). Other parameter values are in Table 1. (J, K). Experimental data and best-fit simulation data (black and red, from panel (I)) are compared with simulation using the same parameter values as the best-fit simulation except that precursors are either randomly spaced (Panel J; E = 0; green) or Poisson distributed among chromosomes in different nuclei (Panel K; B = 0; blue). Best-fit simulations for Chromosome IV and III data and for chromosome XIV in BR are shown in Figure S5 A–D; parameter values in Table 1. Importantly, even spacing is important for the best fit in all cases. In contrast, constant and Poisson distributions give very similar matches to experimental data except for the case of Chromosome III, where constant distribution must be required to ensure a sufficiently low number of zero-CO chromosomes (Figure S5 EF).

Yeast provides a favorable system for analysis of CO patterning in general, and for application and evaluations of BF modeling in particular, for several reasons. First, in this organism, the sites of patterned (“interfering”) COs are marked by foci of ZMM proteins Zip2 or Zip3 along pachytene chromosomes ([31]; Materials and Methods; Figure 6A). Zip2/3 foci mark CO sites very soon after they are designated (and independent of the two immediate downstream consequences of CO-designation, i.e. formation of the first known CO-specific DNA intermediate and nucleation of SC formation). Thus, effects of CO maturation defects on CO patterns are minimized. Second, the positions of these foci, and thus of CO-designations, can be determined along any specifically-marked chromosome to the resolution of fluorescence microscopy (e.g. Figure 6B). Also, Zip2/3 focus positions can be determined even in the absence of SC [31]. Third, the average number of Zip3 foci (COs) per bivalent varies over a significant range (e.g. Table 2). The lowest value described thus far, ∼2 for Chromosome III, is close to the number of COs seen in some other organisms (e.g. mouse or human or grasshopper) and thus provide appropriate models for such cases. At the highest values, ∼7, multiple foci (COs) occur quite evenly along the length of the chromosome, which is very useful for revealing general patterns. Fourth, analysis of chromosomes in hundreds of nuclei is readily achievable, thus readily providing sufficiently large data sets for both wild-type and diverse mutant cases. Correspondingly, CoC and ED relationships can be determined extremely accurately. For a given data set, CoC values at each inter-interval distance can vary over a significant range (Figure 6C). This variation is largely due to sampling variation because it is significantly reduced in BF simulation data sets that involve 5000 chromosomes rather than the 300 that usually comprises a typical data set. Despite this variation, which is present in all experimental data sets (below), the average CoC curve obtained from such an experiment is highly reproducible, as illustrated by the results of four independent experiments (Figure 6D–G).

**Table 2:** Experimental characteristics and best-fit simulation parameters.

		Experimental data				Best-fit simulation values									
Organisms\Chromosomes		Chrom. length#		CO/Biv	% Zero-CO Biv	L_CoC_	L_BF_		Smax	A	cL/cR	N@	E	B	M
		Mbp	µm				Fraction	µm							
*S.cerevisiae*	Chr III	0.32	1.2	1.8	1	0.3	0.25	0.3	3.5	1	0.85/0.85	6	0.6	1	1
	Chr XV	1.05	3.2	4.7	0	0.3	0.1	0.3	3.5	1	0.85/0.85	13	0.6	1	1
	Chr IV	1.53	4.8	6.8	0	0.3	0.075	0.3	3.5	1	0.85/0.85	19	0.6	1	1
	Chr XIV (BR)	0.79	2.1	3.7	0	0.3	0.15	0.3	3.5	1	0.85/0.85	11	0.6	1	1
*Chorthippus*	L3	1800	94	2.2	0	28	0.3	28	2.3	2	1.1/0.96	14	0	1	1
*D.Melanogaster*	X	22	15	1.4	5	6	0.2	6	2.8	1	0.65/0.5	7	0.4	0.5	1
*S. lycopersicum*	Chr 2–4	70	22	1.4	0	11	0.65	14	1.05	3	1.7/1.1	25	0.6	1	1
		70	17	1.2	0	11	0.8	14	1.05	3	1.7/1.1	20	0.6	1	1

# The genomic chromosome length and SC length are from the following sources.

(1) *S.cerevisiae*: Saccharomyces Genomic Database (http://www.yeastgenome.org) and SC length are from this study and [31]. (2) *Chorthippus*: its C value = ∼10 pg (http://www.genomesize.com/results.php?page=1), and 1 pg = 978 Mb [70]; thus its genome size is ∼9780 Mb, chromosome L3 SC = 94 µm (0.189 of total SC length [71]), thus the predicted L3 is ∼1800 Mb assuming the SC length is proportional to genome size in this organism. (3) *D. Melanogaster*: Fly Database (http://flybase.org/). SC length is based on [72], [73]. (4) *S. lycopersicum*: Sol Genomics Network (http://solgenomics.net). SC length is from [38].

@The number of precursors is based on the following. (1) *S. cerevisiae*: Spo11 oligos and also microarray data [21], [52]. (2) *Chorthippus*: from [3] (3) *D. Melanogaster*: based on [3], [34], [35] (4) *S. lycopersicum*: based on [38].

Experimental CO patterns in wild-type SK1: We have defined Zip3 focus patterns along WT chromosomes III, IV and XV in the SK1 strain background. These chromosomes range in size from 330 to 1530 kb (Table 2). For CoC analysis, each chromosome was divided into 100 nm interval, a size that provides maximal accuracy (Figure S1). Inter-interval distances for CoC analysis are expressed in units of µm SC length (rationale above). The three analyzed chromosomes exhibit virtually overlapping CoC curves, with L_COC_ = ∼0.3 µm (Figure 6F; Table 2). Along a pachytene chromosome of SK1, this distance corresponds to ∼100 kb. The CoC curve remains less than 1 up to inter-interval distances corresponding to ∼150 kb, in accord with the maximal distance over which interference is detected by genetic analyses (e.g. [32]).

The value of Zip3 foci as a marker for CO patterns is further confirmed by analysis of an *mlh1*Δ mutant. Since Zip3 foci mark CO sites shortly after they are designated, and long before the late step at which Mlh1 is thought to act (above), the *mlh1*Δ mutation should not reduce total Zip3 focus levels and should have no effect on CoC relationships for Zip3 foci. This expectation is confirmed (Figure 6H).

BF analysis: CoC and ED data for all three analyzed chromosomes can be very closely matched by BF best-fit simulations (e.g. Figure 6I; best-fit simulations for SK1 Chromosome IV and III and for chromosome XIV in BR are shown in Figure S5 A–D; parameter values in Table 2). Points of note include:

1. Despite differences in absolute chromosome length, and numbers of Zip3 foci, all three chromosomes are described by the same set of optimal parameter values with the exception of the predicted number of precursors, which increases with chromosome length as could be expected (Table 2).
2. The thus-defined best-fit value of (L), i.e. the distance over which the inhibitory interference signal spreads, is ∼300 nm (L_BF_ = ∼0.3 µm) for all analyzed chromosomes (Table 2). This value of (L_BF_) turns out to correspond closely to “interference distance” as defined experimentally by CoC analysis in all cases (L_COC_ = ∼0.3 µm; Table 2).
3. For each chromosome, the number of precursors used for the best-fit simulation corresponds well to that described experimentally by analysis of DSBs and is approximately proportional to chromosome length [21].
4. An optimal match between best-fit CoC curves and experimental data requires that precursors be relatively evenly spaced along the chromosomes (E = 0.6; (Figure 6IJ; Figure S5A–E), thus confirming and extending experimental evidence that yeast DSBs are evenly spaced (discussion in [13], [14]; see also below).
5. Notably, also, for the shortest chromosome (III), an optimal match is obtained only if precursors are also assumed to occur in a relatively constant number along a given chromosome in different nuclei (B = 1; Figure S5F). At lower values of B, the frequency of zero-Zip3 focus chromosomes is higher than that observed experimentally because a significant fraction of chromosomes fail to acquire enough precursors to give at least one focus. This finding suggests that a given chromosome usually acquires the same (or nearly the same) number of precursors in every meiotic nucleus. For longer chromosomes, the value of B is not very important (e.g. Figure 6K; further discussion below).
6. Zip3 focus analysis reveals that the shortest yeast chromosome (III) has a significant number of zero-event chromosomes (1%) whereas the two longer chromosomes have much lower numbers (no zero-focus chromosome has been detected among >1000 chromosomes analyzed) (e.g. Figure 6G). These values are recapitulated by BF simulations (Figure 6I; Figure S5EF; further discussion below).

The ability of BF simulations to accurately describe yeast data is further supported by analysis of CO homeostasis and the “obligatory CO” as described below.

#### *Drosophila melanogaster*

**Figure 7:**
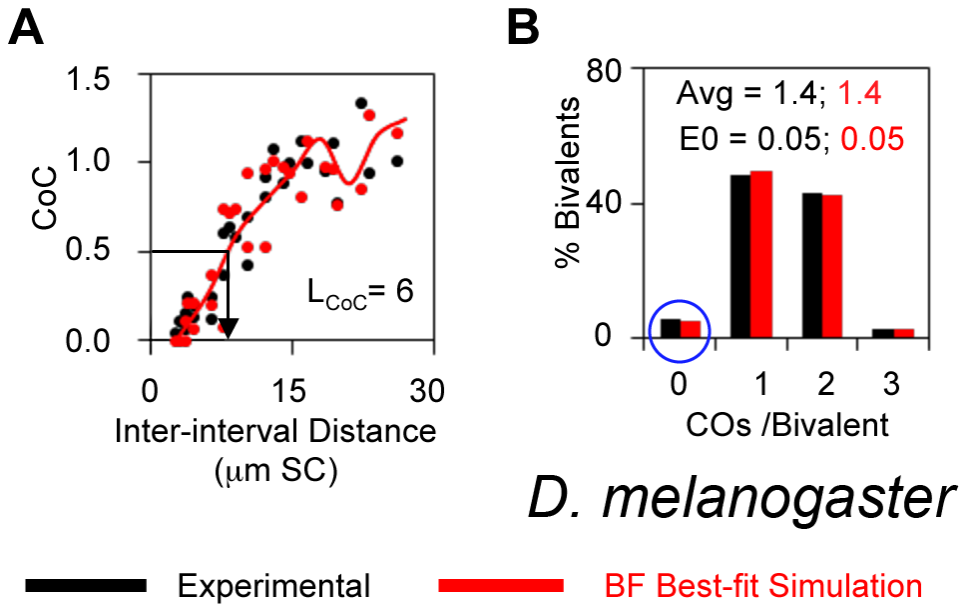
Experimental and BF analysis of CO patterns in Drosophila. (A, B). Experimental data (black) and BF simulations (red) for the *D. melanogaster* X chromosome.

CO interference was first discovered, and CoC analysis developed, by genetic analysis of Drosophila X chromosome (Introduction). These classical genetic data do provide for a quantitatively accurate CoC curve with L_COC_ = ∼6 µm (Figure 7A), because the interval sizes are small enough and the data set is large enough [33]. The average number of COs per bivalent defined by experimental analysis is 1.44 with an unusually high level of zero-CO bivalents (5%) (Figure 7B). Recent studies of γ-His2Av foci have defined a total of ∼24 DSBs for the entire genome in Drosophila female meiosis [34]. Assuming that DSBs are proportional to genome length, this implies 6 DSBs for the X-chromosome (N = 6). A BF simulation with N = 6 can quantitatively match the classical Drosophila X chromosome data for all descriptors (Figure 7AB). Best-fit simulation requires differential clamping at the two ends, with less clamping at the centromeric end (Table 2). This feature provides for the experimentally-observed tendency for CO distributions to be shifted away from that end (illustrated in [35]).

The ability of BF simulations to accurately describe Drosophila data is further supported by analysis of CO homeostasis and the “obligatory CO” as described below.

#### *Chorthippus bruneus* (grasshopper)

**Figure 8:**
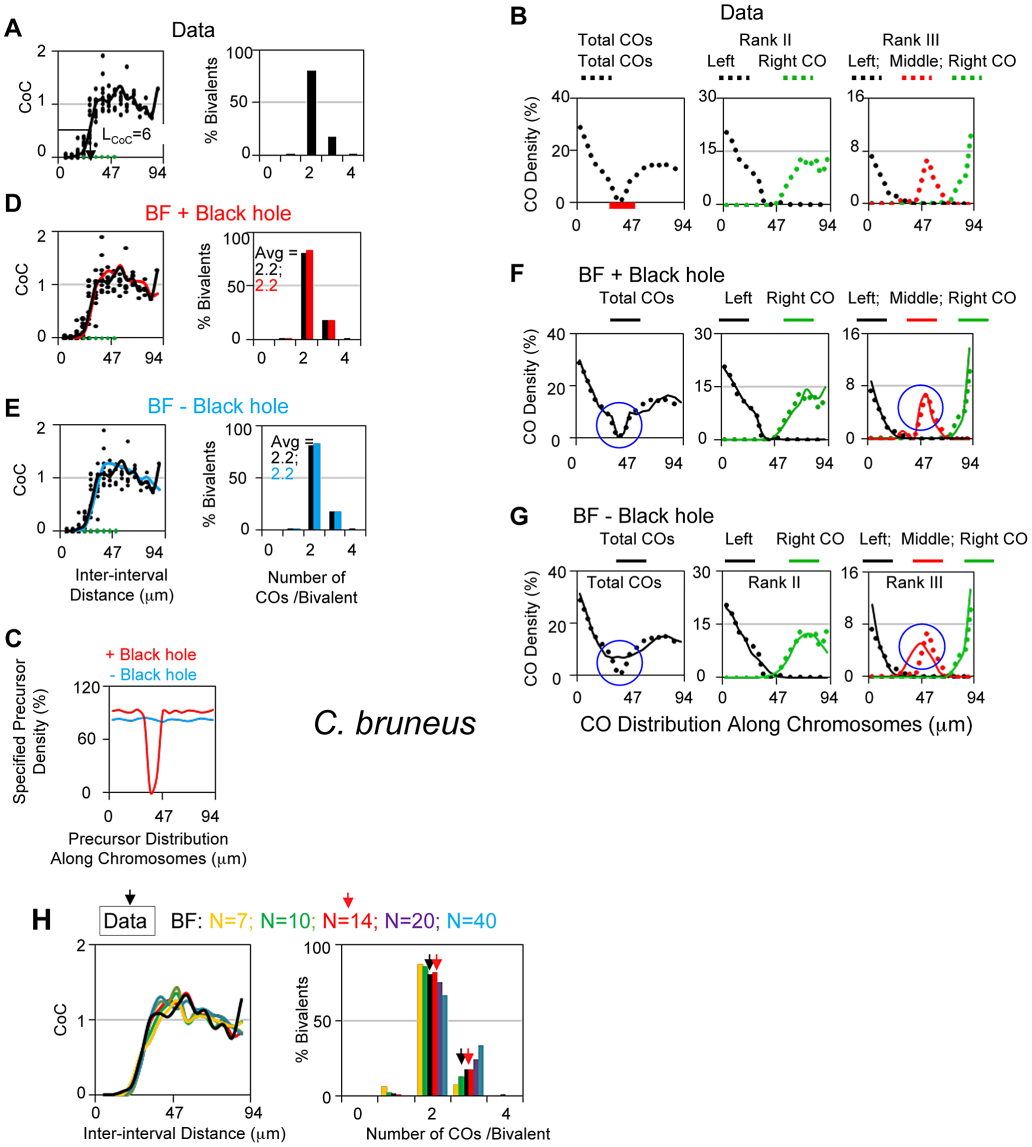
Experimental and BF analysis of CO patterns in grasshopper (*Chorthippus bruneus*). (A, B) Experimental data. (A) CoC and ED relationships; (B) Distribution of COs along the bivalent for total COs (left) and for bivalents with either two or three COs with different colors for first, second (and third) COs from the left end of the bivalent. The centromeric region is labeled by a red bar. (C–H) BF simulation analyses. (C) Precursor density (frequency of precursors pre bivalent per interval specified for simulations, where the number of intervals = 17 as for CoC analysis) for BF simulations that used either an even distribution along the chromosome (blue; − Black hole) or a distribution where precursor levels decrease to zero over a region corresponding the paucity of COs in the centromeric region (red; + Black hole; centromeric region defined in Panel B). (D) BF simulations with best-fit parameter values (Table 2) using the two precursor distributions defined in (C), i.e. with or without the centromere region black hole. CoC and ED relationships are the same in both cases (D and E); the distribution of COs along the chromosomes are well-fit when the black hole is included (F) as compared to when it is not (G). (H) BF simulations were used to estimate the likely value of N. CoC relationships seen experimentally (black) were compared by those given by BF simulations that use all best-fit parameter values except that the value of N (precursors per bivalent), which was varied from N = 7 to N = 40 (colors). Left panel: CoC relationships match the experimental curve for any N≥14 (i.e. all curves except gold and green which are N = 7 and N = 10). Right panel: ED relationships are best fit by N = 14 (compare red and black), with less good fits at lower and higher values (right side).

CO sites along the L3 bivalent of grasshopper have been defined by analysis of chiasmata in 1466 diplotene nuclei [36], [37]. These data yield a CoC curve with L_CoC_ = 28 µm and an ED with an average of 2.2 COs/bivalent and no detected zero-CO chromosomes (<0.07%; Figure 8A).

A prominent feature of the L3 bivalent is a severe paucity of COs in the centromere region (Figure 8B left panel). This feature presumably reflects a defect in occurrence of precursors (DSBs) in centromeric heterochromatin. Thus, for BF best-fit simulations, the precursor array was adjusted accordingly, to give a strong paucity of precursors in the centromere region (a “black hole”; Figure 8B left; C).

BF best-fit simulations can accurately describe L3 CoC and ED relationships, with or without inclusion of the black hole (Figure 8D, E); however, inclusion of the centromere precursor defect dramatically improves CO distributions along the chromosome, not only for total COs but for bivalents with two or three COs (Figure 8F versus 8G). Unlike all of the other cases analyzed above, there is no information for grasshopper regarding the number of precursors per bivalent. In BF simulations, the best fit between experimental and simulated data sets is provided when N = ∼14 (Figure 8H).

#### *Solanum lycopersicum* (tomato)

**Figure 9:**
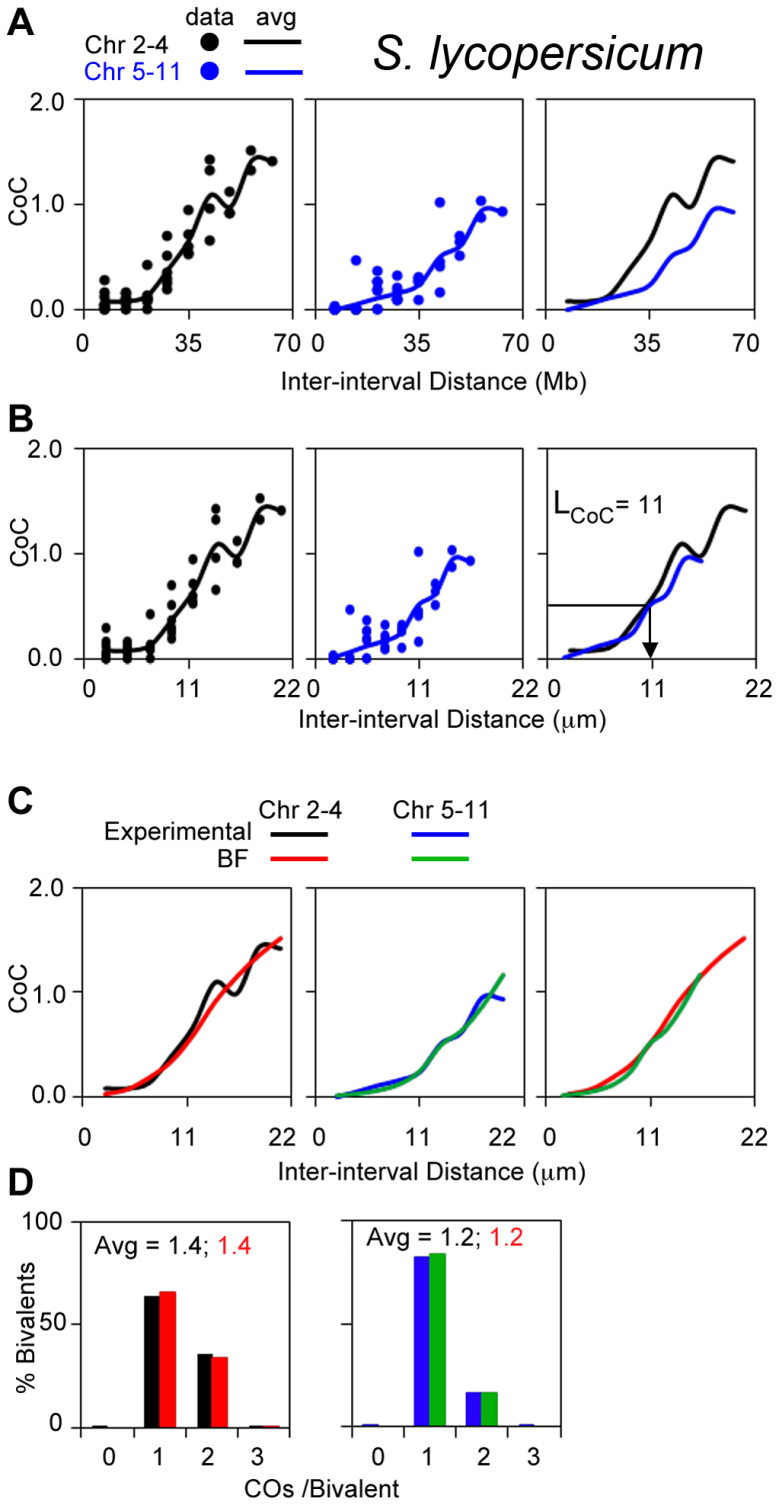
Experimental and BF analysis of CO patterns in tomato. (A, B) Experimental CoC data for chromosomes with shorter or longer SC lengths (2–4 and 5–11, respectively). Curves for the two groups are offset when inter-interval distance is expressed in units of Mb (genomic distance) (Panel A) and are overlapping when distance is expressed in units of µm SC (physical distance) (Panel B). (C, D) BF best-fit simulations for both groups of chromosomes using the physical distance metric (µm SC length) and adjusting precursor number as required to give ∼20 precursors per CO for each type of chromosome. This relationship is based on observation of ∼280 Rad51 foci [69] and 15 Mlh1 foci genome wide [38]. Interestingly, this relationship further implies that N is proportional to SC length.

CO patterns in tomato have been defined by analysis of Mlh1 foci ([38]; Lhuissier F.G. personal communication). Chromosomes in this organism exhibit a range of different pachytene bivalent lengths [38]. Bivalents comprise two groups, chromosomes 2–4 and 5–11, on the basis of longer and shorter SC lengths respectively (Figure S6A). We find that experimental CoC curves are significantly offset for the two groups when the metric of inter-interval distance is Mb (Figure 9A); in contrast, CoC curves for the two groups are superimposable when inter-interval distance is µm SC length (physical distance), with L_CoC_ = 11 µm (Figure 9B).

Physical distance has shown to be the appropriate metric for CO interference in mouse and Arabidopsis (above). The experimental results for tomato described above (Figure 9 AB) imply that this is also true in tomato. In addition, these data imply that the ratio of Mb to µm SC length is higher for shorter chromosomes than for longer chromosomes. This difference is explained, quantitatively, by two facts that: (i) heterochromatic DNA is much more densely packed along the SC than euchromatin and (ii) shorter chromosomes have a higher proportion of heterochromatin than euchromatin (and thus shorter SC lengths) (Figure S6).

CoC and ED patterns for both groups of chromosomes are well-described by BF simulations (Figure 9CD). All parameters have the same values in both cases, including the interference distance (L) when expressed in µm SC length, except that precursor number varies with CO number/SC length (Figure 9CD legend).

#### Interference spans centromeres in grasshopper, yeast and tomato

**Figure 10:**
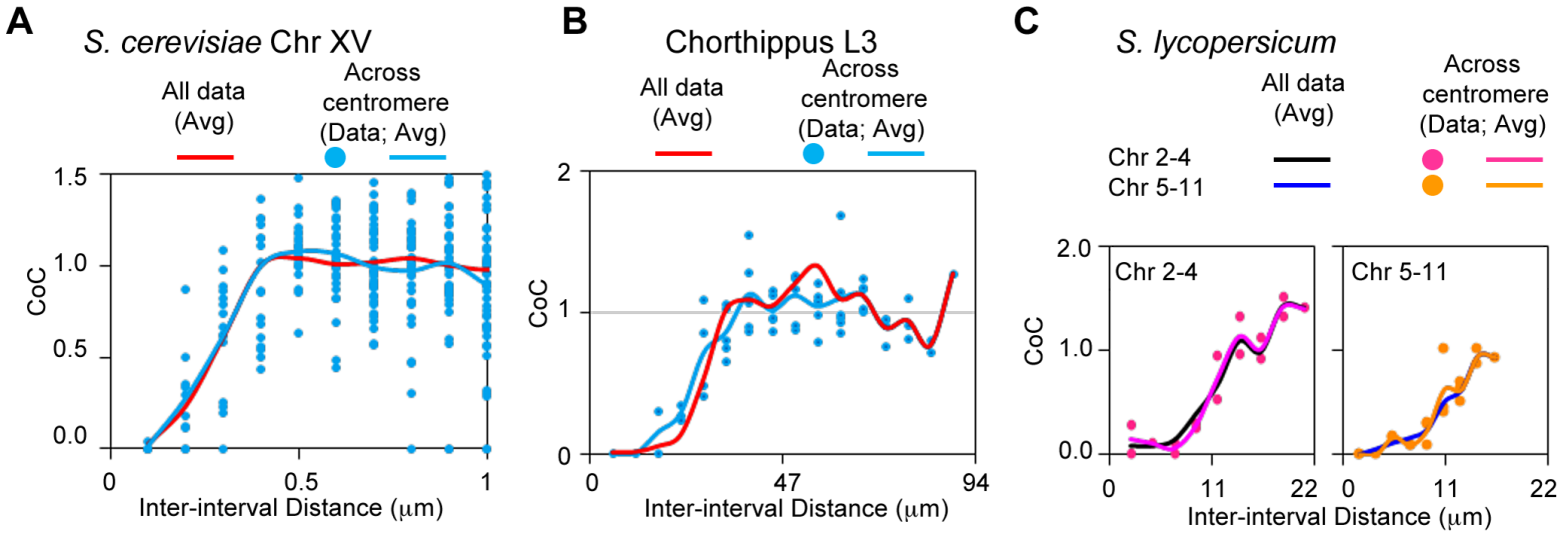
Interference spreads across centromeres in yeast, grasshopper and tomato. CoC values for interval pairs that span centromere regions, and the corresponding average CoC curves, closely match the average CoC curves for all interval pairs for yeast (Panel A), grasshopper (Panel B) and tomato, both bivalent groups as described in Figure 9B (Panel C).

Previous analyses of interference have shown that CO interference is transmitted across centromeric regions [6], [37]–[42]. Correspondingly, CoC values for interval pairs that span centromeres are almost indistinguishable from those for pairs separated by the same distances that do not span centromeres (Figure 10B). This is remarkable given the manifestly different structure in these regions. We further find that the same is true for budding yeast, based on Zip3 focus analysis (Figure 10A) and for tomato, from Mlh1 focus analysis (Figure 10C).

### Part IV. CO Homeostasis

Experimental evidence has revealed that variations in the level of recombination-initiating double-strand breaks (DSBs) are not accompanied by corresponding variations in the number of COs. When DSB levels are either reduced or increased, CO levels are not reduced or increased commensurately [15], [34], [43]–[46]. This phenomenon is referred to as CO homeostasis [43].

**Figure 11:**
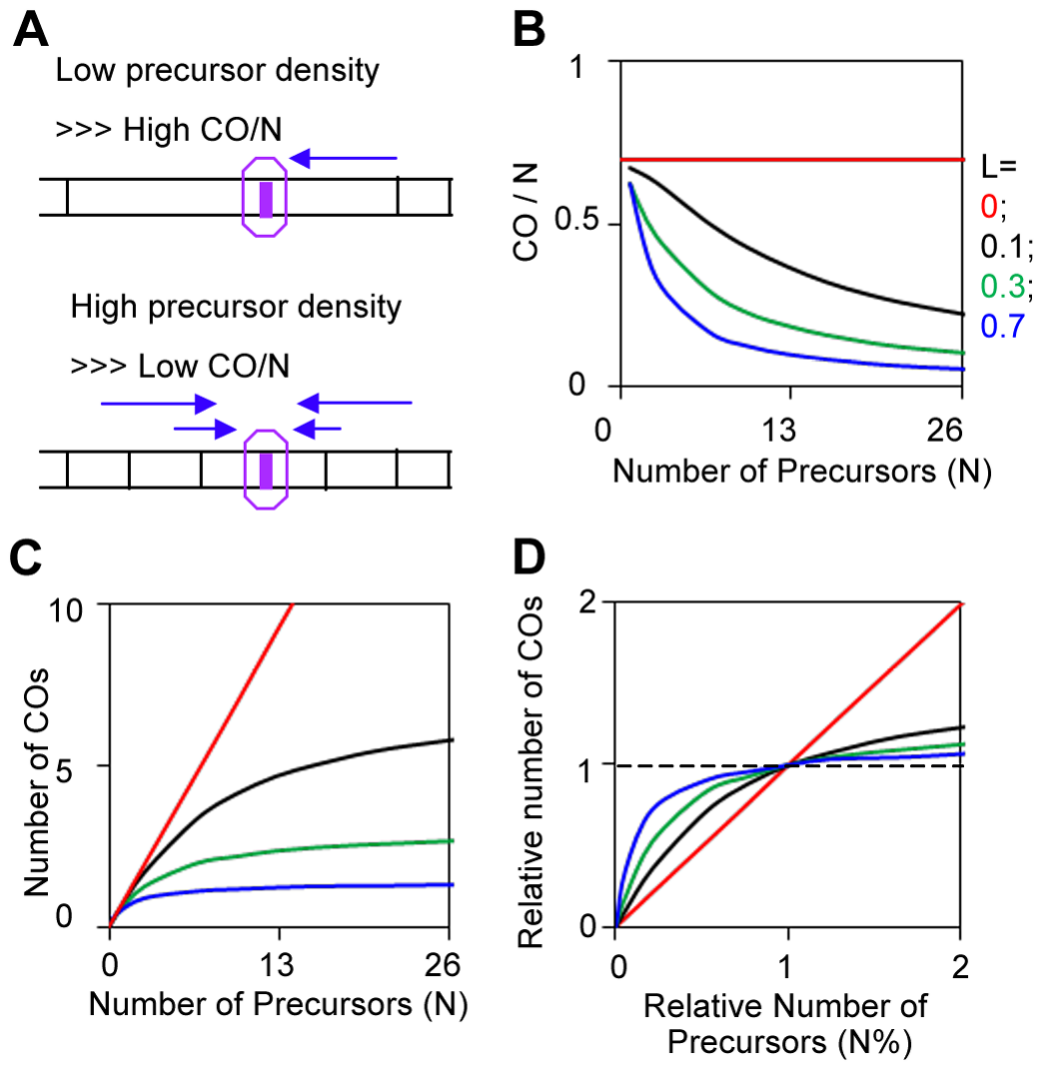
CO homeostasis and quantification by BF simulations. (A) CO homeostasis is the phenomenon that, as the level of DSBs (precursors) increases or decreases, there is a less than proportional change in the frequency of COs. The basis for this effect is illustrated. At lower (higher) precursor density, a given precursor will be less (more) likely to be subject to interference and thus more (less) likely to give a CO. These relationships further imply that the extent of CO homeostasis at a given precursor density will also vary with interference distance and the strength of the DDF (L and Smax) and other patterning features as reflected in L_CoC_ (text). (B, C, D) CO homeostasis was modeled by BF simulations using standard parameter values (Figure 3) with the indicated variations in the number of precursors (N), and varying values for the interference distance (L). CO homeostasis can be viewed as a function of N and L, as an effect on the probability that a single precursor will become a CO (B); or as an effect on the total number of COs along a chromosome (C). Also, the values of N and of COs can be defined as 1 for the wild-type reference situation and the effects of varying N (as a percentage of the reference value) can be seen directly as effects on CO levels (also as a percentage of the reference value). This approach considers the “densities” of precursors and COs rather than the absolute levels.

According to the BF model, CO homeostasis is dependent upon, and in fact is a direct consequence of, CO interference (Figure 11A), as proposed [43], [46]. In the absence of interference, the probability that a precursor will give rise to a CO is a function only of its own intrinsic properties, independent of the presence/absence of other precursors nearby. Thus, as the number of precursors decreases, the number of COs will decrease proportionately. In contrast, if interference is present, each individual precursor is subject to interference that emanates across its position from CO-designation events at neighboring positions. The lower the number of precursors, the less this effect will be. Thus, assuming a fixed level of CO interference, the frequency of COs per precursor will increase as the number of precursors decrease. Put another way: as the density of precursors decreases, the ratio of COs to precursors increases, even though there is no change in CO interference. Importantly, since CO homeostasis requires CO interference, its magnitude will also depend on the strength of CO interference as discussed below.

#### In the BF model, CO homeostasis involves interplay between N and patterning parameters, e.g. L and Smax

CO homeostasis for a given condition can be defined quantitatively by BF simulations in which the value of N (which is the number of precursors per bivalent and thus corresponds to precursor density) is varied, with all other parameter values remaining constant. Homeostasis can be described by plotting, as function of (N), either the frequency of COs per precursor (CO/N) or the total number of COs per bivalent (which corresponds to CO density) (Figure 11BC). Alternatively, CO homeostasis can be seen from the perspective of a starting wild-type situation with the number of COs per bivalent at the wild type precursor level taken as the point of reference and variations in the number of COs and precursors expressed relative to those reference values (Figure 11D).

The magnitude of CO homeostasis, i.e. the extent to which CO levels fail to respond to changes in precursor levels, will vary with the level of CO interference (above). This relationship can be described quantitatively by carrying out simulations for different values of (N) at different values of patterning parameter(s), illustrated here for variations in the interference distance (L) (Figures 11B–D). In the absence of interference, CO levels vary directly with precursor levels; the greater the interference distance, the less the change in CO levels with precursor levels. Notably, at very long interference distances, CO levels do not change at all with precursor levels. This is basically because interference precludes CO-designation at all other precursor sites, thus rendering variations in CO density irrelevant. This latter situation has recently been documented for *C.elegans* [44], [45]. Analogous effects can be seen for variations in Smax (not shown), in accord with the fact that both L and Smax play important roles for L_CoC_ (above).

#### BF simulations of CO homeostasis in experimental data sets can be used to evaluate the validity of the BF model

BF best-fit simulations for wild type experimental data sets provide specific predicted values of all BF parameters, including N, L and Smax (above). Given this starting point, BF simulations can then specifically predict how CO levels will vary if the level of DSBs (precursors) is decreased or increased. If the BF model accurately describes CO patterning, the best-fit simulation will accurately describe the experimental data. Application of this approach to budding yeast and Drosophila shows that the BF model can quantitatively predict experimental CO homeostasis patterns in both organisms.

**Figure 12:**
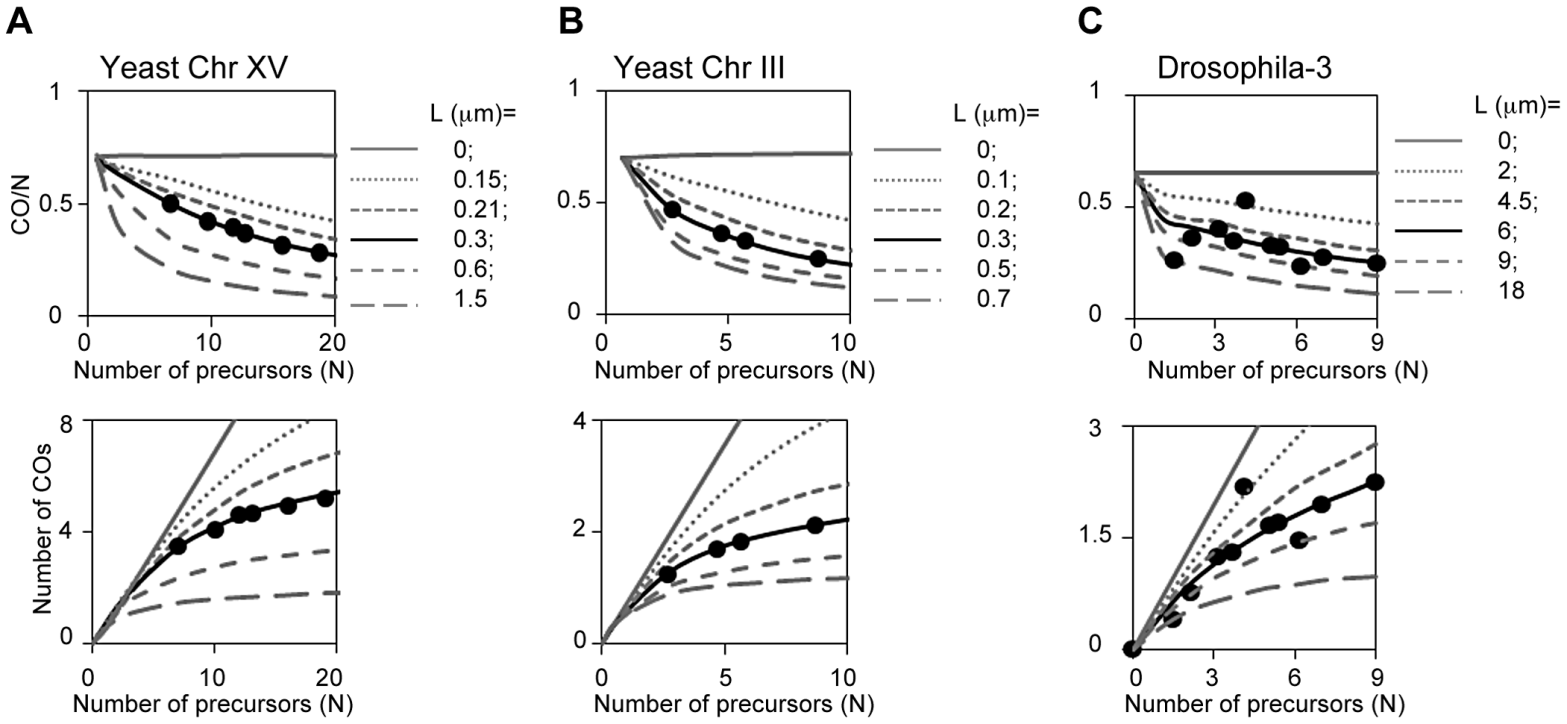
CO homeostasis in yeast, Drosophila, tomato and grasshopper. (A, B) CO homeostasis relationships were determined experimentally for yeast chromosomes XV and III (text; Figure S7); plotted (filled circles); and compared with the values predicted from BF simulations based on best-fit parameter values for the two chromosomes (L = 0.3 µm, black lines). Predictions for other values of L are shown for comparison (grey lines). Experimental data precisely match BF simulation predictions. (C) CO homeostasis relationships determined experimentally for Drosophila chromosome 3 were compared with the predictions of the corresponding BF simulation (L = 6 µm, black line). Predictions for other values of L are shown for comparison (grey lines). Experimental data precisely match BF simulation predictions. [Note: Drosophila analysis was carried out as follows. Variation in CO number as a function of DSB level was determined experimentally for a fragment of chromosome 3 [34]. We first defined the theoretical CO homeostasis curve (black line) for full length chromosome 3 by BF simulations using the same set of parameters defined for chromosome X except that the number of precursors was adjusted in proportion to relative chromosome length (chromosome 3 is 1.5× the length of chromosome X). The CO levels observed experimentally on the chromosome 3 fragment at the different DSB levels were then adjusted to those expected for the full length chromosome under the assumption that CO frequency is proportional to chromosome length, as in all other cases (above). The resulting experimental values were then compared with the theoretical curve and its relatives constructed at varying values of L (grey lines).]

The BF model quantitatively describes CO homeostasis in yeast: We asked whether the BF model could quantitatively explain CO homeostasis along chromosomes XV and III as defined by Zip3 focus analysis (above). We determined experimentally the number of Zip3 foci that occur along the two test chromosomes in a series of mutants that are known to exhibit particular, defined decreases or increases in DSB levels. Reductions in DSB levels were provided by the hypomorphic alleles of DSB transesterase Spo11 used to originally define CO homeostasis [43]. An increase in DSB levels was provided by a *tel1D* mutation, which increases DSB levels without significantly altering CO interference ([13]; unpublished). The observed experimental relationships are described by the BF-predicted relationships (Figures 12AB, L = 0.3 µm).

The robustness of BF simulations is further supported by analysis of CoC and ED relationships for Zip3 foci in each of the DSB mutant strains. Best-fit simulations of these data sets should occur at exactly the same values of all parameters except for the number of precursors (N), which should match that defined by experimental analysis of DSBs. Both of these predictions are fulfilled (Figure S7). These comparisons also reveal some interesting subtleties to DSB formation in the mutants (Figure S7).

The BF model quantitatively explains CO homeostasis in Drosophila: We analogously evaluated whether BF simulations accurately predict CO homeostasis relationships in Drosophila, which were defined experimentally by analysis of a fragment of chromosome 3 [34]. In Drosophila, as in yeast, CO-versus-DSB experimental data exhibit a very good match to the CO-versus-(N) relationships predicted using BF best-fit parameter values, uniquely and specifically at the best-fit value of (L) (Figure 12C).

BF simulations should (and do) accurately predict CO homeostasis in interference-defective mutants: CO homeostasis relationships should be altered in mutants where interference is defective, in a predictable way according to the magnitude of the reduction (Figure 12ABC, grey lines above the curves describing the wild-type relationships). We will present elsewhere data showing that BF best-fit simulations for a yeast mutant specifically defective in interference accurately predict CO homeostasis relationships in that mutant, thus further supporting the validity of BF simulation analysis.

#### The strength of CO homeostasis reflects the ratio of inter-precursor distance and interference distance (L_CoC_)

It would be convenient to have a standard way of comparing different situations (e.g. different chromosomes, mutants or organisms) with respect to the “strength” of CO homeostasis. In principle, the strength of homeostasis should vary according to the ratio between the CO interference distance (L_CoC_) and the distance between adjacent precursors (roughly given by the number of precursors divided by µm SC length). If this ratio is higher homeostasis will be stronger because a greater fraction of precursors are within the interference distance and thus can be eliminated without effect; and in the limit, reduction of precursor density will have no effect whatsoever. Oppositely, if this ratio is lower; homeostasis will be weaker because a greater fraction of precursors are outside of the interference distance and thus, when eliminated, will directly reduce CO levels; and in the limit, when the interference distance is zero, CO homeostasis is absent. By this criterion, i.e. [L_COC_/average inter-precursor distance from the data in Table 2], the strength of CO homeostasis is the same for all yeast chromosomes (0.3 µm/0.3 µm = 1); are essentially the same for yeast chromosomes as for the Drosophila X chromosome (6 µm/5 µm = 1.1); is significantly greater for grasshopper (28 µm/2.6 µm = 10.8); and is even greater for tomato chromosomes, both groups (11 µm/0.888 µm = 12.5). Systematic exploration of such relationships by BF simulations remains for future studies.

### Part V. The “Obligatory CO”

Regular segregation of homologs to opposite poles at the first meiotic division requires that they be physically connected. During meiosis in all organisms, in at least one sex and usually both, the requisite physical connection is provided by the combined effects of a crossover between non-sister chromatids of homologs and connections between sister chromatids along the chromosome arms. Correspondingly, in such organisms, in wild-type meiosis, every bivalent almost always acquires at least one CO [47]. This first CO that is essential for homolog segregation is often referred to as the “obligatory CO”. In fact, the obligatory CO is simply a biological imperative: the level of zero-CO chromosomes should be low. The CO patterning process, by whatever mechanism, must somehow explain this feature.

In most situations, the frequency of zero-CO bivalents is extremely low (<10^−3^), but higher frequencies also occur in certain wild-type situations as well as in certain mutants (below). In some models for CO patterning, the obligatory CO is ensured by a specific “added” feature of the patterning process (e.g. the King and Mortimer model; Discussion). In contrast, in the beam-film model, the requirement for one CO per bivalent is satisfied as an intrinsic consequence of the basic functioning of the process, as follows.

#### In the BF model, the “obligatory CO” is independent of (L) and (E) and requires an appropriate combination of values for (Smax), (N), (B) and (M)

**Figure 13:**
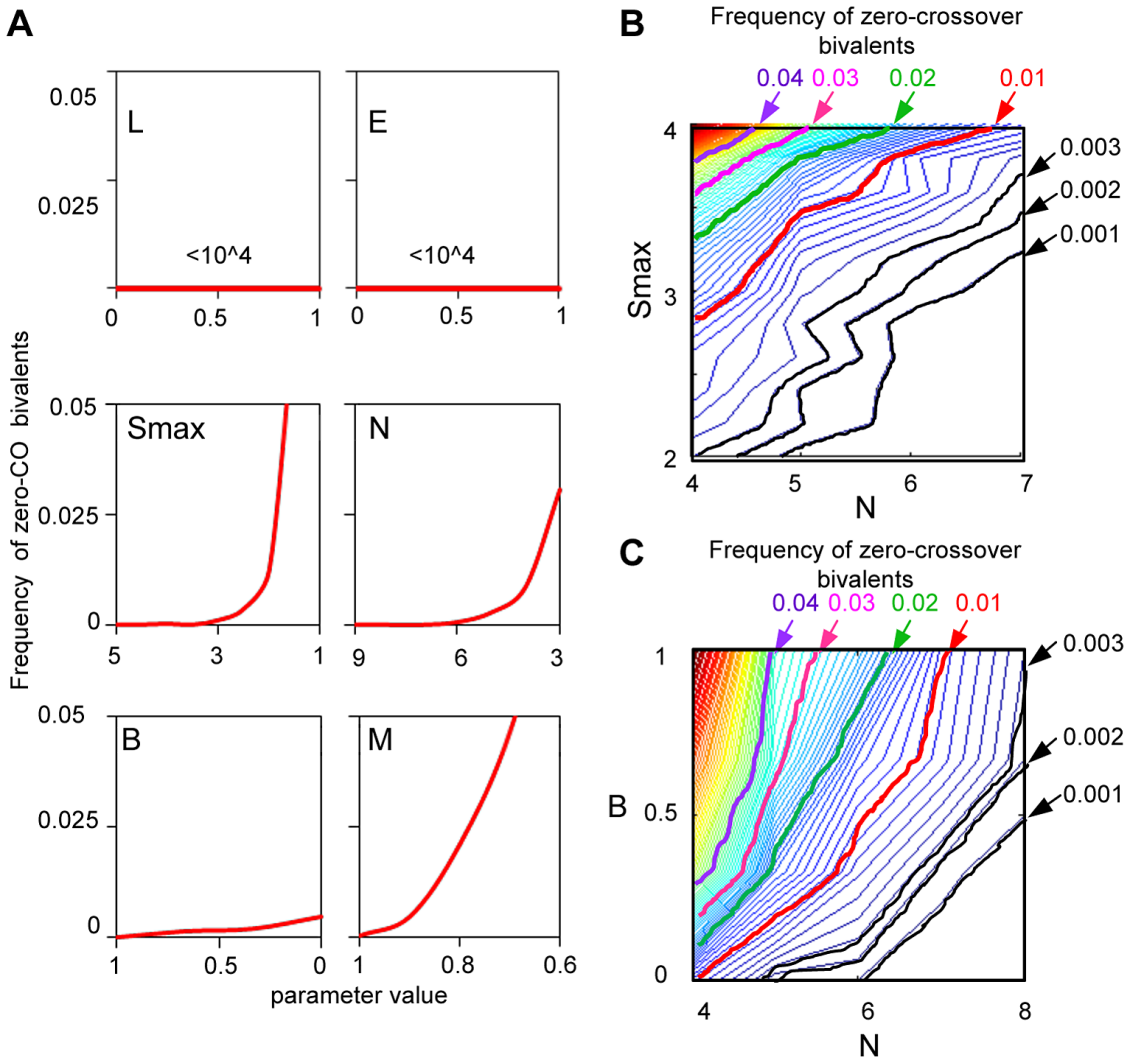
How is a low level of zero-CO bivalents ensured by the beam-film model? (A) BF simulations were carried out under a set of “standard conditions” (as described in Figure 3), except that Smax = 5, N = 8, while the value of one parameter was systematically varied as indicated in each panel. The frequency of zero-CO chromosomes as a function of the value of the varied parameter is plotted. Variations in L and E have no effect; variations in Smax, N, B and M all have effects. (B,C). A given frequency of zero-CO chromosomes can be achieved by diverse constellations of parameter values that play off against one another. This situation is illustrated by BF simulations under above conditions where the frequency of zero-CO chromosomes is determined over a range of combinations of values of two parameters. This interplay is illustrated for combinations of precursor number (N) and either parameter Smax (Panel B) or parameter B (Panel C). The lower the number of precursors, (N), the higher the Smax needed to ensure that at least one will be sensitive enough to undergo CO designation and the more important it will be for precursors to occur at the same average number along the bivalent in every nucleus, thus minimizing the probability that a bivalent will have too-few precursors (higher B) (text).

In the BF model, the obligatory CO is ensured as an intrinsic consequence of all of the features that ensure occurrence of a first event; features that act later in the process are not relevant (Figure 13A).

L and E: Variations in (L) and (E) have no effect on the level of zero-CO chromosomes because: (i) spreading inhibition of CO-designation (“interference”) only affects the number of COs after the first CO designation; and (ii) the distribution of multiple events along a chromosome also comes into play only after the first designation event has occurred. In contrast, essentially all other basic BF parameters are important in ensuring a low level of zero-CO chromosomes:

Smax, N and B: The frequency of zero-CO chromosomes will be minimized if every chromosome has at least one precursor that is adequately sensitive to the DDF. This effect can be favored by either (i) higher Smax; (ii) higher N; or (iii) higher B. Higher Smax means that a higher fraction of sites in a particular precursor array will be adequately sensitive. Higher N means that there will be more sites and there is a higher chance that an adequately-sensitive precursor will be present. Higher B means a reduced probability that a bivalent will have a lower-than-average number of precursors and thus a lower-than-average chance for an adequately-sensitive precursor to be present.

(M): Even if the CO patterning process ensures the occurrence of at least one CO per chromosome, a defect in maturation of CO-designated interactions to detectable COs will tend to counteract that effect, converting chromosomes with one (or a few) COs to chromosomes with zero-COs.

(A): The more likely a precursor is to give a CO-designation in response to a particular local level of interference, the lower will be the frequency of zero-CO chromosomes. The frequency of zero-CO chromosomes is lowest for A = 4 and increases progressively for A = 3, 2 and 1 (not shown).

#### Evolutionary implications

The above considerations imply that, according to the BF model, the “obligatory CO requirement” will be met in any given organism because the relevant features have been coordinately tuned by evolution into a combination that ensures a low level of zero-CO chromosomes. That is: a suitably low level of zero-CO chromosomes can be achieved by a variety of combinations, with more and less favorable values of different parameters in different cases. Interplay between pairs of parameters is illustrated for various combinations of (Smax) and (N) (Figure 13B) and for various combinations of (N) and (B) (Figure 13C). The lower the number of precursors, (N), the higher the Smax needed to ensure that at least one will be sensitive enough to undergo CO designation and the more important it will be for precursors to occur in a constant level along the bivalent in every nucleus (higher B).

#### Application to experimental data: When the obligatory CO appears to be “missing”, in wild-type meiosis

In most organisms, for most chromosomes, in wild-type meiosis the observed frequency of zero-CO chromosomes is <0.1% (e.g. Table 1). However, zero-CO chromosomes occur at significantly higher levels on the Drosophila X chromosome (5%) and on yeast chromosome III (1% by Zip3 foci).

Yeast: the importance of N: BF analysis suggests that, in yeast, the feature responsible for the high level of zero-Zip3 focus chromosomes along chromosome III is simply a paucity of precursors and thus is a function of its diminutive size, *per se*. In this organism, patterns on all analyzed chromosomes can be described by the same set of BF parameters with the exception of (N), which varies roughly in proportion to chromosome length as defined in µm SC (above). The high level of zero-Zip3 focus chromosomes along chromosome III, relative to other chromosomes, is thus solely a reflection of the fact that it is much shorter than other chromosomes (N = 6 versus N≥13; Table 2). This conclusion is directly and strongly supported by experimental analysis of Zip3 foci along chromosome III in mutants where precursor levels are gradually decreased or increased (by alterations in DSB levels; described below). In such mutants, the frequencies of zero-Zip3 focus chromosomes are commensurately increased or decreased. In mutants with relative DSB levels of 1.5, 1 (WT), 0.8 and 0.7, the frequencies of zero-CO chromosomes are, respectively, <0.003; 0.01; 0.02; and 0.08 (Figure S7B).

Analysis of yeast chromosome III further shows that the best-fit simulation requires that precursors occur in a constant number per bivalent in all nuclei (B = 1; frequency of zero-Zip3 focus bivalents = 0.01). If precursors are Poisson distributed among bivalents in different nuclei (B = 0), the frequency of zero-Zip3 focus bivalents increases to 0.04 (Figure S5). This comparison not only suggests that DSBs/precursors always occur at the same number along a different chromosome but provide a rationale for the existence of this feature which, in the general case, is essential to minimize the frequency of zero-CO bivalents along short chromosomes.

Drosophila X chromosome: The high level of zero-CO bivalents for the Drosophila X chromosome is recapitulated in BF simulations without adding any unusual features (Figure 7B), suggesting that there is nothing remarkable about this chromosome. Further, this chromosome the same number of precursors as yeast chromosome III (N = 6), suggesting that here, too, the fact that the chromosome is “too short” could be an important factor for the high level of zero-CO chromosomes. In accord with this possibility, Drosophila chromosome 3 is 50% longer than chromosome X; and BF simulation analysis shows that if X chromosome length is increased by 50%, with a proportional increase in precursors (to N = 9), and without any change in any other parameter, the frequency of zero-CO would decrease to 0.008.

However: the number of precursors cannot be the only relevant feature, because the level of zero-CO chromosomes in Drosophila is higher than that for yeast chromosome III even though they both have N = 6. This difference could be attributable in part to less regular distribution of precursors among chromosomes in Drosophila (B = 0.5 versus B = 1 in yeast) and to a lower DDF level (Smax = 2.8 versus 3.5 in yeast [Table 2]).

Implications: The general implication of these considerations is that CO patterning features have evolved to give a very low level of zero-CO bivalents, in accord with the biological imperative. However, in certain organisms, the constellation of features may be tuned to just such a level that the number of precursors along shorter chromosomes is just at the limit of the necessary threshold.

Notably, also, in both Drosophila and yeast, additional mechanisms exist which complement the patterned CO system to ensure regular homolog segregation. Drosophila exhibits robust CO-independent segregation [48] and, in budding yeast, the significant level of non-interfering COs also ensure disjunction. For example: 1% of chromosome III's exhibit zero Zip3 foci whereas only 0.1% of chromosome IIIs exhibit no COs as defined genetically (Hunter N and Bishop D, personal communication).

#### Application to experimental data: When the obligatory CO appears to be “missing” in mutant meiosis

Mutant phenotypes that affect interference and/or the “obligatory CO” could fall into three different categories [47]:

IF− OC+: The BF model predicts that mutants with decreased CO interference, as defined specifically by decreased L, will show no defect in formation of the first (obligatory) CO, i.e. will show the same level of zero-CO chromosomes as that seen in WT meiosis. This phenotype, “IF− OC+”, not previously reported, is now described by observations in yeast to be presented elsewhere.

IF+ OC−: In certain mutants, the level of zero-CO bivalents is increased but interference is unaltered. In the context of the BF model, this phenotype could arise from several types of defects. For example: this phenotype is expected for mutants with altered recombination biochemistry such that CO designation is normal but CO maturation is inefficient, i.e. M<1 (Figure 5A). For example, mutants lacking Mlh1 exhibit reduced levels of COs and significant levels of zero-CO chromosomes but relatively normal CO interference, as shown by genetic/chiasma analysis for several organisms [49]–[51]. On the other hand, by Zip3 focus analysis, both CoC and ED in a yeast *mlh1*Δ are essentially the same as in WT (Figure 6H), implying that CO-designation is normal. Thus the obligatory CO defect seen by genetic/chiasma analysis is specifically attributable to a maturation defect (M<1). Correspondingly, there are multiple indications that Mlh1 acts very late in recombination, for maturation of dHJs to COs [50], [51].

This phenotype could also be conferred by genetic shortening of a chromosome. A reduction in the Mb length of the chromosome will decrease the precursor number (N) without alternation of any other properties and thus could push N below the minimum necessary threshold. Such an effect could explain the high level of zero-CO chromosomes seen when plant chromosomes are shortened by centric fission [47] and when yeast chromosome III is reduced in length (Hunter N., and Bishop, D.K., personal communication).

IF− OC−: This phenotype could be conferred in several ways. One example would be a defect early in the recombination process that eliminate the ability of recombination precursors to both generate COs and generate/respond to the CO interference signal.

Implications: The existence of IF+ OC− mutants has sometimes been cited as evidence for the existence of a specific feature, separate from interference, that “ensures the obligatory CO”. The existence of IF− OC− mutants has sometimes been cited as evidence that interference is required for the “obligatory CO”. Both of these phenotypes have alternative explanations in the context of the beam-film model. In contrast, the existence of IF− OC+ mutants is specifically predicted by the beam-film model.

### Part VI. Non-interfering COs

In some organisms, a significant fraction of COs arises outside of the patterning process. The existence of these “non-interfering” COs is most rigorously documented for budding yeast, where the number of “non-interfering” COs is ∼30% among total COs (by compassion the number of patterned COs defined by analysis of CO-correlated Zip2/Zip3 foci with the number of total COs from genetic and microarray analyses) (e.g. [22], [31], [52], [53]; below).

**Figure 14:**
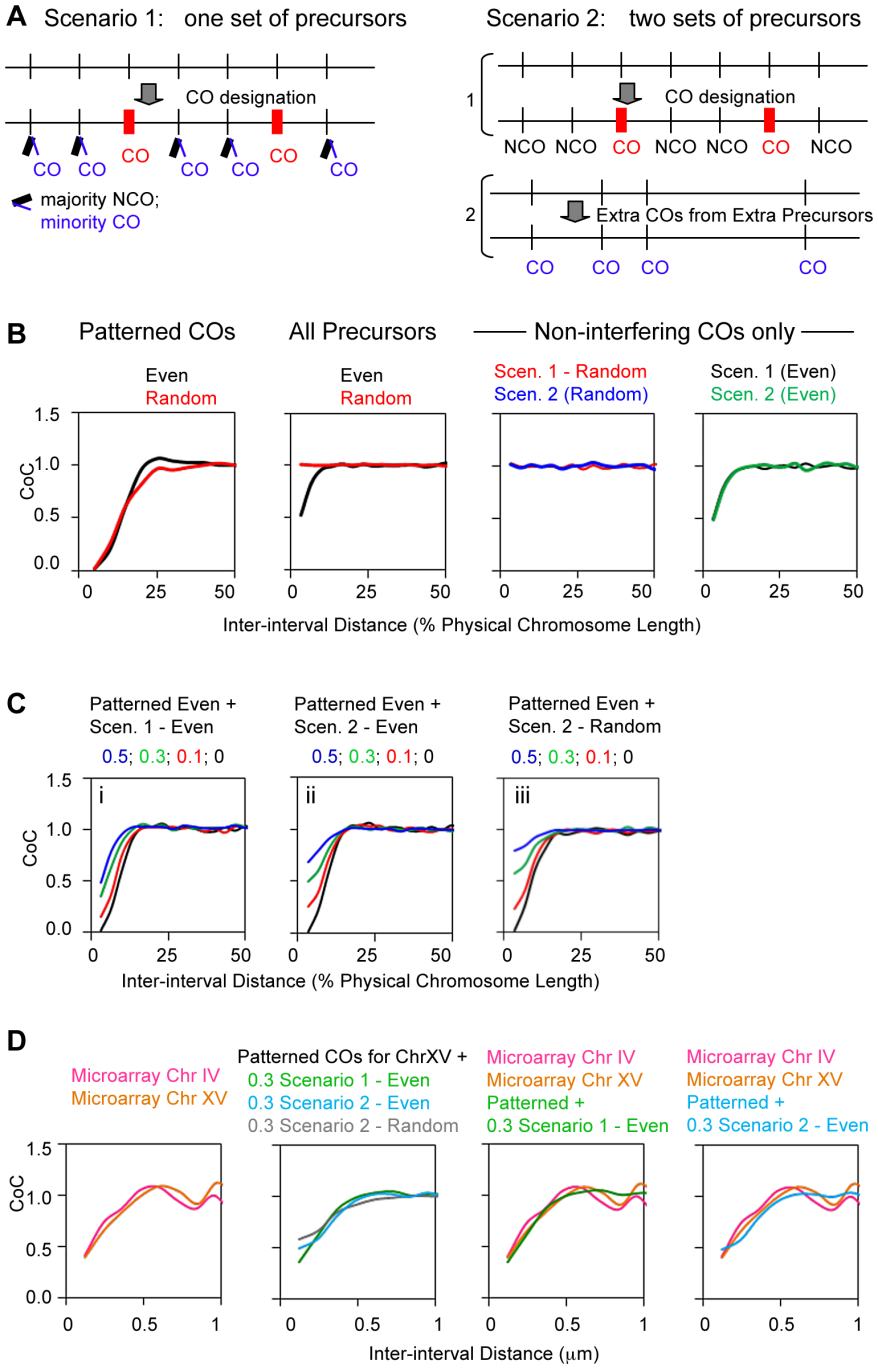
Modeling of non-interfering COs with the example of budding yeast. (A) Non-interfering COs could arise from the same array of precursors as designated COs by low probability of CO formation at remaining not-CO-designated sites (left); alternatively, non-interfering COs could arise from a different set of precursors (right). (B) BF simulations. (Far left) Simulations under standard parameter conditions (Figure 3) illustrate the facts that CoC relationships for interfering COs vary relatively little according to whether precursors are randomly or evenly spaced; see also Figure 3 and Figure S2). (Second from left) An array of precursors was defined under standard parameter conditions with E = 0.6 (relatively even spacing) or random spacing (E = 0) and subjected to a simulation at very high Smax such that all precursors were manifested as “events”, for which CoC curves were then constructed. Precursor CoC relationships are dramatically different for random spacing (CoC = 1 for all inter-interval distances) or even spacing (significant interference at small inter-interval distances). (Right two panels) CoC curves for non-interfering COs generated by Scenario 1 or Scenario 2 with even or random precursor spacing (E = 0.6 or E = 0) directly match the CoC curves for the precursors from which they arose. (C) BF simulations for mixtures of interfering and non-interfering COs, at the different indicated proportions of non-interfering COs. Interfering COs were generated under standard parameter conditions which correspond to yeast chromosome XV (Figure 3; E = 0.6). Non-interfering COs were generated according to Scenario 1 (left) or by Scenario 2 from either even or random precursors (E = 0.6; E = 0) (middle; right). (D) Left: Experimental CoC curves for CO data from microarray analysis of yeast Chromosomes IV and XV [22], [52]. Data from the two studies were combined and the positions of COs defined under the assumption, as made in both studies, that the CO position is at the site of the exchanged polymorphism. CoC analysis of these COs was then performed as for Zip3 foci. Second from left: CoC curves for yeast Chromosome XV corresponding to the combination of interfering COs as described by the best-fit simulation (Figure 6I; E = 0.6) plus non-interfering COs at a level comprising 30% of total COs as described by best-fit simulations under the indicated Scenario and precursor array conditions. Two right-most panels: Experimental CoC curves for Chromosomes IV and XV (from left panel) plus best-fit simulation CoC curves for the indicated mixtures of interfering and non-interfering COs (from the second from left panel). The best match is provided if non-interfering COs arise from precursors left over after CO-designation (Scenario 1 with relatively evenly-spaced precursors; E = 0.6) as shown in second panel from right.

The origin of non-interfering COs is unknown. One possibility is that they arise from the majority subset of interactions that do not undergo CO-designation [5]. By this model (“Scenario 1”; Figure 14A left), not-CO-designated interactions would mostly mature to NCOs but sometimes would mature to COs, analogously to the situation in mitotic DSB-initiated recombinational repair [54]. Alternatively, such COs might arise from some other set of DSBs that arise outside of the normal process, e.g. because they occur later in prophase after CO-designation is completed or earlier in prophase before patterning conditions are established (“Scenario 2”; Figure 14A right).

Both scenarios can be examined using the BF simulation program. To simulate the outcome of Scenario 1, where non-patterned COs arise from non-designated interactions left over after patterning, a standard CO-designation BF simulation is performed to define the interfering COs; the precursors that have *not* undergone CO-designation are then used as the starting array of precursors for a second round of CO-designation. In this second round, COs are randomly selected from among the precursors remaining after the first round of designation. The COs resulting from the two simulations are then combined and the total pattern is analyzed.

To model Scenario 2, in which non-patterned COs arise from an unrelated set of precursors, a standard CO-designation BF simulation is performed to define interfering COs. Then a second, independent simulation is performed using a specified number of precursors that are unrelated to the first set and random selection of COs from among that precursor set. COs generated by the two types of simulations are then again combined and analyzed.

CoC relationships for total COs (interfering plus non-interfering) will depend significantly on whether the precursors that give rise to the “non-interfering” COs are evenly or randomly spaced along the chromosomes. CoC curves for total COs reflect the combined inputs of CoC relationships for interfering COs and non-interfering COs. CoC curves for interfering COs are affected only modestly by even-versus-random spacing due to the overriding effects of CO interference (above; e.g. Figure 14B left). However, non-interfering CO relationships are a direct reflection of precursor relationships, which differ dramatically in the two cases. For precursors, CoC = 1 for random spacing and significant “interference” for even spacing; Figure 14B second from left). CoC relationships for non-interfering COs alone exhibit the same features (Figure 14B, rightmost two panels). These differences are directly visible in CoC curves for total COs, with greater or lesser prominence according to the relative abundance of non-interfering COs versus interfering COs (Figure 14C). Notably, CoC relationships for Scenario 1, where precursors exhibit the even spacing defined by BF best-fit simulations (E = 0.6), show a qualitatively different shape than CoC relationships under Scenario 2.

Given this framework, we defined CoC curves for total COs along yeast chromosomes IV and XV as defined by microarray analysis (Figure 14D left panel). The general shapes of these experimental curves correspond qualitatively to those predicted for emergence of non-interfering COs from an evenly-spaced precursor array, with a closer correspondence to those predicted for Scenario 1 than to those predicted for Scenario 2 (compare Figure 14D left panel with Figure 14C).

This impression is further supported by BF simulations. To model Scenario 1, we began with the set of best-fit parameters defined for interfering COs (Zip3 foci) above (Figure 6I) and generated predicted total CoC curves, assuming that non-interfering COs comprise 30% of the total (above), for each of the three possible case of non-interfering COs: Scenario 1 (where precursors are assumed to be evenly spaced as for interfering COs); and Scenario 2 with precursors assumed to be either evenly or randomly spaced (Figure 14D, second panel from left). The CoC curve for the first of these three cases has the same shape as the experimental CoC curves for total COs (compare Figure 14D left and second from left panels) and direct comparison shows that it gives a quite good quantitative match with the experimental curves (Figure 14D third panel from left). Scenario 2 with evenly-spaced precursors is a less good match (Figure 14D, right panel). Scenario 2 with randomly-spaced precursors (Figure 14D, second panel from left, red) is a quite poor match (not shown).

These analyses suggest that, in yeast, non-interfering COs arise from the not-CO-designated precursors as a minority outcome of the “NCO” default pathway (Figure 14A, Scenario 1).

### Part VII. Variations in Diverse BF Parameters Alter the Value of the Gamma Shape Parameter

**Figure 15:**
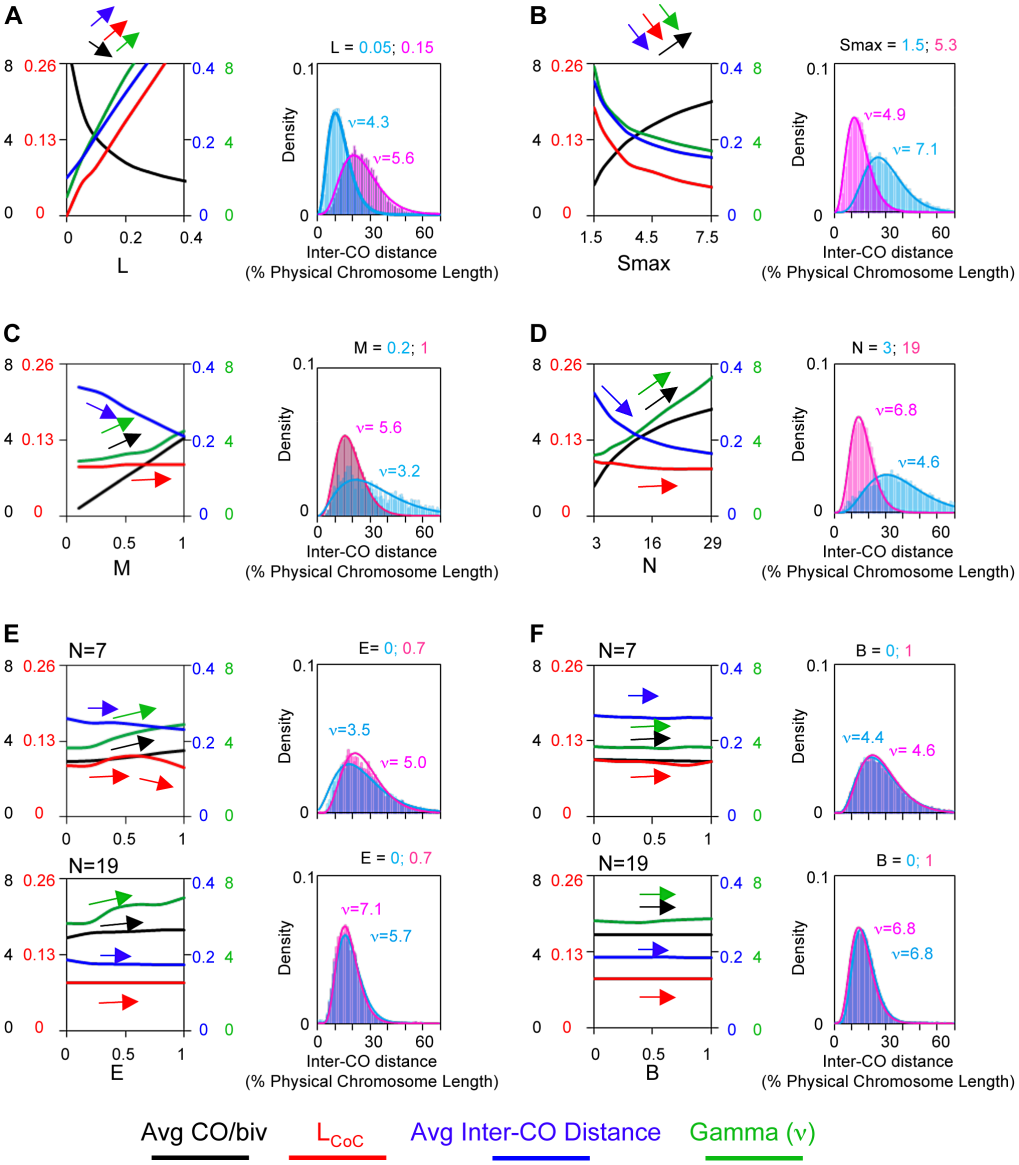
Application of the gamma distribution to CO patterns: Risks and rewards. The value of the gamma distribution shape parameter (ν) varies not only with variations in interference distance (L) but with variations in other parameters. Notably, the value of ν and the value of the average number of COs per bivalent vary inversely with changes in patterning parameters L and Smax but vary directly, or very little, with changes in precursor and maturation parameters. This is shown by BF simulations performed under standard conditions (Figure 3) except that individual parameters were systematically varied. The values of each of the four indicated descriptors (average CO/bivalent; L_CoC_; average distance between adjacent COs (“inter-CO distance”) and the gamma distribution shape parameter ν) were plotted as a function of the value of the varied parameter (left). Also, the distribution of distances between adjacent COs for representative values of the varied parameter are shown (right). (A–D) Effects of systematic variation of parameters L, Smax, N and M. (E, F) Effects of variation of E and B at two different values of N.

Many studies of CO interference characterize CO patterns by defining a gamma distribution that best describes an experimentally observed distribution of the distances between adjacent COs, often with the assumption (implicit or explicit) that a higher value of the gamma shape parameter (ν) corresponds to “stronger” CO interference (e.g. [11]). We have examined the way in which (ν) varies as a function of changes in the values of several BF parameters. Variations in L or Smax increase or decrease the value of (ν) in correlation with increased or decreased L_COC_ and in opposition to the average number of COs per bivalent (Figure 15AB, compare green line and blue/pink distributions with red and black lines). This is the pattern expected for a change in the “strength of interference”. In contrast, the value of (ν) is also altered by variations in M or N, which have little or no effect on L_COC_; moreover, the change in (ν) co-varies with the change in the average number of COs per bivalent (Figure 15CD, compare green line and blue/pink distributions with red and black lines). The BF model thus implies that a change in the value of (ν), e.g. in a mutant as compared to wild type, may or may not imply a change in the patterning process per se. However, comparison of the variation in (ν) with the variation in average COs per bivalent can distinguish between the two possibilities, with opposing variation implying a patterning difference and co-variation implying a difference in some other feature.

## Discussion

The presented analysis has provided new information on CO patterning from several different perspectives.

### BF Simulations Accurately, Quantitatively Describe Experimental Data Sets

This is true not only with respect to CoC and ED relationships but with respect to more detailed effects such as CO homeostasis and the obligatory CO. These matches, and the information that emerges there-from, support the notion that the basic logic of the BF model provides a robust and useful way of thinking about CO patterning. These matches are also specifically supportive of the proposed mechanical stress-and-stress relief mechanism.

### New Information about CO Patterning in Several Organisms

In budding yeast: (i) CO patterning has the same basic features for shorter and longer chromosomes; (ii) Mlh1 is required specifically for CO maturation not for CO patterning; and (iii) Precursors are evenly spaced, as shown by both CoC analysis and analysis of total (interfering-plus-non-interfering) COs.

In tomato (and, to be described elsewhere, in budding yeast), the metric of CO interference is physical chromosome length (µm) not genomic length (Mb). In the case of tomato, differences in CoC relationships expressed in the two different metrics is attributable to differential packaging of heterochromatin versus euchromatin along the chromosome plus differential proportions of heterochromatic versus euchromatic regions among different chromosomes.

In tomato and yeast, as previously described for grasshopper, human and several other organisms, crossover interference spreads across centromeres with the same metric as along chromosome arms.

In budding yeast, non-interfering COs arise from evenly-spaced precursors, most probably by occasional resolution of NCO-fated precursors to the CO fate.

### New Insights into CO Homeostasis and the “Obligatory CO”

With respect to CO homeostasis, the importance of CO interference as a determinant in the strength of homeostasis is emphasized and BF simulations are shown to permit accurate quantitative descriptions of homeostasis. Also, the strength of homeostasis can be seen to reflect the ratio of interference distance (L_CoC_) to the distance between adjacent precursors.

With respect to the obligatory CO, the general logic of the BF model (Figure 1) suggests that occurrence of a low level of zero-CO chromosomes is independent of CO interference (and precursor spacing) and is achieved by an appropriate evolved constellation of all other parameters. Explanations can also be provided for several known cases where the level of zero-CO chromosomes is unusually high, but interference is robust, and potential explanations for other mutant phenotypes are suggested. Importantly, the logic of the beam-film model predicts the existence of mutants that lack interference but still exhibit the obligatory CO, evidence for which will be presented elsewhere.

### Models and Mechanisms of CO Interference

The central issue for CO patterning is how information is communicated along the chromosomes. Three general types of mechanisms have been envisioned. (1) A molecular signal spreads along the chromosomes, e.g. as in the polymerization model of King and Mortimer [55] or the “counting model” of Stahl and colleagues [20], [35]. (2) A biochemical reaction/diffusion process surfs along the chromosomes [56], as recently described in detail for bacterial systems [57], [58]. (3) Communication occurs via redistribution of mechanical stress, as in the beam-film model [3], [4] or via other mechanical mechanisms (e.g. [59]).

The counting model can provide good explanations of experimental data; however, the underlying mechanism is contradicted by experimental findings ([43]; but see [60]). No specific reaction/diffusion mechanism has been suggested thus far for CO interference. The King and Mortimer model and the beam-film model are significantly different, in three respects. First, in the King and Mortimer model, the final array of COs reflects the relative rates of CO-designation and polymerization. Thus it is the kinetics of the system that governs its outcome. In the beam-film model, where interference arises immediately after each CO-designation, kinetics does not play a role. Second, in the King and Mortimer model, the interference signal continues to spread until it runs into another signal approaching from the opposite direction. In the beam-film model, the interference signal is nucleated and spreads for an intrinsically limited distance, with an intrinsic tendency to dissipate with distance from its nucleation site. Third, the King and Mortimer model envisioned that precursors were Poisson distributed among chromosomes. As a result, significant numbers of chromosomes would initially acquire no precursors if the average number of precursors is low and thus would never give a CO, thereby giving an unacceptably high level of zero-CO chromosomes. To compensate for this effect, the model proposed that the effect of interference was to release encountered precursors, which then rebound in regions that were not yet affected by interference (and thus on chromosomes with no precursors). This precursor turnover would ensure that all chromosomes achieved a precursor that could ultimately give a CO. Because of this feature, the King and Mortimer model envisions that interference is required to ensure a low level of zero-CO chromosomes (i.e. to ensure the “obligatory CO”). By the beam-film model, instead, precursors do not turn over and interference is not required to ensure a low level of zero-CO chromosomes, which results instead from an appropriate constellation of other features, as described above. The beam-film model predicts the existence of mutants that are defective in interference but do not exhibit an increase in the frequency of zero-CO chromosomes.

## Materials and Methods

### Yeast Strains

Yeasts SK1 strains (Figure 6 and S7) are described in Table S1. In all strains, ZIP3 carries a MYC epitope tag; a construct expressing LacI-GFP and is integrated at either *LEU2* or *URA3*, and a *lacO* array [61] is inserted at HMR (chromosome III), Scp1 (Chromosome XV) or Chromosome IV telomere (SGD1522198) to specifically label each chromosomes by binding of LacI-GFP.

### Zip2/Zip3 Foci on Yeast Pachytene Chromosomes Correspond to Programmed (“Interfering”) COs

Pachytene chromosomes exhibit ∼65 foci of Zip2, Zip3 and Msh4, with strong colocalization of Zip3 and Msh4 foci ([31], [62]; this work). Zip2 foci [63] exhibit interference as defined by CoC relationships for random adjacent pairs of intervals [31]. We further show here that Zip2 and Zip3 foci exhibit interference as defined by full CoC relationships along specific individual chromosomes (Figure 6 and Figure S5). Zip2 and Zip3 foci also both occur specifically on association sites of *zip1Δ* chromosomes [31], [64]. The total number of COs per yeast nucleus as defined by microarray and genetic analysis is ∼90 [22], [52], [65] implying that Zip2/Zip3/Msh4 foci represent 65/90 = 70% of the total. Correspondingly, mutant analysis suggests that “non-interfering” COs comprise ∼30% of total COs (e.g. [50]). Additionally, BF analysis accurately explains CoC relationships for total COs on the assumption of 70% patterned COs and 30% “non-interfering” COs (Figure 14, Results).

### Cytological Mapping Zip3 Foci in Yeast

Synchronous meiotic cultures (SPS sporulation procedure from [66]) were prepared and harvested at a time when pachytene nuclei are most abundant (∼4–5 hours). Cells were spheroplasted and chromosomes spread on glass slides according to Loidl et al. and Kim et al. [67], [68]. Primary antibodies were mouse monoclonal anti-myc, goat polyclonal anti-Zip1 (Santa Cruz) and rabbit polyclonal anti-GFP (Molecular Probes). Each was diluted appropriately in the above BSA/TBS blocking buffer. Secondary antibodies were donkey anti-mouse, donkey anti-goat, and donkey anti-rabbit IgG labeled with Alexa488, Alexa645 or 594 and Alexa555 (Molecular Probes), respectively. Stained slides were mounted in Slow Fade Light or Prolong Gold Antifade (Molecular Probes). Spread chromosomes were visualized on an Axioplan IEmot microscope (Zeiss) with appropriate filters. Images were collected using Metamorph (Molecular Devices) image acquisition and analysis software. Acquired images were then analyzed with Image J software (NIH), with total SC length and positions of Zip3 foci for the specifically labeled bivalent were measured from the *lacO/*LacIGFP-labeled end to the other end (Figure 6B bottom). For each type of chromosome analyzed (III, IV and XV) in each experiment, measurements were made for >300 bivalents, one from each of a corresponding number of spread nuclei. Resulting data were transferred into an EXCEL worksheet for further analysis.

### CoC Calculations for Yeast Zip3 Foci

Coefficient of coincidence (CoC) curves were generated from SC length and Zip3 focus positions determined as described above. Each analyzed bivalent was divided into a series of intervals of 0.1 µm in length (corresponding to the resolution with which adjacent Zip3 foci can be resolved). Chromosome III, IV and XV were thus usually divided into 9, 42 and 30 intervals with equal size, respectively. Each chromosome length was normalized to 100% and each Zip3 focus position was also normalized correspondingly. Each Zip3 focus was then assigned to a specific interval according to its coordinate. The total frequency of bivalents having a Zip3 focus in each interval was calculated. For each pair of intervals, the frequency of bivalents having a Zip3 focus in both intervals was determined to give the “observed” frequency of double COs. For each pair of intervals, the total CO frequencies for the two intervals were multiplied to give the frequency of double COs “expected” on the hypothesis of independent occurrence. The ratio of these two values is the CoC. Thus in each pair of intervals, CoC = (Obs DCO)/(Pred DCO). CoC values for all pairs of intervals can be plotted as a function of the distance between the midpoints of the two involved intervals (“inter-interval distance”). However, for all of the data shown here, the CoC values from all pairs of intervals having same inter-interval distance were averaged and this average CoC was plotted as a function of inter-interval distance (e.g. Figure 6C and others).

### BF Software and Simulations

The previous Beam Film program [3] was rewritten in MATLAB (R2010a) for easy use and modified to include more features as described in the text. Extensive details regarding program structure and application are provided in the Protocol S1 section. However, briefly, there are three options in the software that serve three different purposes:

1. Analyze existing CO data. This option allows the user to process an experimental CO data set. Outputs include a variety of different CO distribution descriptors including CoC curves, ED relationships including the average number of COs/bivalent, average inter-CO distance and evaluation of the gamma distribution shape parameter (ν).
2. Do a single BF simulation with a particular set of specified parameter values. This option gives the same outputs as for analysis of an existing CO data set. It can help the user to understand how the BF model works (e.g. by extension of examples presented in the Results). It also enables a skilled user to do a single round of BF simulation at some single particular parameter condition.
3. Scan a range of parameters to get a BF best-fit simulation of an experimental data set. This option automatically scans all parameter combinations (over specified ranges of each parameter) and outputs the results in rank order according to the goodness of fit levels calculated based on PLS (Projected Likelihood Score) as defined by Falque et al. [18], [19]. However, the rank order defined by PLS is not a maximum likelihood method. As a result, the best fit judged by PLS is not always the actual best fit. To overcome this drawback, the software outputs the results for all parameter combinations scanned, which the user can further evaluate to select the actual best fit. We normally choose the best fit by comparing experimental and simulated data sets with respect to the CoC curve, the average number of COs per bivalent and the distribution of CO number per bivalents (the ED distribution).

### Other Data Used in This Study

The *Chorthippus L3* chiasmata data were generously provided by Gareth Jones (University of Birmingham, UK). The Drosophila X-chromosome crossover data are from [33]. The tomato (*S. lycopersicum*) Mlh1 foci date are from [38] (generously provided by F. Lhuissier). Zip2 data in the *S.cerevisiae* BR background are from [31] (generously provided by J. Fung).

## Supporting Information

Figure S1 Determination of interval sizes required for accurate CoC curves. Bivalents must be divided into a sufficiently large number of intervals that few if any closely-spaced COs are missed. A general rule is that the interval size should be less than 1/4 the average distance between COs. Operationally, where possible, interval size should be progressively decreased until the position of the CoC curve no longer changes. The simulations presented were performed under standard parameter conditions (text Figure 3) except that the number of intervals (and thus the inter-interval distance) was progressively increased. For this particular case, the CoC curves do not change significantly once the number of intervals is at or above 20 (interval size = 5% total chromosome length in µm).
(TIF)

Figure S2 Interplay among precursor parameter values at low precursor number (N). Variations in the distributions of precursors along or between bivalents (parameters E and B) have more significant effects at lower average precursor numbers (N). Panels (A) and (B): Effects of variations in B and E are illustrated by simulations using the same parameter values as for text Figure 3 except that N = 7.
(TIF)

Figure S3 CoC curves can have signatures that reflect inter-precursor spacing. BF simulations show that if precursors are very evenly spaced (E≥0.8, the corresponding ν>10), and if the interference distance is relatively long as compared to the average distance between precursors (e.g. L = 0.15 versus 0.06–0.17), CoC curves can exhibit “humps” corresponding to the average inter-precursor distance. These humps reflect the fact that closely-spaced double COs will tend to occur specifically at adjacent precursors, and when those precursors are evenly spaced, there is an elevated probability of double CO occurrence at that particular inter-interval distance. This feature is not apparent in standard simulation conditions (text Figures 3–5) because, in those conditions, precursors are less evenly spaced (E = 0.6, the corresponding ν = 2.4). Other BF parameter values for the simulations show in this figure are: L = 0.15, Smax = 1.8, A = 2, cL = cR = 1, E = 0.8, B = 0.9.
(TIF)

Figure S4 How to obtain the best-fit BF simulation for an experimental data set. For each data set, the constellation of BF parameters that provides the best fit to the data set was obtained in three stages, as illustrated for data from yeast Chromosome XV (text). (1) A simulation was carried out at probable approximate values of N and L (Panel A). The range of sensible values of (N) is suggested by total DSB levels, total levels of inter-homolog events (COs+NCOs), numbers of inter-axis bridges, immunofluorescent foci and/or EM-defined SC-associated recombination nodules, all of which approximately reflect total precursor interactions. The initial value of L (L_BF_) was generally set at L_COC_ With respect to other parameters: values of (cL) and (cR) were selected based on the distribution of COs along the chromosome; the value of M was assumed to be 100% for wild-type meiosis; the value of A = 1 was selected as a reasonable first approximation. (2) The value of Smax was then refined so as to optimize the fit between experimental and predicted ED arrays with respect to both the average number of COs per bivalent, including the probability of zero-CO chromosomes (Panel B). (3) The values of all parameters were then further refined by empirical trial-and-error, guided by knowledge as to the general effects of changes in each parameter on CoC and ED outputs as described above. Panel C describes initial refinements with respect to L and Smax; Panel D describes subsequent refinements of these two parameters. The final selected best-fit simulation parameters are those described in Panel D, L = 0.1; Smax = 3.5 (other parameter values in text Table 2).
(TIF)

Figure S5 Experimental data and BF simulations for yeast chromosomes. Panels A–C: CoC and ED relationships, and best-fit BF simulations, for SK1 Chromosomes XV (from text Figure 6I) and analogously analyzed Chromosomes IV and III. Panel D: CoC and ED relationships and BF simulation for Chromosome XIV in the BR background ([31]; J. Fung, personal communication). Parameter values for simulations in A–D in text Table 2. Note that all chromosomes, in both strain backgrounds, have the same CoC relationships when inter-interval distance is expressed in µm SC length. Further, pachytene SC length is ∼10% less in BR than in SK1. This comparison, along with other comparisons (L.Z., unpublished), shows that the metric for interference is physical distance in yeast as in other analyzed organisms (text). Panels E and F: the average CoC curve and the ED relationships for Chromosome III observed experimentally (black) and BF best-fit simulations using optimal parameter values (including E = 0.6 and B = 1, which give relatively even spacing and a constant number of COs per bivalent; Table 2) (red) are compared with BF simulations using the same parameter values except that precursors were considered to be randomly spaced along chromosomes (E = 0; Panel E, green) or Poisson-distributed among chromosomes III in different nuclei (B = 0; Panel F, blue). Even-versus-random spacing affects CoC relationships, confirming that precursors are evenly spaced (text), but does not affect ED relationships. Oppositely, constant-versus-Poisson distribution among chromosomes does not affect CoC relationships but significantly alters ED relationships, with a decrease in the average number of COs per bivalent overall but, more importantly, a significant increase in the frequency of zero-CO bivalents, from 1% to 4%. This effect strongly suggests that a given chromosome always acquires the same/similar number of precursors in every meiotic nucleus.
(TIF)

Figure S6 Heterochromatin/euchromatin ratios underlie differences in SC length in tomato. CoC analysis suggests that the metric for CO interference is physical chromosome length (manifested as SC length) and that the density of DNA, i.e. Mb per µm SC length, is greater for short chromosomes than for longer chromosomes (text). The latter feature reflects the combination of two effects: (i) short chromosomes have a higher percentage of their DNA in heterochromatin and (ii) there is more DNA per µm SC length in heterochromatin versus euchromatin. This conclusion requires knowing: (i) the “packing ratio” of Mb/µm SC length for heterochromatin versus euchromatin; and (ii) the fraction of each pachytene SC length that underlies heterochromatin versus euchromatin. This information is available from published data (Panel A; http://solgenomics.net; [38], [74], [75]. Panel B: (i) Genome-wide, total DNA in heterochromatin and euchromatin are 693 Mb and 203 Mb, respectively and total SC length in heterochromatin and euchromatin are 78 µm and 152 µm, respectively. Thus, Mb/µm SC is 8.9 for heterochromatin and 1.3 for euchromatin, respectively. (ii) For shorter and longer chromosomes, the fractions of SC length in euchromatin and heterochromatin in the two groups are 66% and 34% respectively. This information implies that the group of shorter chromosomes will comprise 70 Mb, with an average Mb/µm SC length of 3.35, while the group of longer chromosomes will comprise 73 Mb, with an average Mb/µm SC length of 4.12. This difference quantitatively explains the offset in CoC curves when inter-interval distance is expressed in Mb versus µm SC length. L_CoC_ (and L_BF_) are 14 µm SC length, which thus corresponds to 47 Mb and 58 Mb, respectively, which is a difference of 11 Mb. The offset in the experimental CoC curves for the two groups when inter-interval distance is expressed in Mb exactly matches this difference: L_CoC_ occurs at inter-interval distances of 32 Mb and 43 Mb respectively, which is a difference of 11 Mb. Notably, these considerations also show that, despite their differences in SC lengths, all of the chromosomes in tomato contain about the same total amount of DNA.
(TIF)

Figure S7 BF simulations of CO patterns in yeast mutants with altered DSB levels. CoC and ED relationships for yeast mutants with changed DSB levels and BF best-fit simulations (Panels A, B). Also indicated are the number of DSBs predicted from experimental analysis (relative DSBs levels from pulse-field gels along chromosome III, VII and VIII in a *rad50S* strain background; [43]) and the number of precursors required to give a best fit simulation. Predicted values of (N) and observed levels of DSBs match very precisely for most of the mutants; however, predicted values are slightly but significantly higher than experimental values at the very lowest DSB levels. This could mean that *rad50S* DSB levels are underestimated at low DSB levels; that DSB levels are auto-catalytic such that occurrence of DSBs above a threshold level tends to promote the formation of additional DSBs; or that best-fit simulations do not give precisely the correct values at low DSB levels.
(TIF)

Protocol S1 Instructions for the BF program.
(DOCX)

Table S1 Strains used in this study.
(DOCX)
