# Supplementary figures and images for: Crossover Patterning by the Beam-Film Model: Analysis and Implications
Source: PLoS Genet. 2014 Jan 30;10(1):e1004042. doi: 10.1371/journal.pgen.1004042 (PMC3907302; doi:10.1371/journal.pgen.1004042)

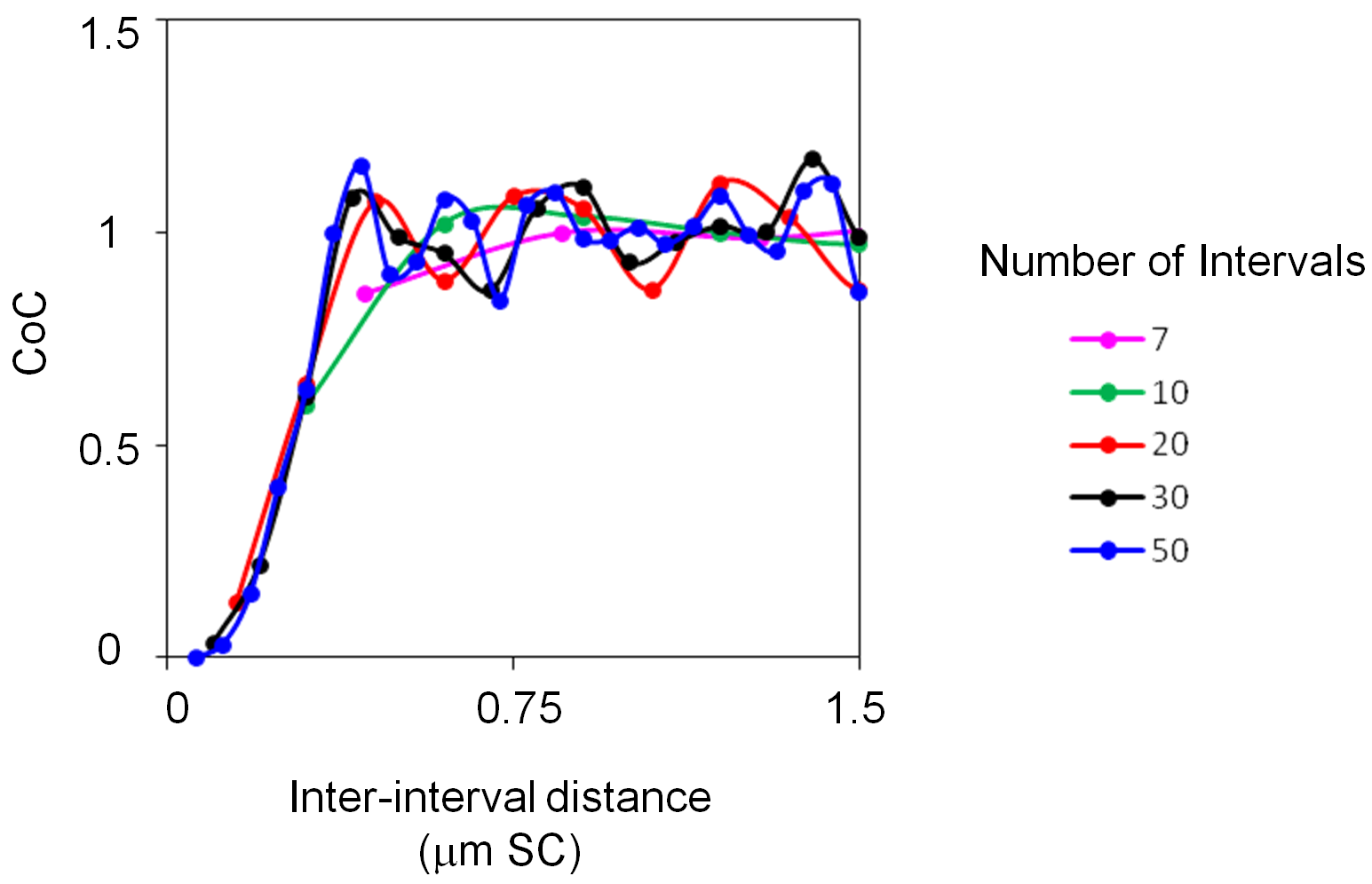

Supplement: Figure S1 — Determination of interval sizes required for accurate CoC curves. Bivalents must be divided into a sufficiently large number of intervals that few if any closely-spaced COs are missed. A general rule is that the interval size should be less than 1/4 the average distance between COs. Operationally, where possible, interval size should be progressively decreased until the position of the CoC curve no longer changes. The simulations presented were performed under standard parameter conditions (text Figure 3) except that the number of intervals (and thus the inter-interval distance) was progressively increased. For this particular case, the CoC curves do not change significantly once the number of intervals is at or above 20 (interval size = 5% total chromosome length in µm). (TIF) [file pgen.1004042.s001.tif]

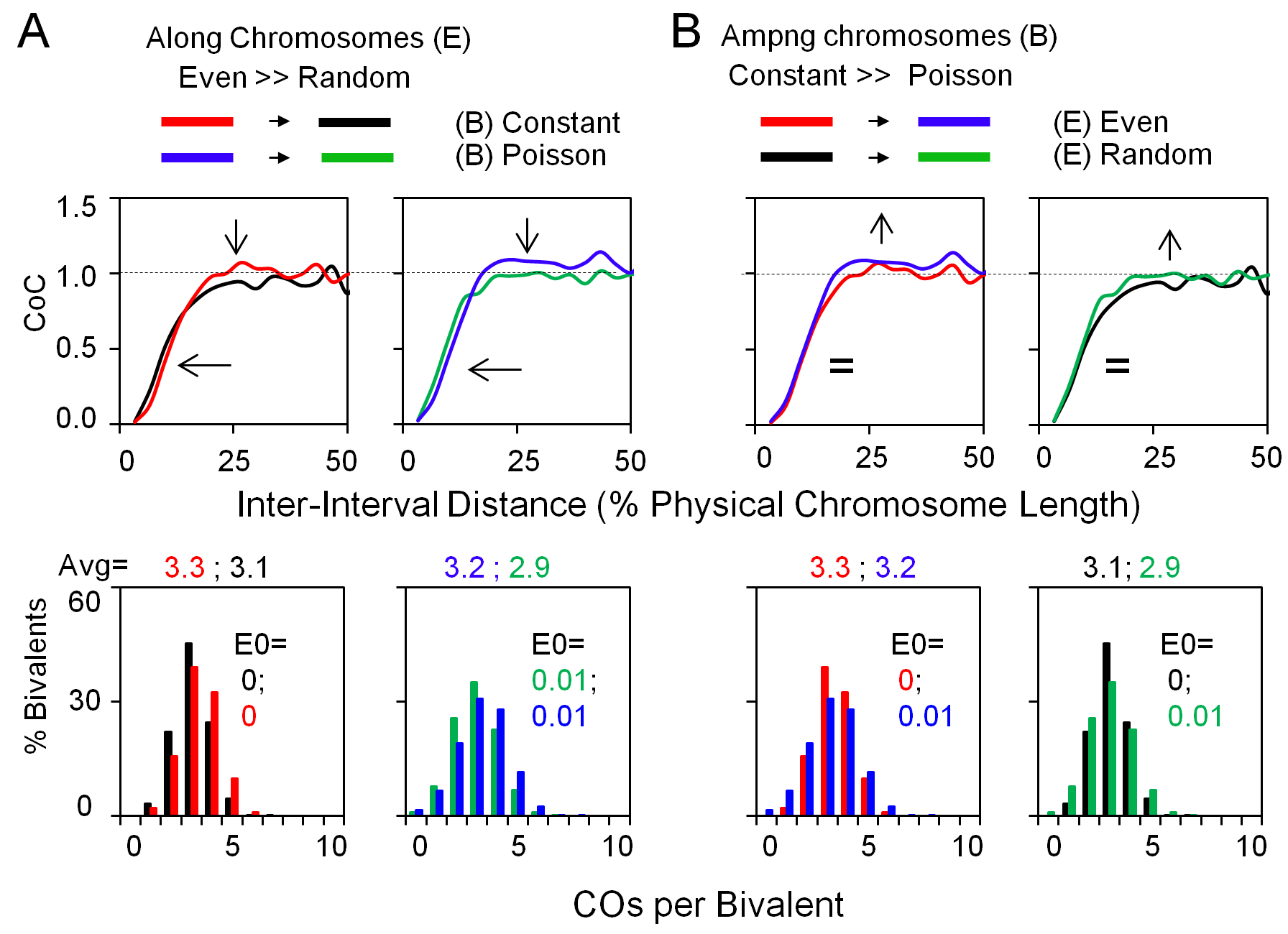

Supplement: Figure S2 — Interplay among precursor parameter values at low precursor number (N). Variations in the distributions of precursors along or between bivalents (parameters E and B) have more significant effects at lower average precursor numbers (N). Panels (A) and (B): Effects of variations in B and E are illustrated by simulations using the same parameter values as for text Figure 3 except that N = 7. (TIF) [file pgen.1004042.s002.tif]

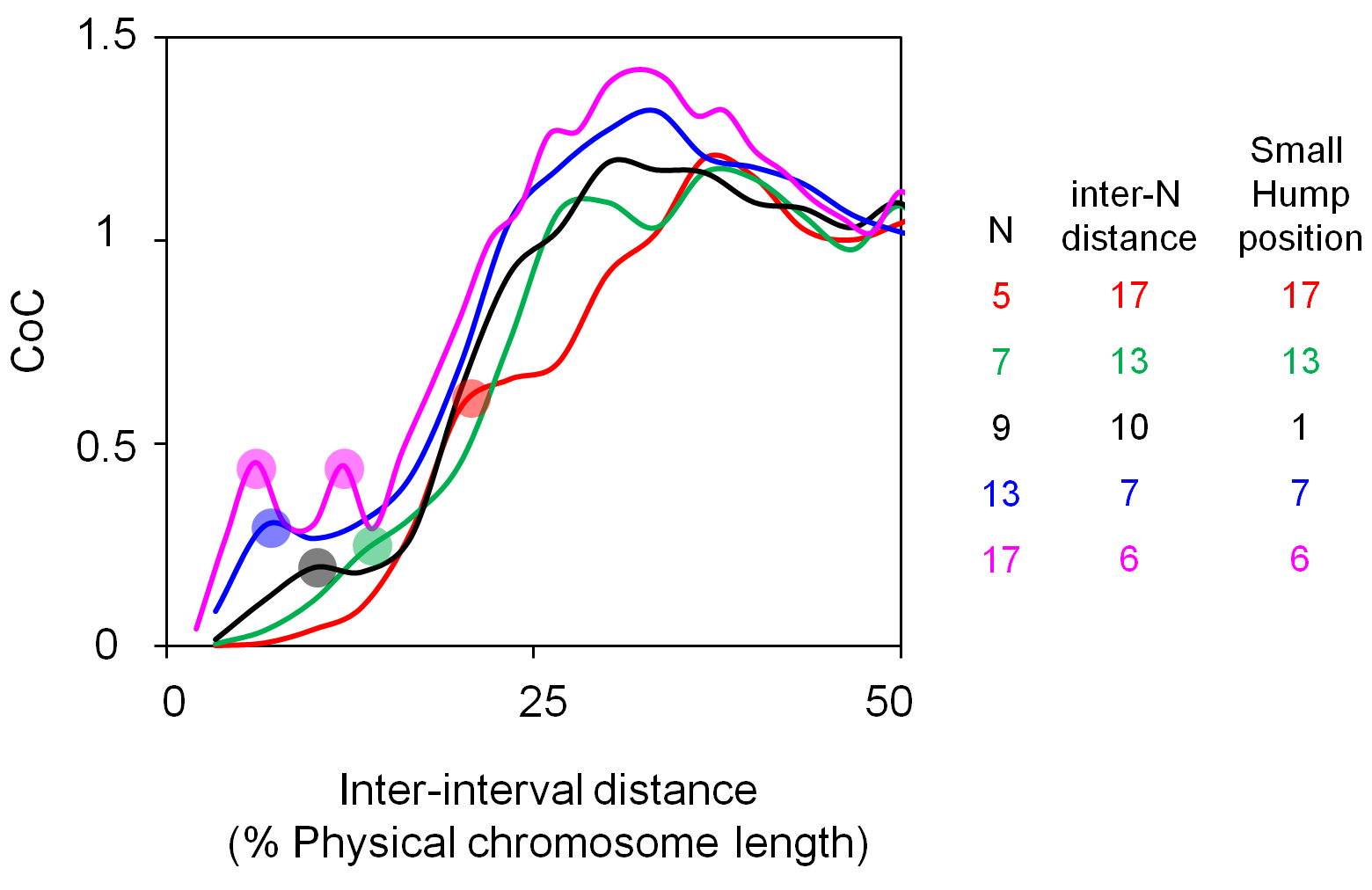

Supplement: Figure S3 — CoC curves can have signatures that reflect inter-precursor spacing. BF simulations show that if precursors are very evenly spaced (E≥0.8, the corresponding ν>10), and if the interference distance is relatively long as compared to the average distance between precursors (e.g. L = 0.15 versus 0.06–0.17), CoC curves can exhibit “humps” corresponding to the average inter-precursor distance. These humps reflect the fact that closely-spaced double COs will tend to occur specifically at adjacent precursors, and when those precursors are evenly spaced, there is an elevated probability of double CO occurrence at that particular inter-interval distance. This feature is not apparent in standard simulation conditions (text Figures 3–5) because, in those conditions, precursors are less evenly spaced (E = 0.6, the corresponding ν = 2.4). Other BF parameter values for the simulations show in this figure are: L = 0.15, Smax = 1.8, A = 2, cL = cR = 1, E = 0.8, B = 0.9. (TIF) [file pgen.1004042.s003.tif]

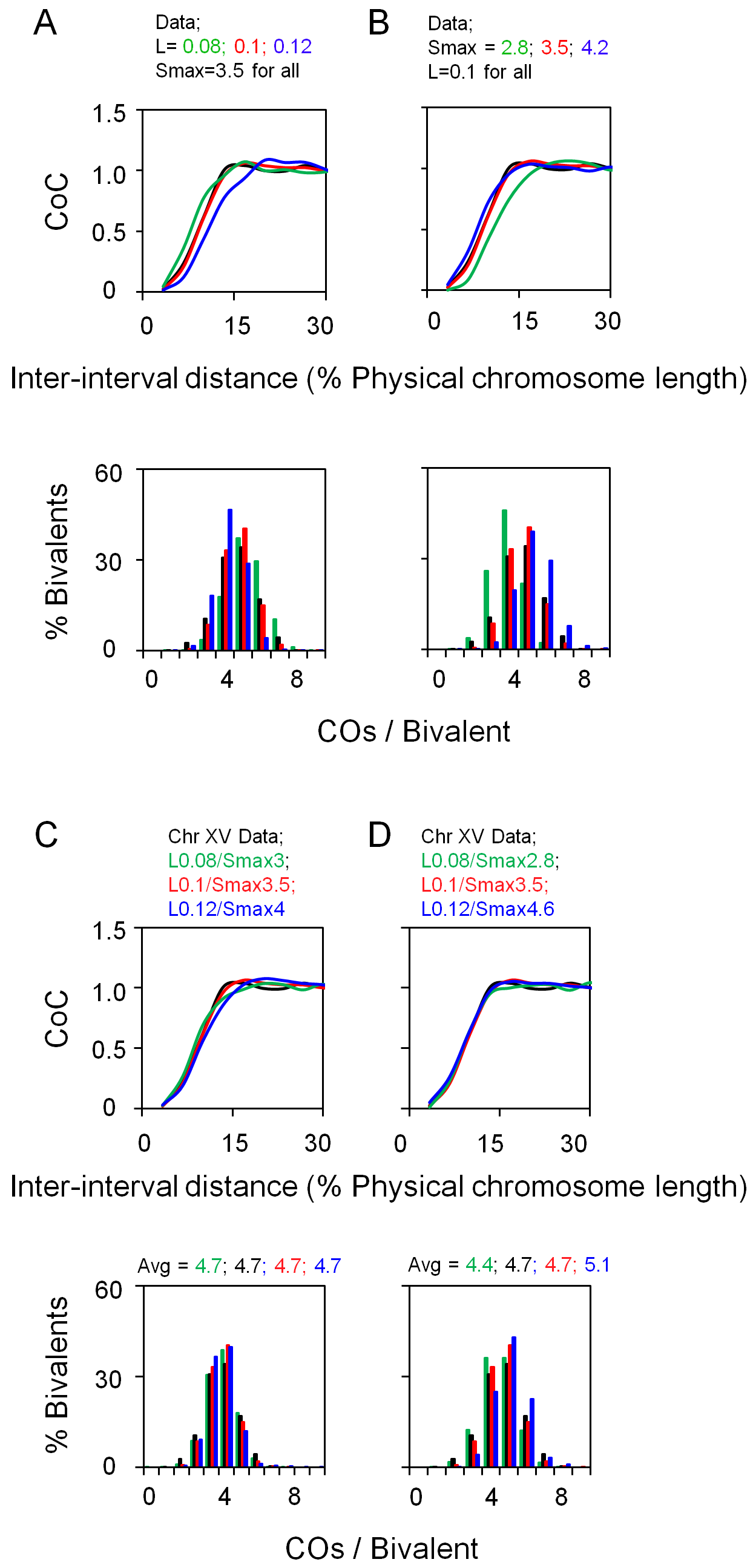

Supplement: Figure S4 — How to obtain the best-fit BF simulation for an experimental data set. For each data set, the constellation of BF parameters that provides the best fit to the data set was obtained in three stages, as illustrated for data from yeast Chromosome XV (text). (1) A simulation was carried out at probable approximate values of N and L (Panel A). The range of sensible values of (N) is suggested by total DSB levels, total levels of inter-homolog events (COs+NCOs), numbers of inter-axis bridges, immunofluorescent foci and/or EM-defined SC-associated recombination nodules, all of which approximately reflect total precursor interactions. The initial value of L (LBF) was generally set at LCOC With respect to other parameters: values of (cL) and (cR) were selected based on the distribution of COs along the chromosome; the value of M was assumed to be 100% for wild-type meiosis; the value of A = 1 was selected as a reasonable first approximation. (2) The value of Smax was then refined so as to optimize the fit between experimental and predicted ED arrays with respect to both the average number of COs per bivalent, including the probability of zero-CO chromosomes (Panel B). (3) The values of all parameters were then further refined by empirical trial-and-error, guided by knowledge as to the general effects of changes in each parameter on CoC and ED outputs as described above. Panel C describes initial refinements with respect to L and Smax; Panel D describes subsequent refinements of these two parameters. The final selected best-fit simulation parameters are those described in Panel D, L = 0.1; Smax = 3.5 (other parameter values in text Table 2). (TIF) [file pgen.1004042.s004.tif]

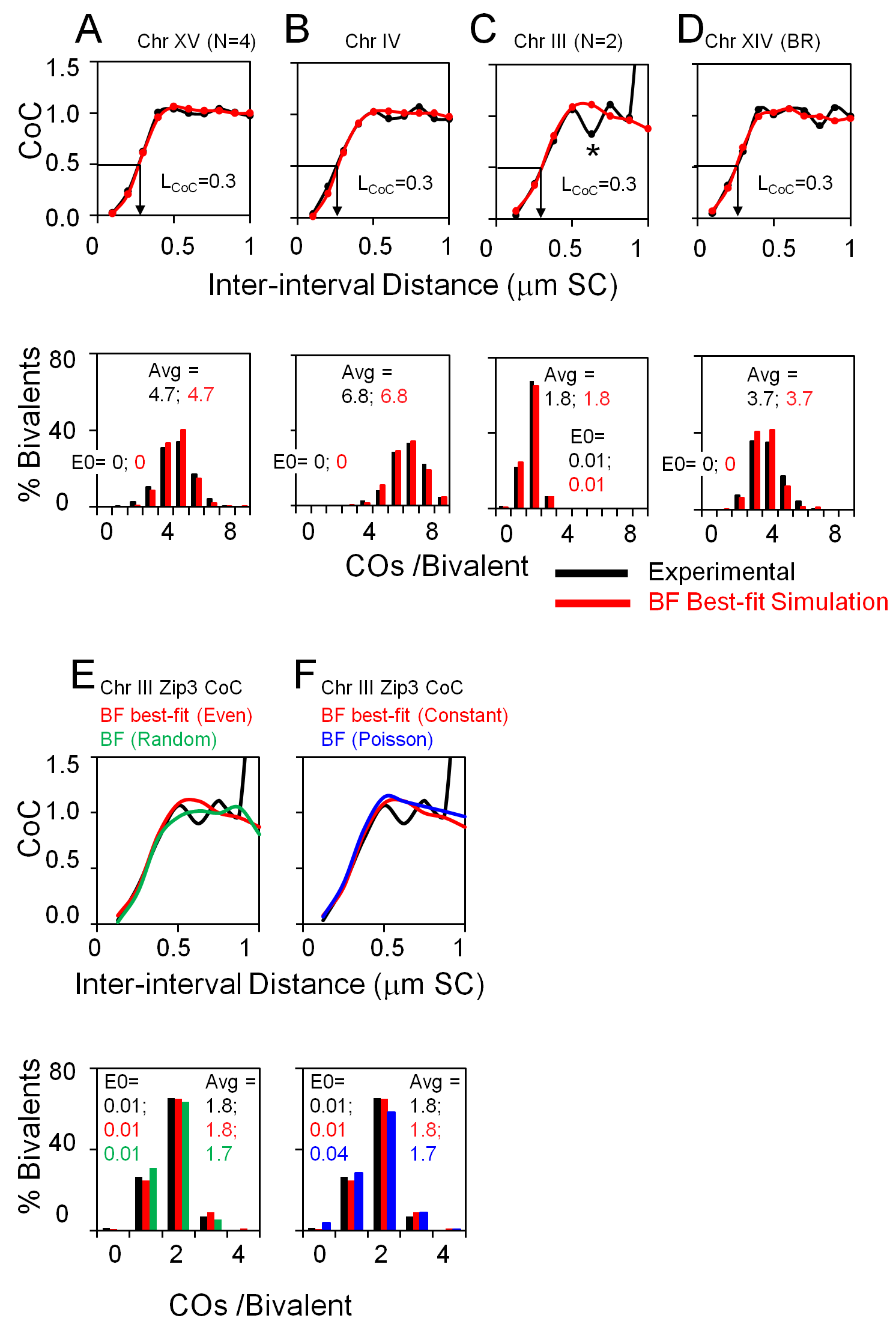

Supplement: Figure S5 — Experimental data and BF simulations for yeast chromosomes. Panels A–C: CoC and ED relationships, and best-fit BF simulations, for SK1 Chromosomes XV (from text Figure 6I) and analogously analyzed Chromosomes IV and III. Panel D: CoC and ED relationships and BF simulation for Chromosome XIV in the BR background ([31]; J. Fung, personal communication). Parameter values for simulations in A–D in text Table 2. Note that all chromosomes, in both strain backgrounds, have the same CoC relationships when inter-interval distance is expressed in µm SC length. Further, pachytene SC length is ∼10% less in BR than in SK1. This comparison, along with other comparisons (L.Z., unpublished), shows that the metric for interference is physical distance in yeast as in other analyzed organisms (text). Panels E and F: the average CoC curve and the ED relationships for Chromosome III observed experimentally (black) and BF best-fit simulations using optimal parameter values (including E = 0.6 and B = 1, which give relatively even spacing and a constant number of COs per bivalent; Table 2) (red) are compared with BF simulations using the same parameter values except that precursors were considered to be randomly spaced along chromosomes (E = 0; Panel E, green) or Poisson-distributed among chromosomes III in different nuclei (B = 0; Panel F, blue). Even-versus-random spacing affects CoC relationships, confirming that precursors are evenly spaced (text), but does not affect ED relationships. Oppositely, constant-versus-Poisson distribution among chromosomes does not affect CoC relationships but significantly alters ED relationships, with a decrease in the average number of COs per bivalent overall but, more importantly, a significant increase in the frequency of zero-CO bivalents, from 1% to 4%. This effect strongly suggests that a given chromosome always acquires the same/similar number of precursors in every meiotic nucleus. (TIF) [file pgen.1004042.s005.tif]

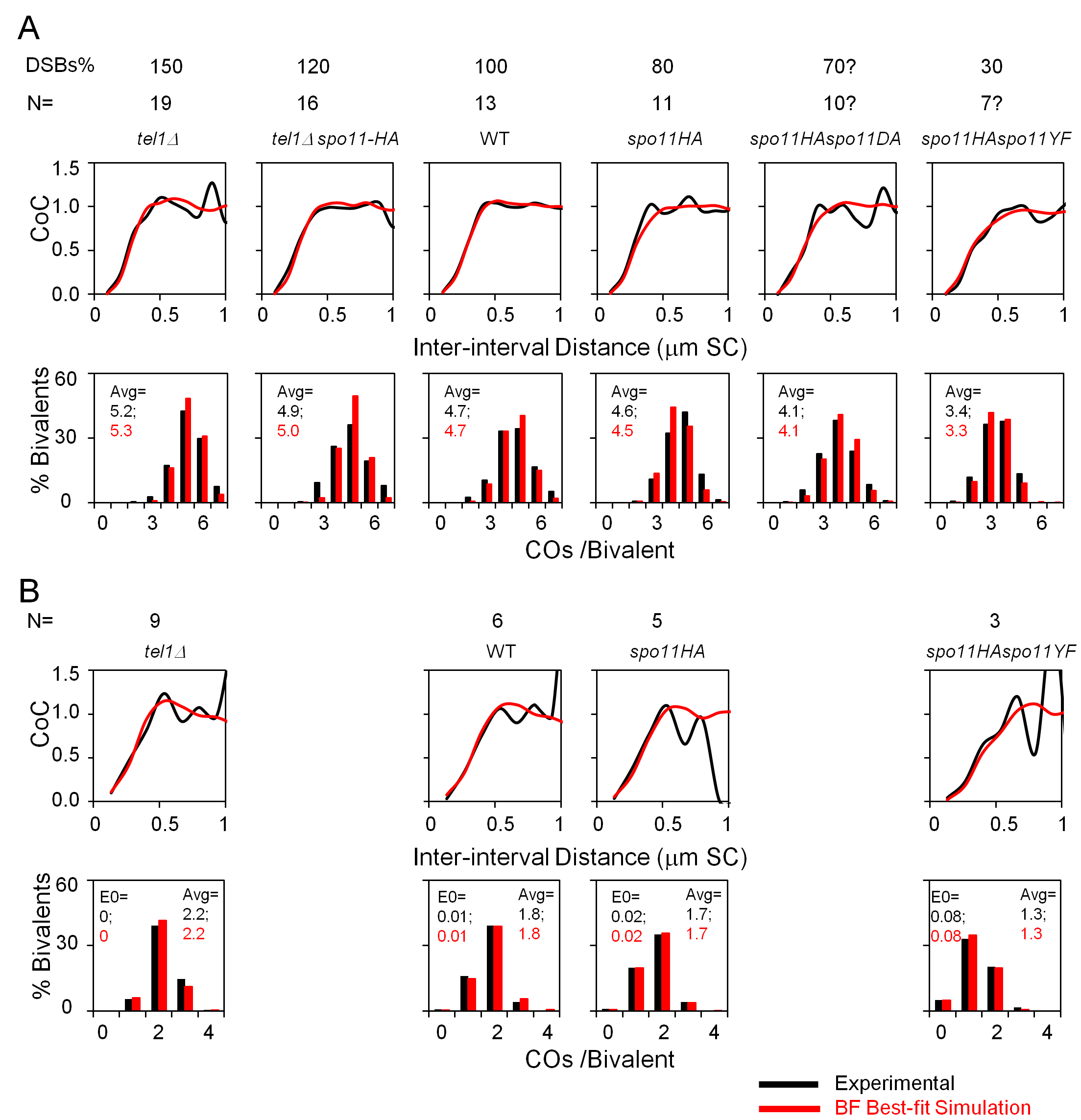

Supplement: Figure S7 — BF simulations of CO patterns in yeast mutants with altered DSB levels. CoC and ED relationships for yeast mutants with changed DSB levels and BF best-fit simulations (Panels A, B). Also indicated are the number of DSBs predicted from experimental analysis (relative DSBs levels from pulse-field gels along chromosome III, VII and VIII in a rad50S strain background; [43]) and the number of precursors required to give a best fit simulation. Predicted values of (N) and observed levels of DSBs match very precisely for most of the mutants; however, predicted values are slightly but significantly higher than experimental values at the very lowest DSB levels. This could mean that rad50S DSB levels are underestimated at low DSB levels; that DSB levels are auto-catalytic such that occurrence of DSBs above a threshold level tends to promote the formation of additional DSBs; or that best-fit simulations do not give precisely the correct values at low DSB levels. (TIF) [file pgen.1004042.s007.tif]
