# Supplementary material for: Crossover Patterning by the Beam-Film Model: Analysis and Implications
Source: PLoS Genet. 2014 Jan 30;10(1):e1004042. doi: 10.1371/journal.pgen.1004042 (PMC3907302; doi:10.1371/journal.pgen.1004042)

**Instructions for BF program**

The new version of BF (Beam Film) code is written in MATLAB (R2010a) for easy use (both the original code and the executable program are downloadable with the following link: https://app.box.com/s/hv91q2nrtq0cp9n8iy9m). Users can use, distribute or modify it freely.

The purpose of this software is to help researchers quantitatively analyze experimental CO data sets and to simulate CO patterns according to the predictions of the BF model.

**1. User interface of the software: general instructions**

Three analysis options are available: (A) Analyze an existing CO data set; (B) Carry out a single round of BF simulation under a specific set of BF parameters; and (C) Scan a range of parameters to get the best fit of the CO data. These three options appear in the main window (a simple graphical user interface; Protocol Figure 1A). After one option is selected, a corresponding next window will appear (Protocol Figure 1BCD).

After selecting one of the three options, a job is initiated by specifying appropriate parameters and, for option A, to input an experimental data set. In the job window that appears upon selection of a particular option (above), when the mouse cursor is moved onto each item, a short explanatory message pops out (e.g. Protocol Figure 1A, mouse cursor symbol and pop out message). For further detailed explanation of these items, consult the remainder of this Instruction (below) and also the main paper.

Once all necessary inputs have been entered and the "RUN" button is pushed, the program will carry out the requested analyses for each of 5000 independent chromosomes. This value is more than sufficient for all uses we have encountered thus far and thus is embedded in the program; however, it can be changed if needed by entering and altering the software itself.

**Note**: Once a particular job has been run, the results can be saved for later use before to running another job, otherwise they will be lost. Results can be preserved by copying them into another location (e.g. another file or a new folder). If results of a job are not saved in this way, they will be substituted by results of the next job in the current work folder.

**2. Option (A): Analyze CO data.**

**Inputs.** The user should first specify the values for two input parameters, specifically:

*1. Interval number of CoC.*  This input defines the intervals used for CoC analysis. The chromosome can be divided either into a particular number of intervals of identical length or a particular array of intervals, of any number, and position along the chromosome can be specified. Same-length intervals are more appropriate for simulation of cytological data where the analyzed chromosome can be divided into intervals as desired. However, for genetic data, intervals are usually specified by particular genetic markers and thus are not all of the same size, so individual specification is required.

In both cases, the necessary specification is entered into the box "Number of intervals for CoC analysis" (Protocol Figure 1B).

For same-length intervals, all that is required is to specify the desired total interval number per chromosome. The default value is 5.

To specify particular intervals, first the genetic marker positions must be normalized. There are two ways to do this normalization according to nature of the data set. (a) If the markers span the entire length of the chromosome, and their positions relative to the chromosome ends are known, the entire chromosome length is defined as "1" and all marker positions correspondingly normalized. (b) If markers define only a continuous internal segment of a chromosome, then the first marker position is defined as “0” and the last marker position is defined as “1” and other marker positions correspondingly normalized. Then, in either case, a list of the normalized genetic marker positions, framed by a pair of square brackets (“[ ]”), is entered into the appropriate box. For example in text Figure 7, the 9 genetic marker positions would be specified as: [0.013, 0.165, 0.249, 0.335, 0.483, 0.58, 0.765, 0.869, 0.958].

Note: BF simulations include parameters that define specialized effects at chromosome ends (cL) and (cR) (text). For simulations, the values of these parameters must be specified appropriately. In the case of option (b), the optimal cL and cR mean effects either from the chromosome region(s) outside the fragment analyzed or from ends (or combined effects) since the ends positions are unknown.

**Note re choice of interval number.**  For CO data sets based on cytological analysis of CO-correlated immuno-stained foci or nodules, the following criteria should be considered in order to set the interval number properly. (a) The interval size should be greater than the microscope resolution (>=0.1 micron for standard epifluorescence or confocal microscopy). (b) Along an individual chromosome, the inhibitory effect of CO interference decays with increasing distance from the site of a CO. As a result, the CoC (classical indicator of CO interference) decreases with increasing inter-interval distance. Thus, a smaller interval size (larger interval number) is preferred because it permits more sensitive detection of interference over smaller inter-interval distances. Also, when precursor interactions are evenly spaced, the value of the CoC tends to rise up to a value larger than one before fluctuating around one (e.g. text Figure 6J) and this feature is more sensitively revealed with smaller inter-interval sizes (text Figure S1). On the other hand, with a smaller inter-interval size, more data (for more bivalents) are needed to get a statistically meaningful result. Thus, a big interval size may require less data but may miss the chance to see short inter-interval interference and CoC rise above one (e.g. text Figure S1). (c) As a general guideline, if possible, interval size should be less than ~1/4 the average distance between adjacent COs. Fewer intervals would tend to place more than one COs into a single interval on a same chromosome which would artificially reduce the CoC. (d) A very large interval number (e.g. 50) will make the calculation process more time-consuming. Conclusion: where possible, the number of intervals should usually be set at five to ten times the CO number.

As an example of these effects, text Figure S1 shows CoC curves with different number of intervals, respectively. The hump where the CoC curve rises above one can be seen with 20 or more interval number, but it is barely seen with <=10 intervals. Also, the position of this feature and its prominence are different with different interval number. A bigger interval size (smaller interval number) would average the details from several smaller intervals (bigger interval number), thus obscuring the effects of interest.

*2. Interval number for rank plots.* Rank plot analysis defines the positions of successive COs along sets of bivalents exhibiting 1, 2 or 3 COs (example in text Figure 8). Analysis of bivalents exhibiting more than 3 COs is not informative because patterns are difficult to discern. For rank plot analysis, chromosomes (bivalents) are divided into intervals and the frequencies of COs of each type are plotted as a function of the interval. If no number is entered, the default value is 0, which means that no rank plots analysis will be performed.

*3. Entering experimental data set.* After input of the two above values, the user should push the “RUN” button. The program will now ask the user to choose the file and the worksheet containing the data to be analyzed. Data must be in an EXCEL file with proper format as described in Protocol Figure 2. For virtually all analyses, chromosomes of a particular genetic identity are first normalized to length 100% and each CO position is then normalized correspondingly.

**Outputs.** The results of running this option are presented in output 3 files:

(1) “output_CO” file. This file includes most of the output information including CO number per chromosome, average CO number, CoC, etc, as detailed in Protocol Figure 3A.

(2) “gamdata_CO” file. This file contains three worksheets. (a) All inter-CO distances for all adjacent pairs of COs are in a worksheet named “gamdata”. (b) Cumulative curve data, which describe the cumulative number of COs as a function of distance from the defined "initial" end, are in worksheet “cumulative”. The primary data are in the second column. The first column is the data for normalized X-axis plot data. (c) The distribution of inter-CO distances, sorted into a specified number of bins, is in worksheet “bin”. The default is 20 bins, but the user can change that number in the bin function.

(3) “coc_CO” file. This file contains the all data relating to CoC relationships which are, for all pairs of intervals: observed double COs (Obs DCOs); predicted double COs (Pred DCOs); and the CoC (= Obs DCO/ Pred DCOs).

**3. Option (B): A single BF simulation.**

This option provides many different outputs for a single set of specified BF parameters. This is useful in helping to understand how the BF model works and, once the set of possible parameter values can be narrowed down by experience or by application of Option (C) below, for doing a single round of BF simulation with all possible outputs.

Users may also need to refer to the fuller discussions in the main text (not referenced specifically below); guidelines concerning how to set the number of intervals along a bivalent presented above under “Analyze CO data” and Section 6 below "More hints for obtaining the best fit between a BF simulation and an experimental data set."

**Inputs**

*(1) CO interference distance (****L****).*  All bivalent length are normalized to “1”, and usually 0<= L <=1. An intrinsic consequence of this feature is that even when different chromosomes of a given organism (and sex) have the same actual value of L in a biological metric (e.g. μm SC), the best-fit value of L will be different for different chromosomes, in proportion to chromosome length by that metric. In some organisms with very strong CO interference, L could be set as > 1. *C.elegans* could be such a case since chromosome fusion studies indicate that the CO interference extends beyond the length of a normal whole chromosome [1]. In organisms with where L is large relative to total chromosome length, the CoC curve rises prominently to values greater than one. As the value of L increases, this feature becomes more prominent and shifts to larger inter-interval distances (to the right on the CoC curve). These effects are illustrated in text Figure 2B and 4A. In arriving at a best-fit simulation it is useful to keep in mind that the inter-interval distance at which the CoC curve exceeds one is generally greater than the value of L. When the average number of COs per bivalent is greater than two, the CoC curve usually reaches a value of one at an inter-interval distance that is about half of the average inter-CO distance. If the average COs per bivalent is less than two, the corresponding point in the CoC curve occurs at an inter-interval distance that is closer to the average inter-CO distance. As a good estimation of the interference distance L, use the inter-interval distance where CoC=0.5 (L_CoC_) from the CoC curve (text for more details).

*(2) CO-designation driving force (****DDF,*** ***Smax****).* Smax is the maximum strength of driving force for CO designation and could correspond to both magnitude and/or time (text for more details). *In vivo*, the parameter Smax could represent a driving force that increases over time, or equivalently, represents increased time duration for CO-designation as proposed for the "interchromosomal effect" in Drosophila ([2,3] ; text). Increased (Smax) has effects on CO number and patterning that resemble those conferred by decreased (L) (see text Figure 4). For organisms examined thus far, the best-fit value of Smax is between one and five. The actual value is easily determined after a range is established for the values of (L) and (N) (below). The BF program also includes an item “Smaxstd” below the Smax item that allows the user to specify different Smax values for different nuclei.

*(3) End conditions (cL and cR).*  This parameter can be used to modulate the different end effects for CO formation in different organisms and/or different chromosomes. The two ends can be set differently. The default value is 0 for both ends (cL=0, cR=0). This default equates to defining the end as a pre-existing CO, with a corresponding reduction in the probability of CO-designation. The effect is to reduce the frequency of COs in the immediate vicinity of the end (over some distance given by the value of (L)). At the other extreme, the value 1 (cL=1, cR=1) means that the end is no different from any other region. However, since CO interference will enter a terminal region from only one side, the consequence is an increase in the number of COs in near-terminal regions relative to internal regions. For example, in some organisms such as *S.cerevisiae*, the frequency of COs (but not NCOs) is suppressed preferentially in telomere regions [4- 8]. This effect is simulated in the BF model by using a low clamping level. However, in other organisms/ chromosomes with a tendency for COs to occur sub-telomerically (e.g. in grasshopper; main text Figure 8), the clamping level could be set at a high level (and may even above 1) to mimic these special terminal effects.

*(4) Precursor parameters.*  Five parameters are used to describe the precursor array: (N), (E), (B), (A) and "black holes". (N) is the average number of precursors per bivalent. (E) is the extent to which precursors are evenly-versus-randomly distributed along each given chromosome. (B) is the extent to which precursors are distributed at a constant number, or are Poisson-distributed, among different chromosomes. (A) specifies the distribution of sensitivities of precursors to the CO driving force. "Black holes" describe cases in which the average number of precursors per unit length varies along the length of a chromosome.

- (N) can be experimentally determined or estimated (at least in some organisms) either by physical DSBs mapping (e.g. [7]) or cytological markers such as Rad51/Dmc1 foci, H2AX foci, RPA foci (e.g. [9-11]), or genetically counted CO and NCO numbers especially from microarray experiments (e.g. [12]). In *S.ceresiae* the precursor number on each chromosome can be more accurately determined based on comprehensive evaluation results from DSBs mapping (e.g. [7]), microarray (e.g. [12]) and classical genetic measurements (http://www.yeastgenome.org). Thus the number of precursors on chromosome III, IV and XV are defined as 6, 19, and 13 respectively. This is about 2~3 times the number of interference COs on these chromosomes (text; Table 2). In mammals, the number of precursors is about 10 times the number of COs (e.g. [9]). In higher plants this ratio is higher, perhaps up to 20-30 folds (e.g. in maize ~20 CO/nucleus and ~500 Rad51 foci/nucleus [13,14]). Due to intrinsic technical limitations, any single assay may not give the accurate precursor number and in some organisms there is absence of data to determine the number of precursors. However, in lots of organisms, particularly those with a high ratio of precursors to COs, a rough estimate of N can work very well. For example, in simulating results for grasshopper (text Figure 8H), the value of N was set at 14; however, we did not see obvious difference for N = 8 – 25. On the other hand, for organisms with a low number of precursors and/or a low N/CO ratio, variations in N have more dramatic effects on CoC and ED (CO number/distribution) values (see Figure S2). Thus, specifying (N) as accurately as possible helps to accurately define the strength of interference.

- The parameter (B) makes it possible to model the extent to which a given (genetic) chromosome gets different number of precursors in different nuclei. By changing the value of (B), the user can vary this condition from a random distribution (Poisson distribution; B = 0) to a constant number (B = 1), where the number of precursors for a given chromosome is the same in all nuclei. When the number of precursors is high, the value of (B) has little effect on ED (CO number/distribution) or on CoC relationships. However, at low values of N, more random distribution of precursors among different chromosomes (nuclei) will increase the fraction of chromosomes that fail to get even one precursor (or, more generally, even one precursor that is adequately sensitive to the CO driving force define by Smax). This will lead to an increase in the frequency of chromosomes that fail to acquire even a single CO (zero-CO chromosomes or E0's; text Figure 13, Figure S2B and S5F). Correspondingly, there are indications that the precursors tend to occur at a relatively constant level along a given chromosome in different nuclei. For example, in *C. elegans*, there are only a small number average of precursors, but every chromosome in every nucleus gets at least one DSB [15]. In Sordaria, precursor numbers defined by Rad51 and Mer3 foci are relatively similar for each chromosome among different nuclei [11]. Thus usually B >> 0.

- The parameter (E) sets the precursor distribution along each given chromosome. The value of E is between 0 and 1. The default value is 0, i.e. randomly distributed precursors, which is set by the MATLAB function “rand” based on Poisson distribution in [0, 1] interval. E = 1 means absolutely evenness. Between these two extremes, evenness levels are adjusted by varying the standard deviation relative to the mean of [the distance between adjacent precursors], which in turn is given by the average precursor number N. A commonly-used metric for defining "evenness of spacing) is the shape parameter of the gamma distribution ("ν"). To relate values of (E) to this metric, the distribution of inter-precursor distances at different values of (E) were subjected to gamma distribution analysis. Roughly, E = 0 corresponds to ν=1; E = 0.6 corresponds to ν =2.4; E = 0.7 corresponds to ν = 4.5 and at E=1, ν is infinite. Users can obtain the values of ν corresponding to any specific value of (E) from the BF output file under the “Precursor Gamma” item.

Specification of the value of (E) is important. In most cases, the expectation is that E >>0 because, recently, there is more and more evidence, from studies in various organisms, that precursors are distributed with some level of evenness. For example: (i) In Sordaria, total precursors marked by Mer3 or Msh4 foci are arranged along chromosomes like peas in a pod, with a ν value >200 ([11] and discussion therein). (ii) In mouse meiosis, Msh4 and RPA foci, which are also believed to label total inter-homolog recombination intermediates, are distributed non-randomly (ν = ~4 - 5; [10,16]). (iii) In *S.cerevisiae*, there is interference not only between COs but between COs and NCO [12]. Moreover, evidence for evenly distributed precursors can be detected as early as DSBs stage in other types of analysis [17].

Correspondingly, implementation of evenly distributed precursors in our model, which is never considered/ included in any other model, is very helpful for researchers to understand/distinguish the interplay between precursor "interference" and CO interference. Simulations illustrate the fact that effects of even spacing of precursors can be seen in CO patterns when the level of N is relatively low (as seen e.g. for Drosophila X or *S. cerevisiae* chromosome III; text Table 2; text Figure S2A). When the value of N is relatively high, the effects of CO patterning override the effects of even spacing of precursors (text Figure 3B).

- (A). The sensitivity (s) of each precursor to the CO driving force is randomly assigned with the MATLAB function “rand”. Our original model assumed that the value of (A) is the square root of this random number [18]. In the BF model, 4 preset amplitude distributions with different average sensitive levels are ordered from lowest to highest based on this with little modification. Different values of (A) are required for best-fit simulations in different organisms, in the following order. A=1 (*S.cerevisiae, Drosophila*); A= 2 (grasshopper); A=3 (tomato and probably also for other higher plants) (text Table 2).

- For specifying a "black hole" the BF default is [0, 0, 1] which implies no difference in the average frequency of precursors along the chromosome. In this option, the first two numbers define the black hole interval and the third number defines precursor density in the black hole interval compared to the average precursor density along the whole chromosome. Multiple intervals with different average precursor densities can be specified although not incorporated in this program.

Application of this option is most prominently useful to accommodate the fact that, in some organisms or for some chromosomes of a given organism, the centromere appears to suppress DSB formation in a large region around it, which contributes to a reduced COs density in this region (see text for more details). Thus, the black hole option has been set up as a low precursor density in the region surrounding the centomere (e.g. text Figure 8B). Necessarily, and as documented by simulations (e.g. text compare Figure 8F with 8G), introduction of a black hole has no effect on CO interference (for interval pairs in which both intervals actually acquire a significant number of COs).

Application of the black hole and ends-clamping options are very useful features of BF simulations. This is particularly obvious in some organisms such as grasshopper. On the other hand, variations in these features are of negligible significance in some organisms and/or particularly on the long chromosomes of such organisms where ends comprise a relatively small fraction of the total chromosome length.

*(5) “Non-interfering” COs.* Genetic studies indicate that there are two different pathways for CO formation in most organisms. The major pathway in most organisms is subject to patterning via the CO interference process. This pathway is sometimes referred to as the "ZMM" pathway (in *S.cerevisiae* it includes at least Zip1-4, Mer3, Msh4/5, Mlh1/3). In some organisms, e.g. *C.elegans* this is likely the only pathway, at least in WT meiosis. A secondary pathway also appears to give rise to COs, outside of the normal patterning process, resulting in what are referred to as "non-interfering COs" (although this may not be a correct characterization in the strict sense). These COs are thought to comprise 5-30% of total COs among several different species as defined by biological or computational analysis [19]. In some organisms, COs do not exhibit interference (e.g. *S. pombe* and *Aspergillus nidulans*). In such organisms, all COs might arise by the same "non-interfering" pathway present in most other cases, or by some different route.

In the BF model, the effects of non-interfering COs on the total array of COs can be modeled. The nature of the non-interfering CO contribution is specified in three steps. First, one of two possible sources of such COs is selected, either Scenario 1 or 2 (further discussion in text and text Figure 14). In Scenario 1, non-interfering COs arise from the same array of precursors as the patterned COs, by random selection among the set of precursors that is left behind after CO-designation. In Scenario 2, non-interfering COs arise from an independently-established array of precursors which might appear either before or after the precursors that give rise to patterned COs. Second, the final fraction of total COs that arise from that source is specified. For both Scenarios, the default option is zero, i.e. no non-interfering COs. Third, for Scenario 1, the precursor array is automatically defined by the parameters used for CO patterning. However, for Scenario 2, the nature of the precursor array can be set either randomly or evenly distributed along chromosomes (i.e. E=0 or E>0) and the number is either Poisson distributed or more constantly among chromosomes (i.e. B=0, or B>0).

*(6) CO maturation efficiency (M).* The default value of M is 1, which implies that 100% of CO-designated interactions actually finally mature into COs. This condition applies in most cases. However, for some chromosomes in some organisms in wild-type meiosis, and in several mutants, the probability that a CO-designated interaction will emerge as a mature CO/chiasma is not 100% because of some inefficiency or defect in events after the designation step. We describe this situation as a reduction in the efficiency of "CO maturation", the magnitude of which is given by a value of M<1. In the BF program, a defect in maturation is simulated simply by randomly removing some CO designation sites from the primary output array of programmed COs. Necessarily, and as confirmed by BF simulations for an *mlh1D* mutant in budding yeast, CO interference as defined by the CoC curve is unaltered by reductions in the value of M (except at very low frequencies of COs/bivalent).

Experimentally, a defect in CO maturation could be detected by comparing the number and distribution of sites defined by very early marker for CO-designation (e.g. Zip3 foci in *S. cerevisiae*) with those defined by a later marker, e.g. Mlh1/3 foci detected by immunostaining; chiasmata detected at diakinesis or metaphase I; or events detected genetically or by microarray analysis of marker/sequence heterozygosities.

Non-interfering COs (section (5) above) could potentially also exhibit inefficient maturation. This possibility can be mimicked by simply adjusting the fraction of these types of COs specified for the simulation (above). Thus, variations in CO maturation efficiency are specifically applied, as such, only to the programmed COs.

*(7) Number of intervals for CoC analysis.* Please refer the Note in section “Analyze CO data” above for details and guidelines.

*(8) Interval number for rank plot analysis.* Please refer to corresponding section above in Part 2. Option (A): Analyze CO data.

**Outputs.**

The user will be presented with four output files after running a job according to this Option:

(1) “output_BF” contains essentially the same outputs obtained from running a job in Option A“ Analyze CO data” (example in Protocol Figure 3A).

(2) “CoC_BF” contains observed DCOs, predicted DCOs and CoC value for all pairs of intervals as described above for the “coc_CO” output file of Option A “Analyze CO data”.

(3) “precursor_BF” shows the array of precursors.

(4) “co_BF” contains the CO array generated by the simulation run. The array is presented in essentially the same format as that described above of CO data sets to be input into "Analyze CO data" in Option A above except that all chromosome lengths have been normalized to one and the "zeros" are used to designate sites of precursors that did not undergo CO-designation.

This data, after add number “1” (the normalized total bivalent length) as the last column, can be used as a data set for analysis of a simulation output by Option A. Such analysis can confirm the validity of the precursor distribution and provide additional further information (e.g. inter-precursor distances, gamma distribution, etc).

**4. Option (C): Automatically search the best fit**

This option automatically scans all combinations of parameter values over particular specified ranges for goodness-of-fit to an input data set. The results of a scan are presented in an ordered list, ranked according to goodness of fit levels based on a PLS (Projected Likelihood Score) derived from that of Falque et al. [20] but with some improvements (including information concerning events at ends and giving more weight to the importance of the ED (average CO number/distribution)). Importantly, however: this weighting method is not a maximum likelihood method and thus the "best fit" as judged by PLS may not be the true best fit. To overcome this drawback, the software output presents all parameter combinations scanned so that the user can check particular cases individually to get the best fit based on all kinds of criteria. We usually choose the best fit by comprehensively comparing simulated and actual data for average number of COs per bivalent, the CoC curve, and the distribution of CO number per bivalent. As discussed in the main paper, the gamma distribution is not a very good indicator of the strength of CO interference level but can be useful in distinguishing whether a given variation in CO patterning reflects a change in a patterning parameter (L, Smax) or some other parameter (for the precursor array or maturation efficiency). Thus we also use the values of average inter-CO distance and the shape parameter ν as additional inputs when deciding on a best fit.

The scanning provided by Option C is useful, especially for users without extensive prior experience with BF simulations, to provide rough estimates of reasonable parameter values. However, to run a job by this Option is very time consuming. We therefore suggest that users first run a job in which parameters are varied over a large range, but with large step sizes between the evaluated values. This can be followed by a second (and maybe also a third) round in which the range of values is narrowed and the step sizes decreased based on the results of the prior scan(s).

Because this scan is so time consuming, readers must be well-informed as to likely ranges of parameter values (as discussed above and in the main text) before initiating a job.

**Inputs.**

All parameters are same as those in Option B “A single BF simulation”. In addition, the user will have to specify a range and a step size for parameters L, N and Smax. Once these values are specified and the job is started, the user will be asked to select the specific CO data file to be used as the reference (formatting as described for Option A "Analyze CO data".

**Outputs.** The user will be presented with a single output file named “BFscan”. This file contains three worksheets:

(1) *“Zip3” sheet* is the experimental data set to which the program seeks a best fit. This sheet presents: (a) average CO number per bivalent, (b) CO events distribution (ED) and (c) average CoC from the experimental data set.

(2) *“BFcopattern” sheet* includes average CO number and CO number per bivalent (ED) from BF simulation (see Figure 3C for an example output).

(3) *“BFcoc” sheet* includes CoC data from BF simulation (see Protocol Figure 3B for an example output).

**5. Inner structure of the software**

Once all input information has been entered, the program carries out the following tasks in a specific order (which depends somewhat on the option(s) selected, Figure S5 and S6). The details of these progressions are very useful if the user wishes to modify the program.

*(1) Precursor array.* The program first sets up the precursor array from the input values of N, B, E and A as described above. If a black hole for the precursor is set up, the precursor array is then adjusted according to the black hole setting.

*(2) Raw CO array.* Once the precursor array is defined, the program will apply the previously-described beam-film function for CO-designation and interference [18] to each of the specified number of bivalents (beams). This operation defines, for each bivalent, which precursor will be a CO (crack) based on the values of the distance (L) over which inhibiting interference (stress) spreads, the maximal level of CO-designation (stress) (Smax) and the distribution of precursor sensitivities to CO-designation (flaw amplitude) (A). The process reaches its limit when no remaining precursors have an amplitude high enough to respond to the specified maximum CO-designation force (Smax). The final outcome is an array of COs and an array of "leftover" un-reacted precursors, output as described above.

*(3) Adjusted CO array.* The raw CO array is then adjusted according to additional inputs such as maturation efficiency (M) and/or the level of non-interfering COs (above). From this reorganized array, program calculates ED relationships, i.e. the average number of COs per bivalent and the frequencies of bivalents with different numbers of COs; the average inter-CO distance, the distribution of distances between adjacent COs and, based on the latter distribution, shape parameter of the Gamma distribution (ν).

*(4) Finalized CO array.* COs in the reorganized, adjusted CO array are now sorted into the intervals specified for CoC analysis. Each CO is assigned to a specific interval, thus defining a "finalized" CO array.

*(5) CoC.* Given the finalized CO array in (4), the program now calculates, for each pair of intervals: (i) the actual frequency of double COs (Obs DCO); the predicted frequency of double COs (Pred DCO) based on the assumption that COs occur independently in the two intervals; and, finally, the ratio of (Obs DCO)/ (Pred DCO), i.e. the CoC.

*(6) Outputs.* Output files are created, according to the option selected, as outlined above.

**6. More hints for obtaining the best fit between a BF simulation and an experimental data set.**

There are two approaches for arriving at the best-fit BF simulation of an experimental data set. One approach is to use the best-fit scan described above. However, a user with more experience regarding the effects of changes in various parameters should be able to arrive at the best fit more directly by trial-and-error with a constrained set of possible parameter values (see main text for more details).

Additional practical tips for arriving at the best fit:

*(a) Interplay between L, Smax*. Both increased L (or decreased L) and decreased Smax (or increased Smax) can decrease (or increase) CO number and shift CoC curve to right (or left). However the two parameters have distinguishable effects for CoC and CO number as discussed in the text and text Figure 4. Furthermore, as shown in Figure 4 and Figure S4, if both L and Smax are changed coordinately while keeping Event Distribution (ED) relationships (CO number/ distribution) fixed, the CoC curve changes; and if both L and Smax are changed coordinately while keeping the CoC curve is kept constant, the CO number/distribution changes. In summary, it is always possible to find specific optimal values of L and Smax.

*(b) L, N and physical chromosome length.* In a given organism, different chromosomes are of different lengths, both genetically and physically (e.g. at the pachytene stage when CO-correlated foci are examined for patterning). When comparing chromosomes of different lengths in BF simulations, two different considerations must be kept in mind.

First: in the BF model, chromosome length is always defined as 1. Therefore, if interference spreads over a particular physical distance along the chromosome (as appears to be the case; [21,22,23]), and assuming that distance is the same for all chromosomes, the best-fit value of (L) in the BF model will have to be normalized such that the absolute length in physical chromosome distance is the same for all chromosomes. That is, for longer chromosomes, the value of L must be a smaller percent of total chromosome length, and *vice versa* for shorter chromosomes. If this normalization is not performed, different chromosomes will exhibit different values of L (as % total chromosome length) whereas the biological reality is that the interference distance is the same for all chromosomes.

Second: experimental data suggest that the number of precursors along a chromosome is proportional to chromosome length ([15]; L.Z. and N.K. in preparation). For BF simulations, this fact implies that for best fit simulations of chromosomes of different lengths, the value of (N) must be scaled such that (N)/length is a constant. This is important because CoC curves and the number/distribution of COs per bivalent are both sensitive to the number of precursors (text, Figure 3).

When these two considerations are taken together, they imply that correct modeling of genetically different chromosomes of different lengths, in the same organism, requires that both (L) and (N) be appropriately normalized to chromosome length. An example is shown in Protocol Figure 4. Simulations were carried out for a hypothetical set of four chromosomes whose lengths vary over a four-fold range. From largest to smallest, the BF value of (L) is increased four-fold (to provide a constant absolute value per unit actual chromosome length) while the value of (N) decreases four-fold in accord with a constant density of precursors per unit length. All other parameters are kept constant. The average number of COs/bivalent varies in direct proportion to the chromosome length, just as does the average number of precursors per bivalent, as shown in Protocol Figure 4A over a wider range of values of chromosome length. However, the CoC curves for the four types of chromosomes are exactly overlapping (Protocol Figure 4B).

There are also situations in which a chromosome of a given genetic composition exhibits two different physical lengths in two different situations, where the interference distance extends over the same physical length in both cases. Modeling of these situations requires the same two adjustments, for analogous reasons (see text Figure 9).

**7. Running environments**

The following apply to both Windows and Mac operating systems.

(a) .exe (large file) can be run in computers without MATLAB.

(b) Original codes (MATLAB code) works only in a MATLAB window.

**References**

1. Hillers KJ, Villeneuve AM (2003) Chromosome-wide control of meiotic crossing over in C. elegans. Curr Biol 13: 1641-1647.

2. Joyce EF, McKim KS (2010) Chromosome axis defects induce a checkpoint-mediated delay and interchromosomal effect on crossing over during Drosophila meiosis. PLoS Genet 6: e1001059.

3. Joyce EF, McKim KS (2011) Meiotic checkpoints and the interchromosomal effect on crossing over in Drosophila females. Fly (Austin) 5: 134-140.

4. Gerton JL, DeRisi J, Shroff R, Lichten M, Brown PO, et al. (2000) Global mapping of meiotic recombination hotspots and coldspots in the yeast Saccharomyces cerevisiae. Proc Natl Acad Sci U S A 97: 11383-11390.

5. Buhler C, Borde V, Lichten M (2007) Mapping meiotic single-strand DNA reveals a new landscape of DNA double-strand breaks in Saccharomyces cerevisiae. PLoS Biol 5: e324.

6. Blitzblau HG, Bell GW, Rodriguez J, Bell SP, Hochwagen A (2007) Mapping of meiotic single-stranded DNA reveals double-stranded-break hotspots near centromeres and telomeres. Curr Biol 17: 2003-2012.

7. Pan J, Sasaki M, Kniewel R, Murakami H, Blitzblau HG, et al. (2011) A hierarchical combination of factors shapes the genome-wide topography of yeast meiotic recombination initiation. Cell 144: 719-731.

8. Chen SY, Tsubouchi T, Rockmill B, Sandler JS, Richards DR, et al. (2008) Global analysis of the meiotic crossover landscape. Dev Cell 15: 401-415.

9. Cole F, Kauppi L, Lange J, Roig I, Wang R, et al. (2012) Homeostatic control of recombination is implemented progressively in mouse meiosis. Nat Cell Biol 14: 424-430.

10. de Boer E, Stam P, Dietrich AJ, Pastink A, Heyting C (2006) Two levels of interference in mouse meiotic recombination. Proc Natl Acad Sci U S A 103: 9607-9612.

11. Storlazzi A, Gargano S, Ruprich-Robert G, Falque M, David M, et al. (2010) Recombination proteins mediate meiotic spatial chromosome organization and pairing. Cell 141: 94-106.

12. Mancera E, Bourgon R, Brozzi A, Huber W, Steinmetz LM (2008) High-resolution mapping of meiotic crossovers and non-crossovers in yeast. Nature 454: 479-485.

13. Pawlowski WP, Golubovskaya IN, Cande WZ (2003) Altered nuclear distribution of recombination protein RAD51 in maize mutants suggests the involvement of RAD51 in meiotic homology recognition. Plant Cell 15: 1807-1816.

14. Anderson LK, Doyle, G.G., Brigham, B., Carter, J., Hooker, K.D., Lai, A., Rice, M., and Stack, S.M. (2003) High-resolution crossover maps for each bivalent of Zea mays using recombination nodules. Genetics 165: 849-865.

15. Mets DG, Meyer BJ (2009) Condensins regulate meiotic DNA break distribution, thus crossover frequency, by controlling chromosome structure. Cell 139: 73-86.

16. Oliver-Bonet M CM, Turek PJ, Ko E, Martin RH (2007) Analysis of replication protein A (RPA) in human spermatogenesis. Mol Human Reprod 13: 837-844.

17. Zhang L, Kim KP, Kleckner NE, Storlazzi A (2011) Meiotic double-strand breaks occur once per pair of (sister) chromatids and, via Mec1/ATR and Tel1/ATM, once per quartet of chromatids. Proc Natl Acad Sci U S A 108:20036-41.

18. Kleckner N, Zickler D, Jones GH, Dekker J, Padmore R, et al. (2004) A mechanical basis for chromosome function. Proc Natl Acad Sci U S A 101: 12592-12597.

19. Berchowitz LE, Copenhaver GP (2010) Genetic interference: don't stand so close to me. Curr Genomics 11: 91-102.

20. Petkov PM, Broman KW, Szatkiewicz JP, Paigen K (2007) Crossover interference underlies sex differences in recombination rates. Trends Genet 23: 539-542.

21. Petkov PM, Broman KW, Szatkiewicz JP, Paigen K (2007) Crossover interference underlies sex differences in recombination rates. Trends Genet 23: 539-542.

22. Billings T, Sargent EE, Szatkiewicz JP, Leahy N, Kwak IY, et al. (2010) Patterns of recombination activity on mouse chromosome 11 revealed by high resolution mapping. PLoS One 5: e15340.

23. Drouaud J, Mercier R, Chelysheva L, Berard A, Falque M, et al. (2007) Sex-specific crossover distributions and variations in interference level along Arabidopsis thaliana chromosome 4. PLoS Genet 3: e106.


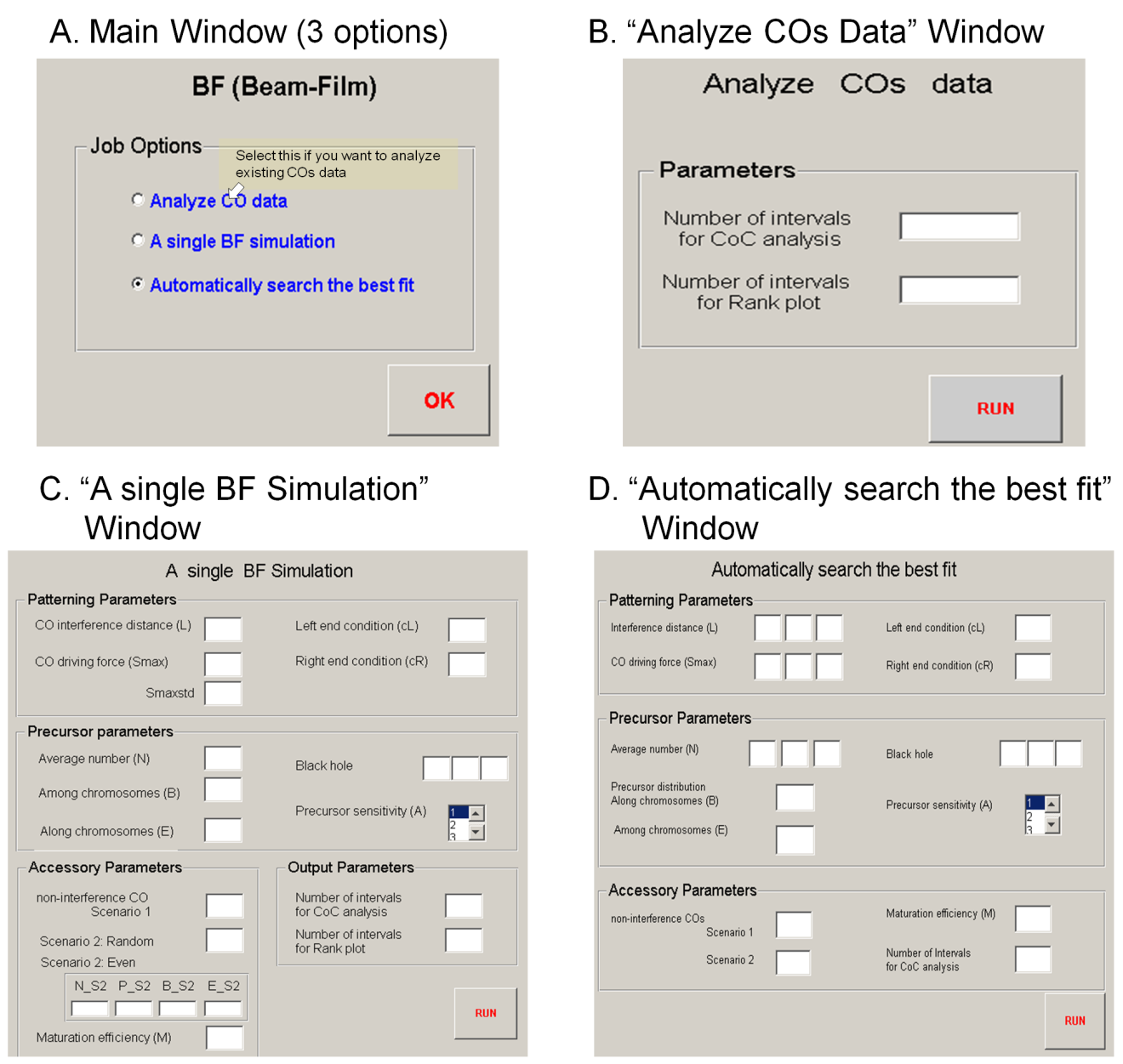


**Protocol Figure 1. BF Software User Interface.**

(A) The main window includes three options (B-D). (B) The sub-window of Option A: “Analyze CO data”; (C) The sub-window of Option B: “A single BF simulation”. (D) The sub-window of Option C: “Automatically search the best fit”.


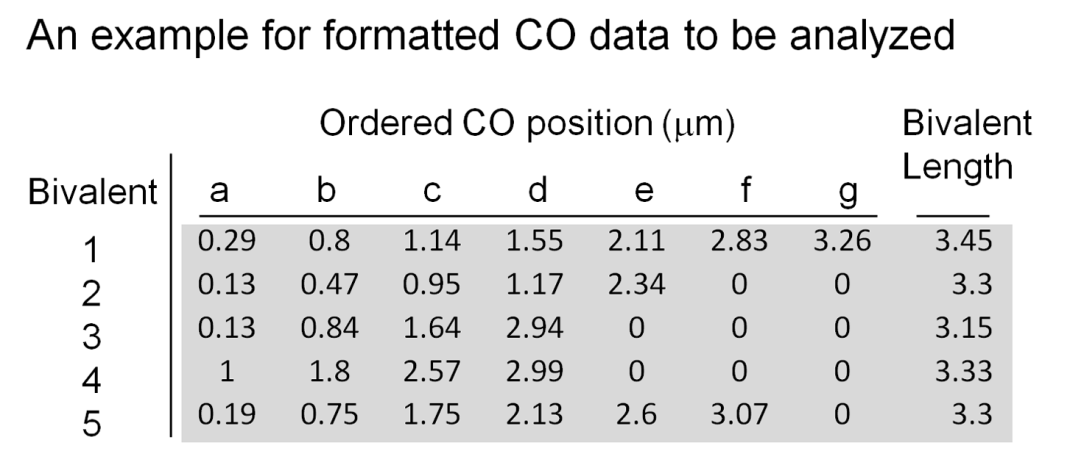


**Protocol Figure 2. An example of the proper format for a data set to be subjected to for CO analysis (Option A) or BF scan (Option C).**

For each bivalent in the data set, CO positions are ordered, beginning at one end defined as 0, from smaller to larger. The last column is the total bivalent length. For bivalents with fewer COs than the maximum number present in the data set, the positions beyond the last CO are filled with zeros, e.g. for the second bivalent, columns f and g are filled with “0”.

**Protocol Figure 3. Output examples.**

(A) An example of the output from Option B: “a single BF simulation” output.

(B, C) Examples of the outputs from Option C: “BFscan”, for worksheets named “BFcoc” and “BFcopattern” respectively. The parts below the dash lines are the actual outputs, and above it are annotations added.


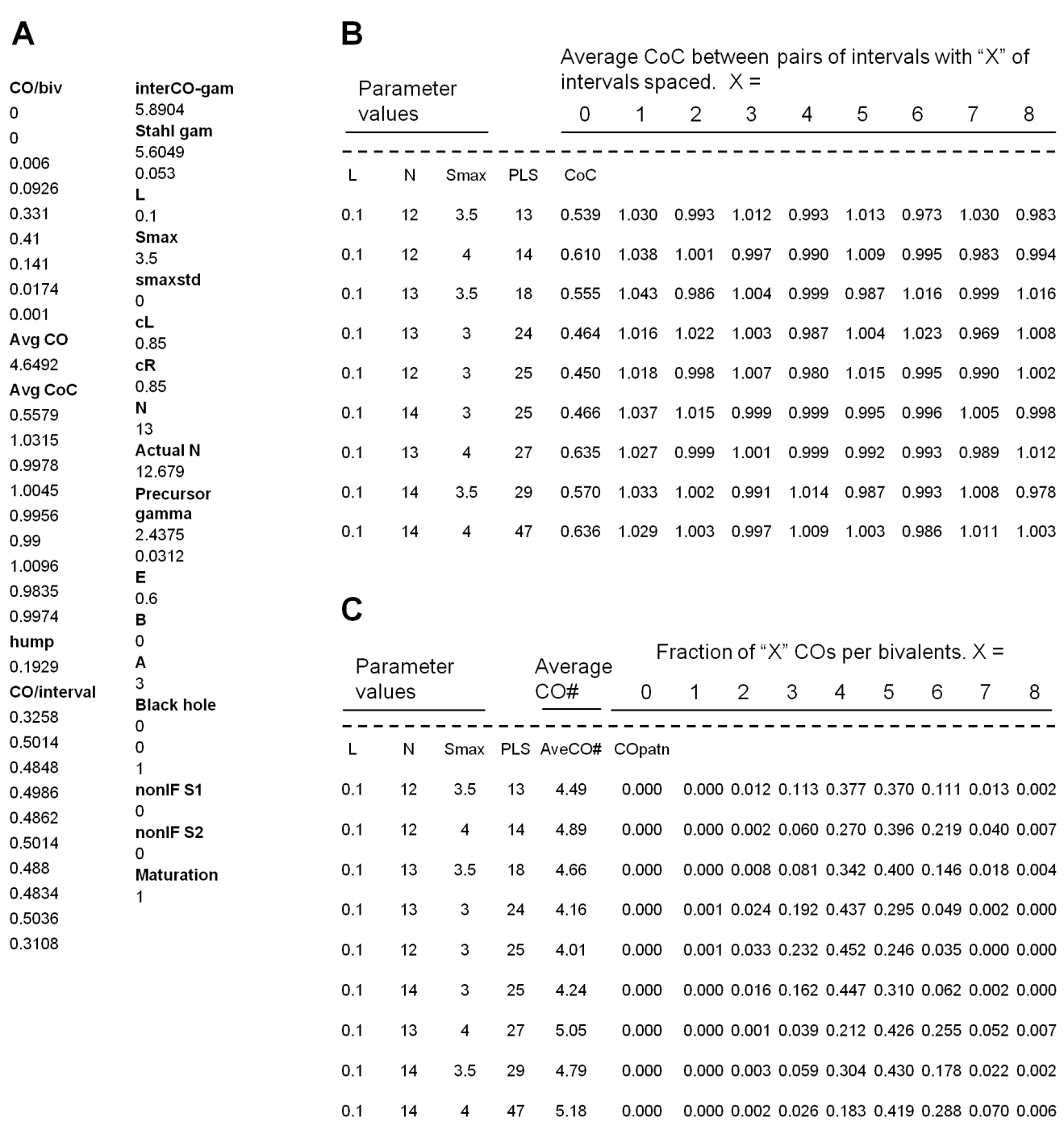

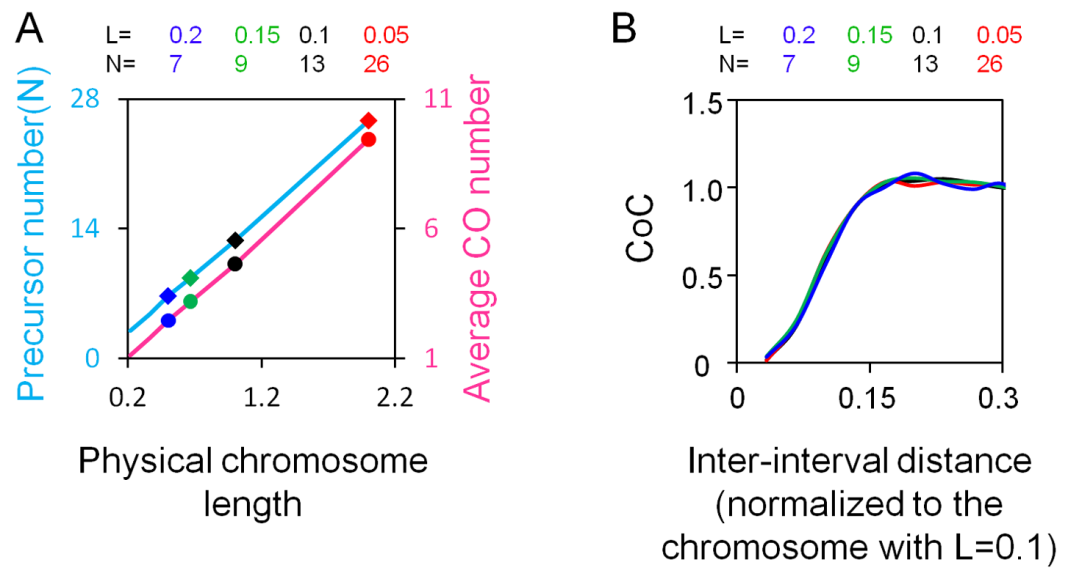


**Protocol Figure 4. Effects of physical chromosome length.** If different chromosomes are of the different physical lengths, the number of precursors is proportional to physical length as shown by experimental analysis. Thus, in BF simulations of such cases, (N) must be increased in proportion to chromosome length. Additionally, assuming that interference spreads over the same physical distance in both cases, as shown by experimental analysis for several examples, and given that the BF model normalizes every chromosome length to "1", the input value of (L) must be adjusted inversely to chromosome length such that it corresponds to the same physical distance in all cases. Simulations were performed in which (N) and (L) are varied coordinately as required to accommodate different physical chromosome lengths. Such simulations show that the number of COs varies in proportion to the number of precursors (Panel A) whereas CoC relationships are identical in all cases (Panel B).


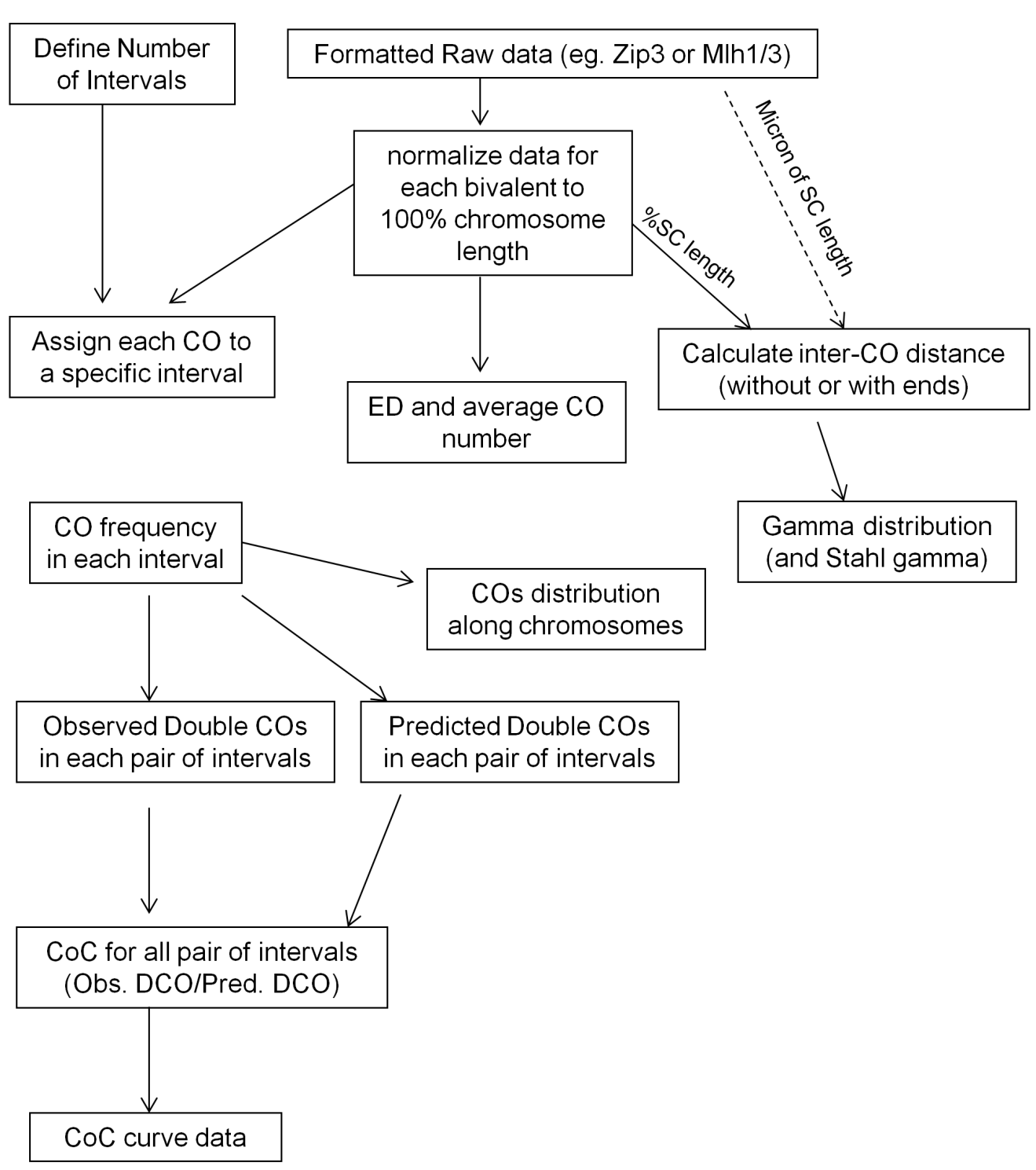


**Protocol Figure 5. Program work flow chart for Option A: Analysis of CO data.**

**Protocol Figure 6. Program work flow chart for Option B: A Single BF Simulation.**


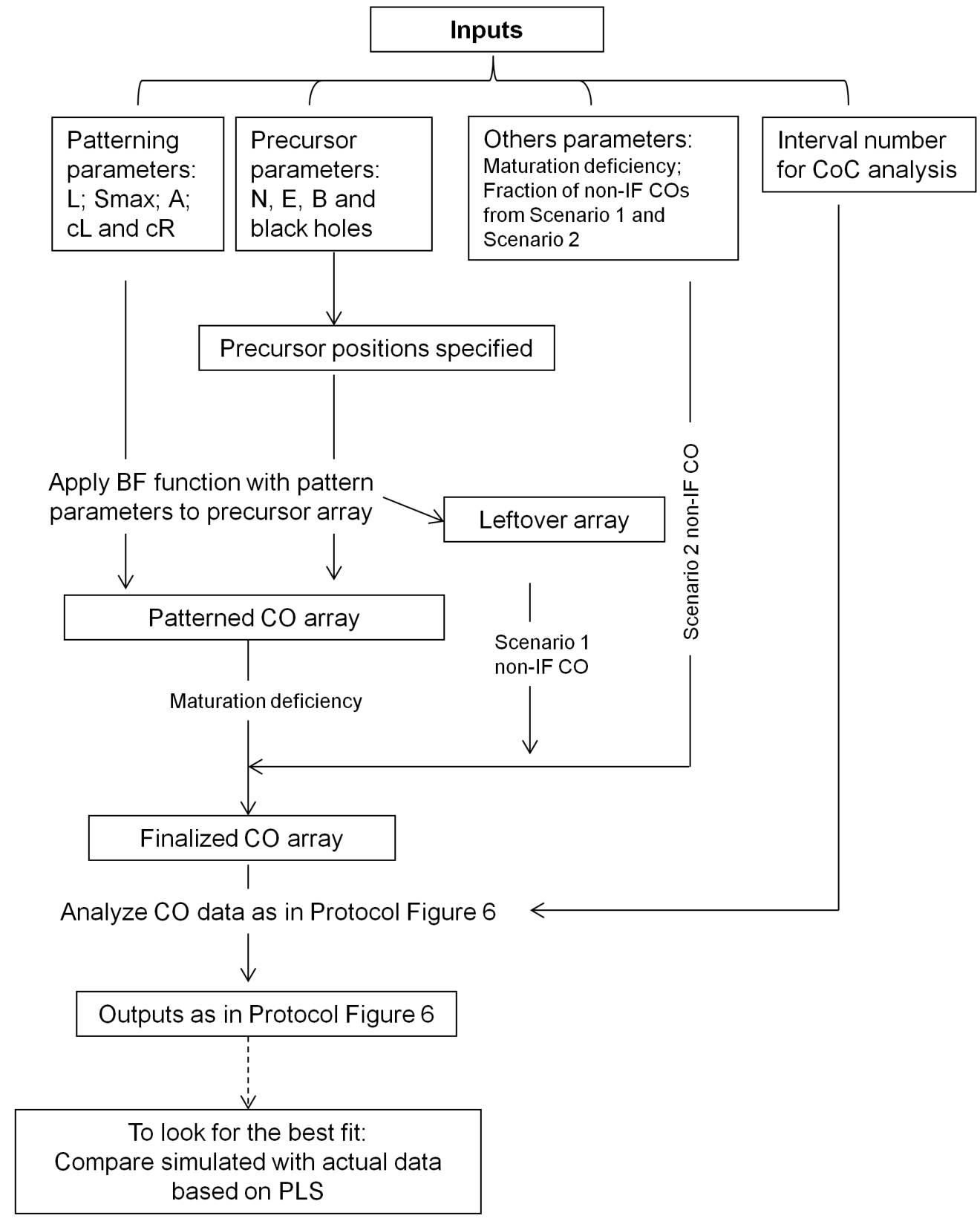

Supplement: Protocol S1 — Instructions for the BF program. (DOCX) [file pgen.1004042.s008.docx]
