# Supplementary material for: Crossover Patterning by the Beam-Film Model: Analysis and Implications
Source: PLoS Genet. 2014 Jan 30;10(1):e1004042. doi: 10.1371/journal.pgen.1004042 (PMC3907302; doi:10.1371/journal.pgen.1004042)
Supplement: Table S1 — Strains used in this study. (DOCX) [file pgen.1004042.s009.docx]

| Strains | Genotype |
| --- | --- |
| NKY4146 | ho::hisG/”, leu2/”, ura3/”, HMR::LacO-URA3/”, ZIP3-13myc::Hygromycin B/”, URA3::CYC1p-LacI-GFP/” |
| NKY4147 | ho::hisG/", leu2::hisG/", ura3(SmaI-PstI)/", ZIP3-13myc::Hygromycin B/", URA3::CYC1p-LacI-GFP/", scp1(Ch XV telomere)::LacO-LEU2/“ |
| NKY4148 | ho/", ura3/", ZIP3-13myc::Hygromycin B/", leu2::LacI-GFP::Clonat/", tel4::226xLacO::Kan/“ |
| NKY4149 | ho::hisG/", leu2::hisG/", ura3(SmaI-PstI)/", ZIP3-13myc::hygromycin B/+, URA3::CYC1p-LacI-GFP/", scp1(Ch XV telomere)::LacO-LEU2/", mlh1::KanMX/" |
| NKY4152 | ho/”, leu2::hisG/”, ura3(SmaI-PstI)/”, arg4-nsp/?, ZIP3-13myc::Hygromycin B/”, URA3::CYC1p-LacI-GFP/”, scp1(Ch XV telomere)::LacO-LEU2/”, tel1D::KanMX4/” |
| NKY4153 | ho/”, leu2::hisG/”, ura3(SmaI-PstI)/”,ZIP3-13myc::Hygromycin B/", URA3::CYC1p-LacI-GFP/", scp1(Ch XV telomere)::LacO-LEU2/", spo11-HA3His6::KanMX4/", tel1D::KanMX4/" |
| NKY4154 | ho/”, leu2::hisG/”, ura3(SmaI-PstI)/”, ZIP3-13myc::Hygromycin B/", URA3::CYC1p-LacI-GFP/ura3, scp1(Ch XV telomere)::LacO-LEU2/", spo11-HA3His6::KanMX4/" |
| NKY4155 | ho::hisG/", leu2::hisG/", ura3(SmaI-PstI)/", arg4-nsp?, ZIP3-13myc::Hygromycin B/", URA3::CYC1p-LacI-GFP/", scp1(Ch XV telomere)::LacO-LEU2/", spo11-HA3His6::KanMX4/spo11(D290A)-HA3His6::KAnMX4, Rec8-3HA::URA3/+ |
| NKY4156 | ho::hisG/", leu2::hisG/", ura3(SmaI-PstI)/", arg4-nsp?, ZIP3-13myc::Hygromycin B/", URA3::CYC1p-LacI-GFP/", scp1(Ch XV telomere)::LacO-LEU2/", spo11-HA3His6::KanMX4/spo11(Y135F)-HA3His6::KAnMX4, Rec8-3HA::URA3/+ |
| NKY4157 | ho::hisG/", leu2/", ura3/", pSE350 xHpaI, HMR::LacO Sherret-URA3/", ZIP3-13myc::Hygromycin B/", URA3::CYC1p-LacI-GFP/", tel1::KanMX4/" |
| NKY4158 | ho::hisG/", leu2::hisG/", ura3(SmaI-PstI)/", ZIP3-13myc::Hygromycin B/", URA3::CYC1p-LacI-GFP/", Rec8-3HA::URA3/", spo11-HA3His6::KanMX4/", HMR::LacO-URA3/" |
| NKY4159 | ho::hisG/", leu2::hisG/", ura3(SmaI-PstI)/", ZIP3-13myc::Hygromycin B/", URA3::CYC1p-LacI-GFP/", Rec8-3HA::URA3/+, spo11-HA3His6::KanMX4/spo11-HA3His6::KanMX4/spo11(Y135F)-HA3His6::KAnMX4, HMR::LacO-URA3/" |
